# Sex discrepancies in cancer research: a systematic review of prospective and retrospective investigations in lung, melanoma, and colorectal cancers

**DOI:** 10.3389/fgwh.2024.1445139

**Published:** 2024-11-11

**Authors:** Maria Díaz Rosario, Camille A. Vélez-Morell, Daniela V. Martinez

**Affiliations:** Universidad Central del Caribe School of Medicine, Bayamón, PR, United States

**Keywords:** cancer research, health disparities, sex differences, colorectal cancer, lung cancer, melanoma, retrospective, prospective

## Abstract

**Introduction:**

According to the latest Cancer Statistics, colorectal, lung, and melanoma are three of the most common cancers that affect both males and females. While males have consistently had a higher incidence and mortality rate in all three types of cancers, females have been shown to have better outcomes. Sex discrepancies in cancer research can impact the efficacy and effectiveness of novel drugs and diagnostic tools. Study results may not accurately represent how the treatment or diagnostic tool performs in the underrepresented sex. To comprehensively assess sex representation in top non-sex-specific cancer research, this systematic review aims to identify if there is equal representation of males and females in colorectal, lung, and melanoma cancer research.

**Methods:**

We explored retrospective and prospective clinical studies published in Pubmed from 2014 to 2023 to identify possible sex discrepancies in colorectal, lung, and melanoma cancer. MeSH terms were employed to retrieve relevant studies for each cancer type (colorectal, lung, melanoma). MeSH terms used include “lung cancer”, “melanoma”, and “colorectal cancer”, in combination with “trials”, “retrospective”, and “prospective”. Extracted data included study characteristics (author, year of publication), study design (prospective or retrospective), sample size, and the number of male and female participants.

**Results:**

The complete study population consisted of 515,003 patients, of which 275,231 (53%) were males and 237,488 (46%) were females. Specifically, retrospective studies included a total of 302,974 patients with 163,473 (54%) of them identifying as male and 139,072 (46%) patients identifying as female. While prospective studies included a total of 212,029 patients with 111,758 (53%) of these being male and 98,416 (46%) being female. Overall, male representation in the studies included in this systematic review was higher than female representation.

**Discussion:**

Disparities in representation were identified in colorectal cancer, lung cancer, and melanoma cancer studies underscoring the need for equitable inclusion of both sexes in cancer research to advance precision medicine and improve patient outcomes. Further exploration of the impact of sex, race, and socioeconomic status on study representation is warranted.

## Introduction

Cancer is one of the leading causes of death worldwide (1) and is projected to affect over two million people in the year 2024 (2); however, there is still no cure for this potentially deadly disease, making research essential to find new ways to treat, diagnose and prevent cancer (3). While there are over 100 types and subtypes of cancer, the latest cancer statistics published by the *American Cancer Society* highlight specific cancer types that have increased incidences (2). For instance, 48% of cancer incidences in men are made up of prostate, lung, and colorectal cancer, whereas 51% of cancer diagnoses in females consist of breast, lung, and colorectal cancer (2). Further examination of the top cancer diagnoses that are consistent in both sexes regardless of sex-specific cancer types reveals that lung cancer, colorectal cancer, and melanoma of the skin are responsible for the current top new cancer diagnosis cases (2). Because of the difference in the impact of cancer types on both sexes and the variability in incidence, survival, and mortality rates, research into these conditions must prioritize sex in their studies to ensure equal representation.

A closer look at sex distribution reveals that in 2023, the incidence of cancer and deaths was higher in males (>50%) than in females (<50%) (4). Similarly, the lifetime probability of being diagnosed with an invasive cancer was higher in men (41.6%) than in females (39.6%) (2). Male and female differences in genetic components, metabolic pathways, and sex hormones can affect the development, progression, and response to treatment for cancer (5). Not only can treatment response vary but also various types of cancer manifest differently in males and females (5). A closer look at colorectal cancer, lung cancer, and melanoma reveals differences in the estimated incidence and mortality in the latest statistics. In colorectal cancer, the incidence and mortality are expected to be higher in males, with an incidence of 81,540 and mortality of 28,700 vs. 71,270 and 24,310 in females respectively. In lung cancer, the incidence will be higher in females (118,270) than males (116,310), but the mortality will be higher in males (65,790) than in females (59,280). In melanoma, the incidence and mortality will be higher in males, with an incidence of 59,170 and mortality of 5,430. In contrast, the incidence in females will be 41,470 with a mortality of 2,860 (2). These disparities indicate the importance of adequate representation for males and females in cancer studies.

Sex disparities in cancer research could lead to gender bias, affecting treatment and patient outcomes and ultimately undermining the reliability and applicability of study findings (6). Should a specific sex be underrepresented in clinical studies, results of potential management and treatment options may not be accurate or yield similar results in the field. Therefore, to comprehensively assess sex representation in top non-sex-specific cancer research, the primary aim of this study is to investigate whether males and females are equally represented in colorectal, lung, and melanoma cancer by evaluating retrospective and prospective studies. Additionally, explore the potential implications of underrepresentation in the development of treatments and clinical outcomes and determine if there are significant differences.

## Methods

Using a systematic review methodology, we evaluated sex differences in prospective and retrospective studies concerning colorectal cancer, lung cancer, and melanoma. Utilizing PubMed as the primary data source, MeSH terms were employed to retrieve relevant studies for each cancer type (colorectal, lung, melanoma). MeSH terms used include “lung cancer”, “melanoma”, and “colorectal cancer”, in combination with “trials”, “retrospective”, and “prospective”. Research was limited to studies published within the last decade (2014–2023) to ensure relevance to current medical practice. Only studies involving adult populations (aged 18 years and older) were included in the analysis. Studies were included if they reported the number of male and female participants separately. Studies were excluded if they did not report sex-specific data or if they did not pertain to the specified cancer types. Systematic reviews were excluded from consideration to maintain focus on original prospective and retrospective studies. Data extraction was performed independently by three reviewers. Extracted data included study characteristics (author, year of publication), study design (prospective or retrospective), sample size, and the number of male and female participants. For colorectal cancer studies, there were 58 retrospective studies and 31 prospective studies. For lung cancer studies, there were 146 retrospective studies and 67 prospective studies for melanoma studies, there were 208 retrospective studies and 162 prospective studies. The extracted data were tabulated to summarize the number of male and female participants in each study. The representation of males and females in each study was calculated as a percentage of the total sample size. Studies were grouped by year, and the distribution of male and female participants over time was analyzed to identify any trends or changes. Additionally, risk ratios and mean differences between male and female representation in the studies were calculated to better understand if differences were significant.

## Results

### Overall study population

**Figure 1:**
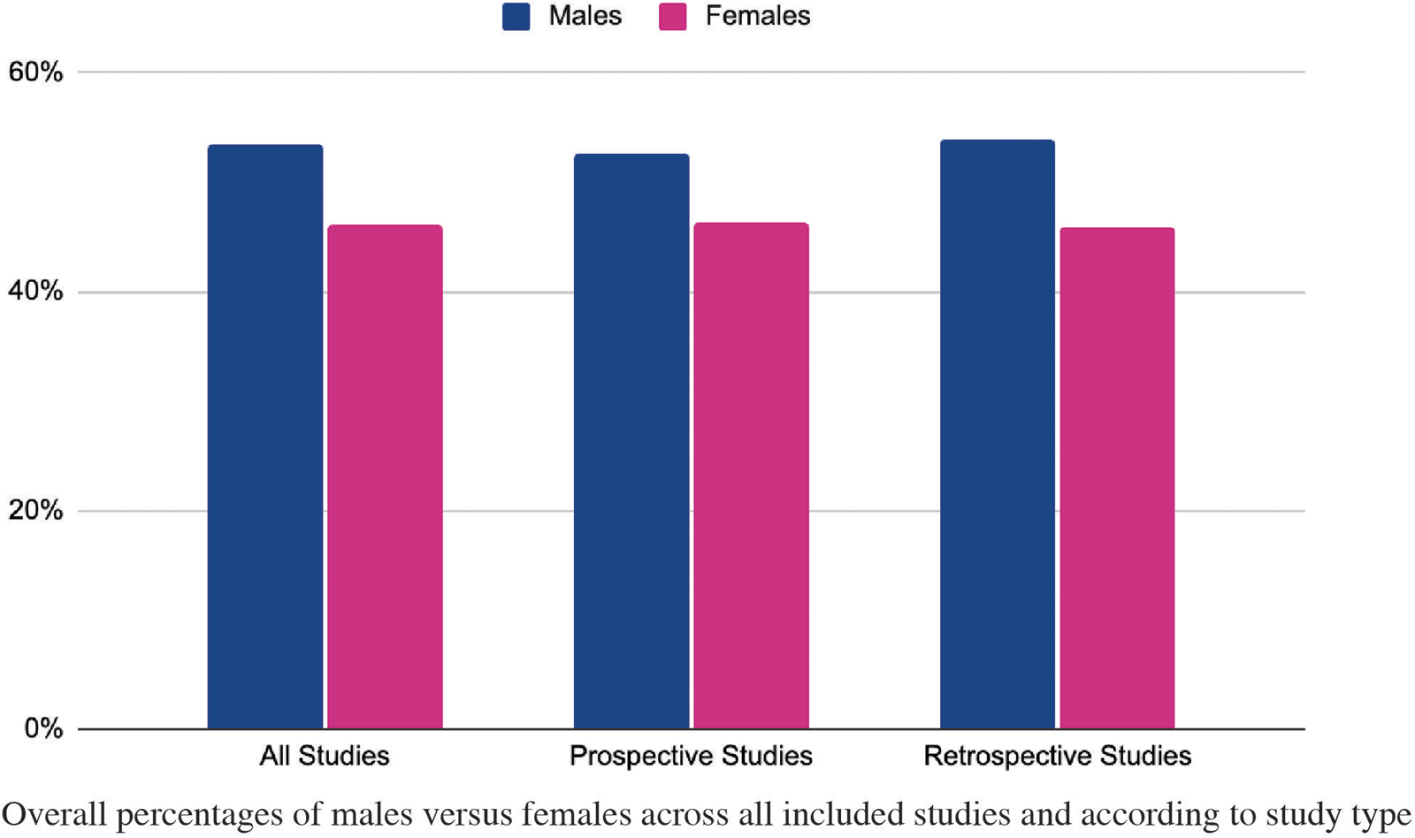
Total percentage of males vs. Females According To Study Type from 2014 to 2023.

The complete study population consisted of 515,003 patients, of which 275,231 (53%) were males and 237,488 (46%) were females (Figure 1). Similar results were obtained when analyzing the total number of patients according to cancer type in both retrospective and prospective studies. Briefly, retrospective studies included a total of 302,974 patients with 163,473 (54%) of them identifying as male and 139,072 (46%) patients identifying as female. While prospective studies included a total of 212,029 patients with 111,758 (53%) of these being male and 98,416 (46%) being female. Overall, male representation in the studies included in this systematic review was higher than female representation, with an overall risk ratio of 1.146, indicating a slight overrepresentation of males compared to females. The mean difference across all cancers was 6.29, further highlighting this disparity. Sex-specific data from retrospective and prospective lung, colorectal, and melanoma studies were analyzed to take a further look at representation in cancer-specific clinical trials with overall results summarized on Table 1. *Colorectal Cancer.*

**Table 1:** Summary of risk ratios and mean differences in sex representation across colorectal, lung, and melanoma cancer clinical trials.

Metric	Colorectal	Lung	Melanoma	Overall (all cancers)
Risk ratio (retrospective)	0.88	1.26	1.30	1.15
Risk ratio (prospective)	1.08	1.55	1.07	1.15
Risk ratio (overall)	0.94	1.33	1.18	1.15
Mean difference (retrospective)	−6.21	11.41	12.94	6.29
Mean difference (prospective)	3.72	21.45	3.60	6.29
Mean difference (overall)	−3.35	14.06	8.16	6.29

**Figure 2:**
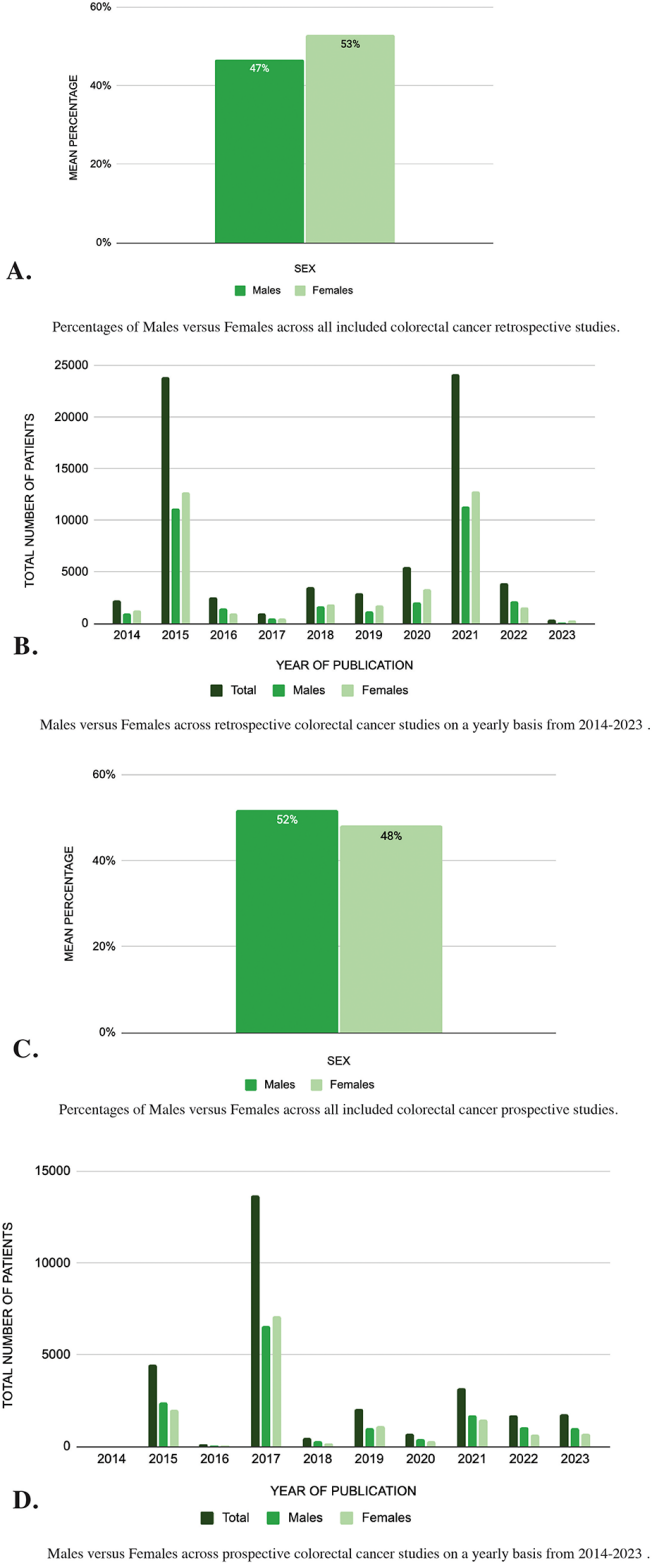
**(A–D)** Sex differences in colorectal cancer studies: analysis of pubMed publications from 2014 to 2023.

A total of 58 retrospective studies over a span of ten years were included in this systematic review (Supplementary Table S1A). These included a total of 70,072 patients with 32,758 (47%) males and 37,095 (53%) females (Figure 2A). When distributed across each year as shown by Figure 2B, 2015 and 2021 had a similar number of patients with an equal distribution between males and females at 47% and 53% respectively. Similarly, a higher female percentage was seen in the year with the least number of patients or 2023. During 2023, only 365 patients were identified in the included studies out of which 93 (25%) were male and 272 (75%) were female. The risk ratio for males in retrospective colorectal cancer studies was 0.883, indicating a slight underrepresentation of males compared to females. The mean difference in representation was −6.21, further supporting the higher representation of females in these studies. To further compare if the data from the prospective studies yielded similar results, an equivalent analysis was done.

A total of 31 prospective studies were included (Supplementary Table S1B) and these amounted to a total of 28,189 patients with 14,619 (52%) being males and 13,570 (48%) females (Figure 2C). The risk ratio for prospective colorectal cancer studies was 1.077, indicating a more balanced representation of males and females. The mean difference in prospective studies was 3.72, favoring a slight overrepresentation of males. When taking a closer look at the sex distribution by year as summarized in Figure 2D, the year 2017 included the largest number of patients with a total of 13,708 out of which 6,603 (48%) were male and 7,105 (52%) were female. In contrast, 2014 included the least number of patients with a total of 16 out of which 12 (75%) were male and 4 (25%) were female.

### Lung cancer

**Figure 3:**
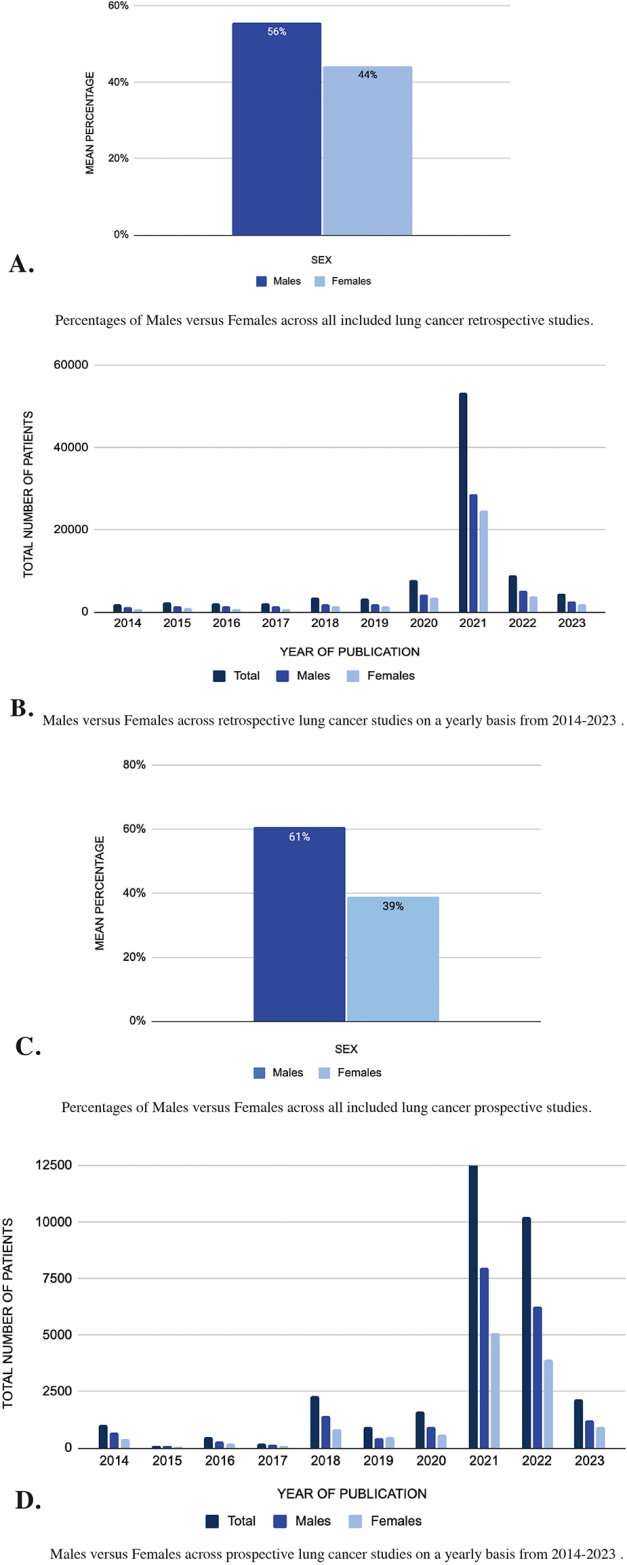
**(A–D)** Sex differences in lung cancer: analysis of pubMed publications from 2014 to 2023.

A total of 146 retrospective studies over a span of ten years were included in this systematic review (Supplementary Table S2A). A total of 89,879 patients were included, with 50,011 (56%) being males and 39,768 (44%) females (Figure 3A). The risk ratio for males in retrospective lung cancer studies was 1.257, indicating a significant overrepresentation of males compared to females. The mean difference in representation was 11.41, further highlighting the higher male participation in these studies. When examining the number of patients by year as seen in Figure 3B, it is evident that 2017 had the highest participation, totaling 8,947 participants, with 5,231 males (58%) and 3,716 females (42%). Instead, in 2014, although the total number of participants was lower compared to other years, the percentage of male patients (60%) was notably higher than the percentage of female patients (40%), indicating a potential imbalance in sex representation within that specific study year.

Conversely, in the 67 prospective lung cancer studies (Supplementary Table S2B), out of 32,166 patients, 19,527 (61%) were males, and 12,629 (39%) were females (Figure 3C). The risk ratio for prospective lung cancer studies was 1.546, showing an even larger disparity, with males being much more represented than females. The mean difference was 21.45, reflecting a notable overrepresentation of males in prospective studies. When examining the number of patients by year (Figure 3D), it is evident that 2021 had the highest participation, totaling 13,061 participants, with 7,963 males (61%) and 5,098 females (39%). Similarly, in 2018, despite a lower total number of participants compared to other years, the percentage of male patients (63%) was notably higher than the percentage of female patients (37%), suggesting a potential sex representation disparity within that specific study year.

These results reveal a consistent trend of gender disparities in both retrospective and prospective lung cancer studies, with males consistently comprising a higher proportion of patients compared to females.

### Melanoma cancer

**Figure 4:**
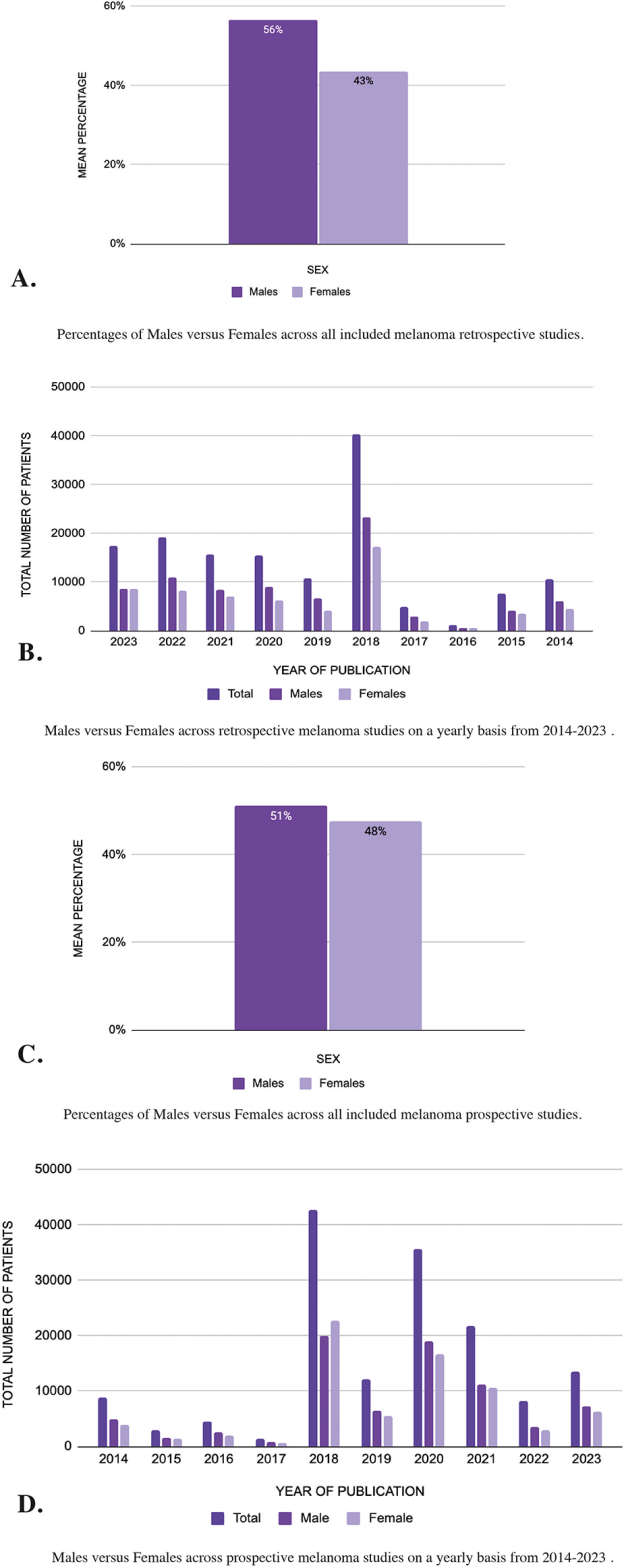
**(A–D)** Sex differences in melanoma cancer: analysis of pubMed publications from 2014 to 2023.

In the 208 retrospective melanoma studies that were analyzed **(**Supplementary Table S3A), there were a total of 143,023 patients of which 80,704 (56%) were male and 62,209 (43%) were female (Figure 4A). The risk ratio for males in retrospective melanoma studies was 1.297, indicating a considerable overrepresentation of males compared to females. The mean difference was 12.94, further emphasizing this disparity. When taking a closer look at each year in Figure 4B, 2018 included a higher number of patients (40,378) with 23,206 (57%) males and 17,172 (43%) females. Conversely, studies from 2016 included the lowest number of patients (1,154) with 645 (56%) males and 509 (44%).

Alternatively in the 162 prospective studies (Supplementary Table S3B), of the 151,674 patients 77,612 (51%) were male and 72,217 (48%) were female (Figure 4C). The risk ratio for prospective melanoma studies was 1.074, reflecting a balanced representation, though males were still slightly overrepresented. The mean difference in prospective melanoma studies was 3.60, showing a more modest imbalance compared to retrospective studies.

When observing the results for each year (Figure 4D), studies published in 2018 contain a higher number of patients of which 19,906 (47%) were male and 22,683 (53%) were female. However, in the year 2017, the studies included the least number of patients with 1,465 of which 891 (61%) were male and 574 (39%) were female.

Within the 370 studies analyzed, a consistent trend emerged indicating that males have a higher representation than females. This highlights the potential disparity in cancer research between males and females.

## Discussion

The results of this systematic review highlight significant sex disparities in cancer research across colorectal, lung, and melanoma studies. Both retrospective and prospective trials demonstrate an overall overrepresentation of males, as evidenced by an overall risk ratio of 1.146 and a mean difference of 6.29% across the three cancer types. These discrepancies raise important questions about the implications of such imbalances on the generalizability and efficacy of cancer treatments, particularly in the context of precision medicine.

### Colorectal cancer

Colorectal cancer is the third most diagnosed cancer in both males and females in the United States (2). In 2024, a total of 152,810 new diagnoses of colorectal cancer were recorded out of which 53% were in males and 47% were females (2); therefore, the incidence is slightly higher in males than in females. Similar sex distribution findings are seen in the estimated deaths due to colorectal cancer with males having 28,700 (54%) deaths compared to 24,310 (46%) in females (2). While females have a higher chance of survival, there has been an overall decrease in death rates due to an increase in screening (7). This results in early identification of colorectal cancers with better chances of effective treatment response.

Our prospective study findings echoed this sex distribution where males comprised a higher percentage of patients (52%) when compared to their female counterparts (48%). A similar fluctuation is observed when patients are broken down on a year-by-year basis. For instance, except for the years 2021, 2019, and 2017, all other years from 2014 to 2023 contain a higher representation of males in clinical studies than females. Within these, 2014 contained the highest sex disparity with 75% of study patients identified as male; However, this year included the least number of patients which could account for such differences in sex representation. Despite this, our study findings reflect a similar sex distribution when compared to cancer incidence statistics.

In contrast, total retrospective study findings revealed a higher female representation at 53% compared to 47% male representation. This marked difference could be due to a disparity in patient population size 2.49 times larger than the prospective studies included in this comparative analysis. Partly this could be due to the time needed to follow a population on a prospective basis compared to the accessibility of retrospective studies. Nevertheless, on a year-by-year basis, female representation was higher in all years except for the years 2022, 2017, and 2016. Most noticeably the year 2023 revealed that 75% of patients included in this study identified as female. In contrast, the year before, 2022, revealed that 55% of study patients were males; thus, highlighting that sex representation in these studies fluctuates every year.

While the overall incidence of colorectal cancer is higher in males (53% of cases), our study found that male representation in retrospective studies was lower than expected, with a risk ratio of 0.883. This underrepresentation of males, particularly in retrospective studies, could limit our understanding of how treatments affect male patients. In contrast, the prospective studies showed a risk ratio of 1.077, indicating more balanced sex representation. These disparities in representation across study types highlight the importance of ensuring equal sex representation in colorectal cancer research to fully capture sex-based differences in treatment response and outcomes.

For example, if we take a hypothetical colorectal cancer trial with 1,000 participants, based on the incidence rate of 53% males and 47% females, we would expect approximately 530 males and 470 females. However, given the risk ratio of 0.883 for males in retrospective studies, the actual number of male participants would be closer to 468, showing underrepresentation. Meanwhile, the risk ratio of 1.077 in prospective studies would result in approximately 571 male participants, reflecting a more balanced sex distribution. These numbers suggest that prospective studies better reflect the real-world incidence ratio compared to retrospective studies.

While a fluctuation in sex representation has been noted across years in retrospective colorectal cancer studies, it is evident that this disease predominantly afflicts males, resulting in a greater overall representation of males compared to females in the data. This trend may be linked to historical patterns of male participation in clinical studies due to concerns dating back to the 1900s about decreasing fertility or risking pregnancies if females were included in clinical trials (8). Additionally, certain bias from male researchers has been found to have impacted female representation in studies as the male race has been historically viewed as dominant (8). Although higher male representation can partly be explained by increased disease incidence, it's noteworthy that females exhibit a superior survival rate. Therefore, it is imperative to ensure equal sex representation in research to comprehensively grasp the variations in risk factors and potential treatment modalities according to sex, ultimately aiming for enhanced outcomes.

### Lung cancer

Lung cancer exhibits distinct sex disparities in both incidence and mortality rates. According to the American Cancer Society (ACS) journal, in the United States, the estimated new cases of lung cancer in 2024 were 108,710 for males and 86,580 for females (2). Similarly, mortality rates for lung cancer are higher in males, with an estimated 74,430 deaths among males compared to 59,020 deaths among females in 2024 (9). Furthermore, research indicates that females tend to have better survival rates compared to males, with a five-year survival rate of approximately 24% for females compared to 19% for males (9).

Across various years examined, retrospective lung cancer studies consistently reveal a higher percentage of male participants compared to females. This trend persists, with males comprising approximately 55%–65% of participants, while females account for 35%–45% (2). While this disparity remains consistent, there are notable fluctuations in the percentage breakdown between males and females from year to year. For example, in 2017, the percentage of male participants notably increased to 63% compared to 37% for females, indicating a pronounced sex imbalance in that specific year.

Similarly, prospective lung cancer studies also demonstrate sex disparities, with males consistently representing a higher percentage of patients compared to females. Across different years, males comprise approximately 58%–68% of patients, while females account for 32%–42% (2). Notably, variations in the percentage breakdown between males and females exist across different years. For instance, the percentage of male participants ranged from 47% in 2019 to 68% in 2015, reflecting varying degrees of sex imbalance within different study cohorts.

Although males have a higher incidence and mortality rate for lung cancer, our analysis reveals that males were overrepresented beyond what would be expected based on their incidence rates. In retrospective studies, the risk ratio for males was 1.257, and in prospective studies, it was 1.546, indicating significant male overrepresentation. This disproportionate male participation in trials could lead to treatment regimens that are skewed toward male physiology, potentially overlooking the factors that contribute to females’ better survival outcomes.

For instance, in a hypothetical lung cancer trial with 1,000 participants, the real-world incidence would predict approximately 556 males and 444 females. However, the risk ratio of 1.546 in prospective studies suggests that the actual number of male participants would be closer to 859, far exceeding the expected proportion based on incidence alone. This overrepresentation could bias treatment results toward male physiology, potentially underestimating treatment effects in females.

The higher incidence and mortality rates of lung cancer in males may partially explain the greater representation of males compared to females in both retrospective and prospective studies. Given that lung cancer predominantly affects males, it is understandable that studies would exhibit a larger proportion of male participants. Therefore, despite the higher incidence and mortality rates in males, females generally exhibit better survival rates. This underscores the importance of ensuring adequate representation of females in lung cancer studies to understand factors contributing to survival disparities and improve outcomes for all patients.

### Melanoma

In the United States, in 2024, about 100,640 new cases of cutaneous melanoma will be diagnosed, and 8,290 people are expected to die because of it (2). Worldwide, there are 232,100 primary cutaneous melanoma cases and around 55,500 related deaths (10). It most commonly occurs in older adults, but it is the third most common cancer in patients between 15 and 39 years old (11). In 2023, males had a higher rate of incidence and mortality. While melanoma is the least common type of skin cancer, it is the deadliest due to its ability to spread rapidly. Melanoma tumor cells have many factors contributing to rapid and unchecked cell proliferation and angiogenesis, contributing to its high metastatic rate (12). As a result of its high mortality and metastatic rate, many of the current clinical studies aim to optimize or discover new methods for the management and diagnosis of cutaneous melanoma and metastasis in the liver, brain, uvea, and lymph nodes.

Across retrospective studies published between 2014 and 2023, there is a higher percentage of male patients (56%) than females (43%). These results suggest an uneven distribution between male and female patients, which aligns with the fact that males have an overall higher incidence and mortality rate of melanoma. This difference was further pronounced in 2019, during which 62% of the patients were male.

Similarly, prospective studies also have a higher percentage of males (51%) than females (48%). However, the studies published in 2018 contain the highest number of patients, of which 47% are male. Interestingly, despite males historically having a higher incidence of melanoma, the prominence of females in the patient population of studies published in 2018 highlights the importance of considering sex disparities in melanoma cancer research. This observation prompts further investigation into potential factors influencing the distribution of patients in different study cohorts, which could ultimately contribute to more targeted and equitable approaches to melanoma prevention and therapies.

Melanoma studies showed significant overrepresentation of males, with a risk ratio of 1.297 in retrospective studies and 1.074 in prospective studies. While males have a higher incidence and mortality rate, this overrepresentation in trials goes beyond what would be expected based on incidence alone. For example, in a 1,000-participant melanoma trial, the expected number of male participants based on the incidence rate of 57% males would be 570 males. However, with a risk ratio of 1.297 in retrospective studies, the actual number of male participants would be approximately 739, showing significant overrepresentation. This could hinder understanding of the factors that contribute to females’ generally better outcomes in melanoma, potentially resulting in treatments that are more effective for males than females.

In general, there has been sex disparity in melanoma cancer studies in the last decade. Males have a higher percentage of participation in clinical trials involving novel diagnostic tools and possible treatments for both cutaneous and metastatic melanoma.

### Study limitations

Although this study represents a systematic review, there are limitations regarding the election process of studies that should be considered in the analysis. These limitations include the exclusive use of *Pubmed* as a source of relevant publications, as well as the absence of a minimum requirement for the number of patients included in the studies. Consequently, it may not represent all relevant studies or provide comprehensive information on sex representation in cancer research. Additionally, the number of studies published between 2014 and 2023 varies between colorectal, lung, and melanoma cancer. Therefore, some studies with very few patients may not accurately represent the population. While the study aimed to assess sex representation, it may not fully capture the complexity of gender diversity and inclusion, such as the representation of transgender and non-binary individuals in cancer research.

## Conclusion

The findings from this systematic review emphasize persistent sex disparities in cancer research, particularly in lung, colorectal, and melanoma cancer studies. Across both retrospective and prospective studies, males are consistently represented at a higher percentage than females, even after accounting for the higher male incidence and mortality rates for certain cancers. This overrepresentation of males may limit the generalizability of study results, potentially affecting the development of tailored treatment approaches and precision medicine strategies that account for sex-based differences in cancer progression and treatment response.

To address these disparities, several specific recommendations can be considered for future research. Policy changes at both the institutional and governmental levels should mandate balanced recruitment of male and female participants, ensuring that clinical trials reflect the actual incidence rates of the cancers studied. The NIH and the FDA have created policies and guidelines that promote the inclusion of women and ethnic minorities in clinical trials. In addition, Duke University and the FDA have created the Clinical Trials Transformation Initiative that has conducted investigations at an organizational level to implement changes that promote diversity and inclusion in clinical trials (13–15).

Targeted recruitment strategies should be developed to encourage increased female enrollment in cancer trials, particularly in cancers like lung and melanoma where males are historically overrepresented. This can be achieved through focused outreach programs, improved patient education about clinical trials, and addressing any socioeconomic or cultural barriers that may prevent female participation. Additionally, ensuring that trial protocols are designed with sex-specific variables in mind, such as hormonal influences or differences in drug metabolism, can further encourage participation by making trials more relevant to women.

Further studies are needed to assess the consequences of these sex imbalances on cancer prognosis and treatment efficacy. Future research designs should incorporate sex as a key variable in both recruitment and data analysis. This could include stratified randomization by sex in clinical trials or sex-based subgroup analyses to determine how treatment outcomes differ between males and females. Observational cohort studies and meta-analyses could also explore how these imbalances affect long-term cancer survival and recurrence rates.

A deeper exploration of the underlying factors contributing to these disparities is necessary to understand their full clinical implications. These factors may include biological differences such as variations in tumor biology, hormonal influences, and genetic factors that affect drug metabolism. Sociocultural and systemic biases, including historical trends of prioritizing male enrollment in clinical research, also play a significant role. Understanding how these factors contribute to sex disparities in cancer trials will allow for more equitable and effective treatment strategies moving forward.

Ultimately, addressing the sex imbalances identified in this review is critical for advancing precision medicine and improving cancer outcomes for both men and women. Future research must prioritize balanced representation, not only to enhance the scientific validity of studies but also to ensure that all patients benefit equally from advances in cancer care.

## Data Availability

The raw data supporting the conclusions of this article will be made available by the authors, without undue reservation.

## References

[1] Kim HI Lim H Moon A. Sex differences in cancer: epidemiology, genetics and therapy. Biomol Ther. (2018) 26(4):335–42. 10.4062/biomolther.2018.103 PMC6029678 29949843

[2] Siegel RL Giaquinto AN Jemal A. Cancer statistics, 2024. CA Cancer J Clin. (2024) 74(1):12–49. 10.3322/caac.21820 38230766

[3] American Cancer Society. Understanding Cancer Research Study Design and how to Evaluate Results. Hoboken, NJ: American Cancer Society (2013). Available online at: https://www.cancer.net/research-and-advocacy/introduction-cancer-research/understanding-cancer-research-study-design-and-how-evaluate-results (cited March 13, 2024).

[4] SEER. Cancer Disparities - Cancer Stat Facts. Atlanta: NIH- National Cancer Institute. (2022). Available online at: https://seer.cancer.gov/statfacts/html/.html (cited March 13, 2024).

[5] Li CH Haider S Shiah YJ Thai K Boutros PC. Sex differences in cancer driver genes and biomarkers. Cancer Res. (2018) 78(19):5527–37. 10.1158/0008-5472.CAN-18-0362 30275052

[6] Magni F Jhala M Harky A. Gender disparities in concerns of cancer research participation during COVID-19 climate. Cancer Control. (2021) 28:10732748211024214. 10.1177/10732748211024214 34126789 PMC8209786

[7] American Cancer Society. Colorectal Cancer Statistics | how Common is Colorectal Cancer? Hoboken, NJ: American Cancer Society (2024). Available online at: https://www.cancer.org/cancer/types/colon-rectal-cancer/about/key-statistics.html Viewless (cited April 6, 2024).

[8] Merone L Tsey K Russell D Nagle C. Sex inequalities in medical research: a systematic scoping review of the literature. Womens Health Rep. (2022) 3(1):49–59. 10.1089/whr.2021.0083 PMC8812498 35136877

[9] Siegel RL Miller KD Wagle NS Jemal A. Cancer statistics, 2023. CA Cancer J Clin. (2023) 73(1):17–48. 10.3322/caac.21763 36633525

[10] Schadendorf D Van Akkooi ACJ Berking C Griewank KG Gutzmer R Hauschild A Melanoma. Lancet. (2018) 392(10151):971–84. 10.1016/S0140-6736(18)31559-9 30238891

[11] Dzwierzynski WW. Melanoma risk factors and prevention. Clin Plast Surg. (2021) 48(4):543–50. 10.1016/j.cps.2021.05.001 34503715

[12] Braeuer RR Watson IR Wu C Mobley AK Kamiya T Shoshan E Why is melanoma so metastatic? Pigment Cell Melanoma Res. (2014) 27(1):19–36. 10.1111/pcmr.12172 24106873

[13] NIH. Clinical Trial Diversity | FDA. Bethesda, MD: FDA (2024). Available online at: https://www.fda.gov/953consumers/minority-health-and-health-equity/clinical-trial-diversity (cited September 28, 2024).

[14] Grey P McNicol LA Palagi S Kelty M Hanna E McGowan J NIH Policy and Guidelines on the Inclusion of Women and Minorities as Subjects 955 in Clinical Research | Grants & Funding. Bethesda, MD: NIH- Grants and Funding (2024). Available online at: https://grants.nih.gov/956policy-and-compliance/policy-topics/inclusion/women-and-minorities/guideline (cited September 28, 2024).

[15] Corneli A Hanlen-Rosado E McKenna K Araojo R Corbett D Vasisht K Enhancing diversity and inclusion in clinical trials. Clin Pharmacol Ther. (2023). 113(3):489–99. 10.1002/cpt.2819 36628990

