# Supplementary material for: Sex discrepancies in cancer research: a systematic review of prospective and retrospective investigations in lung, melanoma, and colorectal cancers
Source: Front Glob Womens Health. 2024 Nov 11;5:1445139. doi: 10.3389/fgwh.2024.1445139 (PMC11586390; doi:10.3389/fgwh.2024.1445139)
Supplement: Supplementary file 1 [file Table1.docx]

**Supplemental Table S1A.** Colorectal Retrospective Studies Included in Systematic Review

| Author, year | Title | Male/Female |
| --- | --- | --- |
| (1) Tinguely, et al., 2023 | A prospective multicentre trial on survival after Microwave Ablation Versus Resection for Resectable Colorectal liver metastases (MAVERRIC) | 488/293 |
| (2) King, et al., 2023 | Safety and feasibility of same-day discharge after endoscopic submucosal dissection: a Western multicenter prospective cohort study | 474/357 |
| (3) Meyer, et al., 2023 | Implementation of totally robotic right hemicolectomy: lessons learned from a prospective cohort | 28/32 |
| (4) Van der Does de Willebois, et al., 2023 | Endoscopic Recurrence or Anastomotic Wound Healing Phenomenon after Ileocolic Resection for Crohn's Disease: The Challenges of Accurate Endoscopic Scoring | 37/51 |
| (5) Cleary, et al., 2022 | Intracorporeal and extracorporeal anastomosis for robotic-assisted and laparoscopic right colectomy: short-term outcomes of a multi-center prospective trial | 135/145 |
| (6) Emmons, et al. 2022 | Survival and Toxicities after ^90^Y Transarterial Radioembolization of Metastatic Colorectal Cancer in the RESIN Registry | 298/200 |
| (7) Bojesen, et al. 2022 | Fit for Surgery-feasibility of short-course multimodal individualized prehabilitation in high-risk frail colon cancer patients prior to surgery | 4/4 |
| (8) Ohue, et al. 2022 | Preoperative and postoperative prognostic factors of patients with stage II/III lower rectal cancer without neoadjuvant therapy in the clinical trial (JCOG0212) | 450/213 |
| (9) Rouanet, et al., 2022 | Tailored Strategy for Locally Advanced Rectal Carcinoma (GRECCAR 4): Long-term Results From a Multicenter, Randomized, Open-Label, Phase II Trial | 90/43 |
| (10) Falt, et al., 2022 | Endoscopic full-thickness resection versus endoscopic submucosal dissection in the treatment of colonic neoplastic lesions ≤ 30 mm-a single-center experience | 77/25 |
| (11) Reuter, et al., 2021 | Using Patient-Generated Health Data From Twitter to Identify, Engage, and Recruit Cancer Survivors in Clinical Trials in Los Angeles County: Evaluation of a Feasibility Study | 202/235 |
| (12) Sinicrope, et al., 2021 | Prognostic variables in low and high risk stage III colon cancers treated in two adjuvant chemotherapy trials | 1527/1243 |
| (13) Bremholm, et al., 2020 | Pressurized IntraPeritoneal Aerosol Chemotherapy (PIPAC)-directed treatment of peritoneal metastasis in end-stage colo-rectal cancer patients | 13/11 |
| (14) Imai, et al., 2020 | A risk-prediction model for en bloc resection failure or perforation during endoscopic submucosal dissection of colorectal neoplasms | 403/270 |
| (15) Kosumi, et al. 2019 | Prognostic association of PTGS2 (COX-2) over-expression according to BRAF mutation status in colorectal cancer: Results from two prospective cohorts and CALGB 89803 (Alliance) trial | 524/676 |
| (16) Péron, et al., 2019 | The location of the primary colon cancer has no impact on outcomes in patients undergoing cytoreductive surgery for peritoneal metastasis | 387/409 |
| (17) Coevoet, et al., 2019 | Quality of life of patients with a colonic interposition postoesophagectomy | 66/14 |
| (18) Suhardja, et al, 2018 | Comparison of the Thunderbeat and Other Energy Devices in Laparoscopic Colorectal Resection: A Single-Center Experience | 56/58 |
| (19) Hofmann, et al., 2018 | Prognostic value of radiologically enlarged lymph nodes in patients with metastatic colorectal cancer: Subgroup findings of the randomized, open-label FIRE-3/AIO KRK0306 trial | 239/100 |
| (20) Atkin, et al., 2017 | Is whole-colon investigation by colonoscopy, computerised tomography colonography or barium enema necessary for all patients with colorectal cancer symptoms, and for which patients would flexible sigmoidoscopy suffice? A retrospective cohort study | 3080/4300 |
| (21) Cerdán Santacruz, et al., 2017 | **Laparoscopy may decrease morbidity and length of stay after elective colon cancer resection, especially in frail patients: results from an observational real-life study** | 1798/1170 |
| (22) Jia, et al., 2016 | Water Exchange Method Significantly Improves Adenoma Detection Rate: A Multicenter, Randomized Controlled Trial | 1685/1618 |
| (23)Sasaki, et al., 2017 | Postoperative Chemoradiotherapy After Local Resection for High-Risk T1 to T2 Low Rectal Cancer: Results of a Single-Arm, Multi-Institutional, Phase II Clinical Trial | 40/17 |
| (24) Ali, et al., 2016 | Lean body mass as an independent determinant of dose-limiting toxicity and neuropathy in patients with colon cancer treated with FOLFOX regimens | 73/65 |
| (25) Pentheroudakis, et al., 2015 | Immune response gene expression in colorectal cancer carries distinct prognostic implications according to tissue, stage and site: a prospective retrospective translational study in the context of a hellenic cooperative oncology group randomised trial | 244/197 |
| (26) Klare, et al., 2015 | Patient age and duration of colonoscopy are predictors for adenoma detection in both proximal and distal colon | 285/266 |
| (27) Steffens, et al., 2015 | Observational study of adjuvant therapy with capecitabine in colon cancer | 820/657 |
| (28) Nachiappan, et al., 2015 | The impact of anastomotic leak and its treatment on cancer recurrence and survival following elective colorectal cancer resection | 590/458 |
| (29) Bakker, et al., 2015 | Eight years of experience with Enhanced Recovery After Surgery in patients with colon cancer: Impact of measures to improve adherence | 431/385 |
| (30) Mussetto, et al., 2015 | Split dosing with a low-volume preparation is not inferior to split dosing with a high-volume preparation for bowel cleansing in patients with a history of colorectal resection: a randomized trial | 63/57 |
| (31) Sahebally, et al., 2014 | Short-term outcomes following the use of self-expanding metallic stents in acute malignant colonic obstruction--a single centre experience | 12/4 |

**Supplemental Table S1B.** Colorectal Prospective Studies Included in Systematic Review

| Author, year | Title | Male/female |
| --- | --- | --- |
| (32) Marolleau, et al., 2023 | Complete pathological response after chemotherapy or immune checkpoint inhibitors in deficient MMR metastatic colorectal cancer: Results of a retrospective multicenter study | 46/42 |
| (33) Kou, et al., 2023 | Immune checkpoint inhibitor-induced colitis with endoscopic evaluation in Chinese cancer patients: a single-centre retrospective study | 22/3 |
| (34) Mu, et al., 2023 | A retrospective evaluation of short-term results from colonic stenting as a bridge to elective surgery versus emergency surgery for malignant colonic obstruction | 25/8 |
| (35) Hamada, et al., 2023 | Risk Factors Associated with Painful Colonoscopy and Prolonged Cecal Intubation Time in Female Patients | 0/219 |
| (36) Samalavicius, et al., 2022 | Robotic colorectal surgery using the Senhance^®^ robotic system: a single center experience | 29/28 |
| (37) Baker, et al., 2022 | Are the current colonoscopy recommendations for interval surveillance in patients with polyps enough? Machine learning-augmented propensity score cohort analysis of 1840 patients | 1149/691 |
| (38) Cheng, et al., 2022 | Colonoscopic Ultrasound-Guided Fine-Needle Aspiration Using a Curvilinear Array Transducer: A Single-Center Retrospective Cohort Study | 0/13 |
| (39) Duong, et al., 2022 | Evaluation of the polyp-based resect and discard strategy: a retrospective study | 274/270 |
| (40) Ghareeb, et al., 2022 | The "terminal line": a novel sign for the identification of distal mesorectum end during TME for rectal cancer | 59/40 |
| (41) Hrebinko, et al,, 2022 | Transanal excision with adjuvant therapy for pT1N0 rectal tumors with high-risk features offers equivalent survival to radical resection: A National Cancer Database analysis | 656/500 |
| (42) El-Sharkawy, et al., 2021 | Minimally invasive surgery for T4 colon cancer is associated with better outcomes compared to open surgery in the National Cancer Database | 10144/11854 |
| (43) Alkrekshi, et al., 2021 | Safety of Immune Checkpoint Inhibitors in Patients with Cancer and Hepatitis C Virus Infection | 30/10 |
| (44) Oki, et al., 2021 | Evaluation of a 55-gene classifier as a prognostic biomarker for adjuvant chemotherapy in stage III colon cancer patients | 98/92 |
| (45) Halamkove, et al., 2021 | Second primary malignancies in colorectal cancer patients | 724/450 |
| (46) Renelus, et al., 2021 | Short-term aspirin and statin chemoprophylaxis did not reduce the risk of developing advanced adenomatous polyps in Black patients | 335/411 |
| (47) Milone, et al., 2020 | **Mid-transverse colon cancer and extended versus transverse colectomy: Results of the Italian society of surgical oncology colorectal cancer network (SICO CCN) multicenter collaborative study** | 192/196 |
| (48) Mao, et al., 2020 | **Concordance Between Watson for Oncology and Multidisciplinary Teams in Colorectal Cancer: Prognostic Implications and Predicting Concordance** | 109/66 |
| (49) Roberto, et al., 2020 | **Evaluation of Prognostic Factors for Survival in Transverse Colon Cancer** | 59/38 |
| (50) Crippa, et al., 2020 | **Long-term Oncological Outcomes Following Anastomotic Leak in Rectal Cancer Surgery** | 527/260 |
| (51) Miller, et al., 2020 | **Stage I Squamous Cell Carcinoma of the Anus: Is Radiation Therapy Alone Sufficient Treatment?** | 1136/2703 |
| (52)Shi, et al., 2020 | **Short-Term Outcomes of Three-Port Laparoscopic Right Hemicolectomy Versus Five-Port Laparoscopic Right Hemicolectomy: With a Propensity Score Matching Analysis** | 76/92 |
| (53) Kawasaki, et al., 2019 | **Clinical usefulness of magnifying colonoscopy for the diagnosis of ulcerative colitis-associated neoplasia** | 8/9 |
| (54) Numata, et al., 2019 | **Laparoscopic surgery in patients diagnosed with clinical N2 colon cancer** | 98/64 |
| (55) David, et al., 2019 | **Confounders in Adenoma Detection at Initial Screening Colonoscopy: A Factor in the Assessment of Racial Disparities as a Risk for Colon Cancer** | 788/1437 |
| (56) Woff, et al., 2019 | **Validation of Metabolically Active Tumor Volume and Total Lesion Glycolysis as 18F-FDG PET/CT–derived Prognostic Biomarkers in Chemorefractory Metastatic Colorectal Cancer** | 127/97 |
| (57) Bhatt, et al, 2019 | **Patients with extensive regional lymph node involvement (pN2) following potentially curative surgery for colorectal cancer are at increased risk for developing peritoneal metastases: a retrospective single-institution study** | 127/114 |
| (58) Carriles, et al., 2019 | **Trifluridine/Tipiracil (TAS-102) for refractory metastatic colorectal cancer in clinical practice: a feasible alternative for patients with good performance status** | 49/35 |
| (59) Pelz, et al., 2018 | **Laparoscopic right-sided colon resection for colon cancer-has the control group so far been chosen correctly?** | 149/130 |
| (60) Kang, et al., 2018 | **Multicenter, randomized single-port versus multiport laparoscopic surgery (SIMPLE) trial in colon cancer: an interim analysis** | 101/80 |
| (61) Dahdaleh, et al., 2018 | **Obstruction predicts worse long-term outcomes in stage III colon cancer: A secondary analysis of the N0147 trial** | 729/814 |
| (62) Manzenreiter, et al., 2018 | **A proposal for a tailored approach to diverting ostomy for colorectal anastomosis** | 95/72 |
| (63) Sage, et al., 2018 | **Laparoscopic delayed coloanal anastomosis without diverting ileostomy for low rectal cancer surgery: 85 consecutive patients from a single institution** | 69/16 |
| (64) Fritz, et al., 2018 | **Prolonged Cecal Insertion Time Is Not Associated with Decreased Adenoma Detection When a Longer Withdrawal Time Is Achieved** | 544/759 |
| (65) Kim, et al., 2017 | **Outcome of bridge to surgery stenting for obstructive left colon cancer** | 80/78 |
| (66) Burgess, et al., 2017 | **Deep mural injury and perforation after colonic endoscopic mucosal resection: a new classification and analysis of risk factors** | 412/390 |
| (67) Pecorelli, et al., 2016 | **Ten-year outcomes following laparoscopic colorectal resection: results of a randomized controlled trial** | 345/259 |
| (68) Sharara, et al., 2016 | **Body mass index and quality of bowel preparation: Real life vs. clinical trials** | 283/258 |
| (69) Grade, et al., 2016 | **[Robotic Rectal Cancer Surgery]** | 136/66 |
| (70) Plumb, et al., 2016 | **Appearances of screen-detected versus symptomatic colorectal cancers at CT colonography** | 180/53 |
| (71) Lapeyre-Prost, et al., 2016 | **Feasibility of Capecitabine and Oxaliplatin Combination Chemotherapy Without Central Venous Access Device in Patients With Stage III Colorectal Cancer** | 123/78 |
| (72) Anjos, et al., 2016 | **Semiquantitative Volumetry by Sequential PET/CT May Improve Prediction of Complete Response to Neoadjuvant Chemoradiation in Patients With Distal Rectal Cancer** | 45/45 |
| (73) Imai, et al., 2016 | **Preoperative indicators of failure of en bloc resection or perforation in colorectal endoscopic submucosal dissection: implications for lesion stratification by technical difficulties during stepwise training** | 403/270 |
| (74) An, et al., 2015 | **T4 stage and preoperative anemia as prognostic factors for the patients with colon cancer treated with adjuvant FOLFOX chemotherapy** | 112/84 |
| (75) Jeong, et al., 2015 | **Association Between Time (Initiation and Length) and Oncological Outcomes for the Patients with Colon Cancer Treated with Adjuvant Chemotherapy** | 246/178 |
| (76) Vogelaar, et al., 2015 | **Epidural analgesia associated with better survival in colon cancer** | 288/290 |
| (77) Mistrangelo, et al., 2015 | **Laparoscopic versus open resection for transverse colon cancer** | 55/68 |
| (78) Wang, et al., 2015 | **Efficacy and safety of neoadjuvant intensity-modulated radiotherapy with concurrent capecitabine for locally advanced rectal cancer** | 178/82 |
| (79) Ross, et al., 2015 | **Detection rates of premalignant polyps during screening colonoscopy: time to revise quality standards?** | 1003/1830 |
| (80) Thirunavukarusu, et al., 2015 | **Effect of Incorporation of Pretreatment Serum Carcinoembryonic Antigen Levels Into AJCC Staging for Colon Cancer on 5-Year Survival** | 7936/8683 |
| (81) Kim, et al., 2015 | **ERCC1 as a Predictive Marker for FOLFOX Chemotherapy in an Adjuvant Setting** | 92/74 |
| (82) Daniels, et al., 2015 | **Yield of colonoscopy after recent CT-proven uncomplicated acute diverticulitis: a comparative cohort study** | 917/910 |
| (83) Degener, et al., 2015 | **Long-term experience of hyperbaric oxygen therapy for refractory radio- or chemotherapy-induced haemorrhagic cystitis** | 12/3 |
| (84) Lee, et al., 2015 | **Diminutive polyps among black and Latino populations undergoing screening colonoscopy: evidence supporting a resect and discard approach** | 196/441 |
| (85) Maeda, et al., 2015 | **Efficacy of intracorporeal reinforcing sutures for anastomotic leakage after laparoscopic surgery for rectal cancer** | 118/83 |
| (86) Hugen, et al., 2014 | **Metastatic pattern in colorectal cancer is strongly influenced by histological subtype** | 663/975 |
| (87) Maitra, et al., 2014 | **Results of laparoscopic colorectal surgery from a national training center** | 258/250 |
| (88) Amato, et al., 2014 | **One-step mini-invasive treatment of abdominal aortic-iliac aneurysm associated with colo-rectal cancer** | 56/6 |
| (89) Sammartino, et al., 2014 | **Long-term results after proactive management for locoregional control in patients with colonic cancer at high risk of peritoneal metastases** | 47/28 |

**References**

1. Tinguely P, Ruiter SJS, Engstrand J, de Haas RJ, Nilsson H, Candinas D, et al. A prospective multicentre trial on survival after Microwave Ablation VErsus Resection for Resectable Colorectal liver metastases (MAVERRIC). Eur J Cancer Oxf Engl 1990. 2023 Jul;187:65–76.

2. King W, Draganov P, Gorrepati VS, Hayat M, Aihara H, Karasik M, et al. Safety and feasibility of same-day discharge after endoscopic submucosal dissection: a Western multicenter prospective cohort study. Gastrointest Endosc. 2023 Jun;97(6):1045–51.

3. Meyer J, Wijsman J, Crolla R, van der Schelling G. Implementation of totally robotic right hemicolectomy: lessons learned from a prospective cohort. J Robot Surg. 2023 Oct;17(5):2315–21.

4. van der Does de Willebois EML, Duijvestein M, Wasmann KA, D’Haens GRAM, van der Bilt JDW, Mundt MW, et al. Endoscopic Recurrence or Anastomotic Wound Healing Phenomenon after Ileocolic Resection for Crohn’s Disease: The Challenges of Accurate Endoscopic Scoring. J Crohns Colitis. 2023 May 3;17(5):693–9.

5. Cleary RK, Silviera M, Reidy TJ, McCormick J, Johnson CS, Sylla P, et al. Intracorporeal and extracorporeal anastomosis for robotic-assisted and laparoscopic right colectomy: short-term outcomes of a multi-center prospective trial. Surg Endosc. 2022 Jun;36(6):4349–58.

6. Emmons EC, Bishay S, Du L, Krebs H, Gandhi RT, Collins ZS, et al. Survival and Toxicities after 90Y Transarterial Radioembolization of Metastatic Colorectal Cancer in the RESIN Registry. Radiology. 2022 Oct;305(1):228–36.

7. Bojesen RD, Jørgensen LB, Grube C, Skou ST, Johansen C, Dalton SO, et al. Fit for Surgery-feasibility of short-course multimodal individualized prehabilitation in high-risk frail colon cancer patients prior to surgery. Pilot Feasibility Stud. 2022 Jan 21;8(1):11.

8. Ohue M, Fujita S, Mizusawa J, Kanemitsu Y, Hamaguchi T, Tsukamoto S, et al. Preoperative and postoperative prognostic factors of patients with stage II/III lower rectal cancer without neoadjuvant therapy in the clinical trial (JCOG0212). Jpn J Clin Oncol. 2022 Feb 5;52(2):114–21.

9. Rouanet P, Rullier E, Lelong B, Maingon P, Tuech JJ, Pezet D, et al. Tailored Strategy for Locally Advanced Rectal Carcinoma (GRECCAR 4): Long-term Results From a Multicenter, Randomized, Open-Label, Phase II Trial. Dis Colon Rectum. 2022 Aug 1;65(8):986–95.

10. Falt P, Zapletalová J, Urban O. Endoscopic full-thickness resection versus endoscopic submucosal dissection in the treatment of colonic neoplastic lesions ≤ 30 mm-a single-center experience. Surg Endosc. 2022 Mar;36(3):2062–9.

11. Reuter K, Angyan P, Le N, Buchanan TA. Using Patient-Generated Health Data From Twitter to Identify, Engage, and Recruit Cancer Survivors in Clinical Trials in Los Angeles County: Evaluation of a Feasibility Study. JMIR Form Res. 2021 Nov 26;5(11):e29958.

12. Prognostic variables in low and high risk stage III colon cancers treated in two adjuvant chemotherapy trials - PubMed [Internet]. [cited 2024 Apr 1]. Available from: https://pubmed.ncbi.nlm.nih.gov/33341444/

13. Ellebæk SB, Graversen M, Detlefsen S, Lundell L, Fristrup CW, Pfeiffer P, et al. Pressurized IntraPeritoneal Aerosol Chemotherapy (PIPAC)-directed treatment of peritoneal metastasis in end-stage colo-rectal cancer patients. Pleura Peritoneum. 2020 Jun 1;5(2):20200109.

14. A risk-prediction model for en bloc resection failure or perforation during endoscopic submucosal dissection of colorectal neoplasms - PubMed [Internet]. [cited 2024 Apr 1]. Available from: https://pubmed.ncbi.nlm.nih.gov/31883411/

15. Kosumi K, Hamada T, Zhang S, Liu L, da Silva A, Koh H, et al. Prognostic association of PTGS2 (COX-2) over-expression according to BRAF mutation status in colorectal cancer: Results from two prospective cohorts and CALGB 89803 (Alliance) trial. Eur J Cancer Oxf Engl 1990. 2019 Apr;111:82–93.

16. Péron J, Mercier F, Tuech JJ, Younan R, Sideris L, Gelli M, et al. The location of the primary colon cancer has no impact on outcomes in patients undergoing cytoreductive surgery for peritoneal metastasis. Surgery. 2019 Feb;165(2):476–84.

17. Quality of life of patients with a colonic interposition postoesophagectomy - PubMed [Internet]. [cited 2024 Apr 1]. Available from: https://pubmed.ncbi.nlm.nih.gov/30544187/

18. Suhardja TS, Norhadi S, Ee E, Hodgkins B. Comparison of the Thunderbeat and Other Energy Devices in Laparoscopic Colorectal Resection: A Single-Center Experience. J Laparoendosc Adv Surg Tech A. 2018 Dec;28(12):1417–21.

19. Hofmann FO, Holch JW, Heinemann V, Ricard I, Reiser MF, Baumann AB, et al. Prognostic value of radiologically enlarged lymph nodes in patients with metastatic colorectal cancer: Subgroup findings of the randomized, open-label FIRE-3/AIO KRK0306 trial. Eur J Radiol. 2018 Mar;100:124–9.

20. Atkin W, Wooldrage K, Shah U, Skinner K, Brown JP, Hamilton W, et al. Is whole-colon investigation by colonoscopy, computerised tomography colonography or barium enema necessary for all patients with colorectal cancer symptoms, and for which patients would flexible sigmoidoscopy suffice? A retrospective cohort study. Health Technol Assess Winch Engl. 2017 Nov;21(66):1–80.

21. Cerdán Santacruz C, Frasson M, Flor-Lorente B, Ramos Rodríguez JL, Trallero Anoro M, Millán Scheiding M, et al. Laparoscopy may decrease morbidity and length of stay after elective colon cancer resection, especially in frail patients: results from an observational real-life study. Surg Endosc. 2017 Dec;31(12):5032–42.

22. Jia H, Pan Y, Guo X, Zhao L, Wang X, Zhang L, et al. Water Exchange Method Significantly Improves Adenoma Detection Rate: A Multicenter, Randomized Controlled Trial. Am J Gastroenterol. 2017 Apr;112(4):568–76.

23. Sasaki T, Ito Y, Ohue M, Kanemitsu Y, Kobatake T, Ito M, et al. Postoperative Chemoradiotherapy After Local Resection for High-Risk T1 to T2 Low Rectal Cancer: Results of a Single-Arm, Multi-Institutional, Phase II Clinical Trial. Dis Colon Rectum. 2017 Sep;60(9):914–21.

24. Ali R, Baracos VE, Sawyer MB, Bianchi L, Roberts S, Assenat E, et al. Lean body mass as an independent determinant of dose-limiting toxicity and neuropathy in patients with colon cancer treated with FOLFOX regimens. Cancer Med. 2016 Apr;5(4):607–16.

25. Pentheroudakis G, Raptou G, Kotoula V, Wirtz RM, Vrettou E, Karavasilis V, et al. Immune response gene expression in colorectal cancer carries distinct prognostic implications according to tissue, stage and site: a prospective retrospective translational study in the context of a hellenic cooperative oncology group randomised trial. PloS One. 2015;10(5):e0124612.

26. Klare P, Ascher S, Hapfelmeier A, Wolf P, Beitz A, Schmid RM, et al. Patient age and duration of colonoscopy are predictors for adenoma detection in both proximal and distal colon. World J Gastroenterol. 2015 Jan 14;21(2):525–32.

27. Steffens CC, Tschechne B, Schardt C, Jacobs G, Valdix AR, Schmidt P, et al. Observational study of adjuvant therapy with capecitabine in colon cancer. Curr Med Res Opin. 2015 Apr;31(4):731–41.

28. Nachiappan S, Askari A, Malietzis G, Giacometti M, White I, Jenkins JT, et al. The impact of anastomotic leak and its treatment on cancer recurrence and survival following elective colorectal cancer resection. World J Surg. 2015 Apr;39(4):1052–8.

29. Bakker N, Cakir H, Doodeman HJ, Houdijk APJ. Eight years of experience with Enhanced Recovery After Surgery in patients with colon cancer: Impact of measures to improve adherence. Surgery. 2015 Jun;157(6):1130–6.

30. Mussetto A, Frazzoni L, Paggi S, Dari S, Laterza L, Radaelli F, et al. Split dosing with a low-volume preparation is not inferior to split dosing with a high-volume preparation for bowel cleansing in patients with a history of colorectal resection: a randomized trial. Endoscopy. 2015 Oct;47(10):917–24.

31. Sahebally SM, Sarwar A, Cooke F. Short-term outcomes following the use of self-expanding metallic stents in acute malignant colonic obstruction--a single centre experience. Int J Surg Lond Engl. 2014 Nov;12(11):1198–202.

32. Marolleau P, Tougeron D, Allignet B, Cohen R, Sefrioui D, Gallet B, et al. Complete pathological response after chemotherapy or immune checkpoint inhibitors in deficient MMR metastatic colorectal cancer: Results of a retrospective multicenter study. Int J Cancer. 2023 Oct 1;153(7):1376–85.

33. Kou F, Li J, Cao Y, Peng Z, Xu T, Shen L, et al. Immune checkpoint inhibitor-induced colitis with endoscopic evaluation in Chinese cancer patients: a single-centre retrospective study. Front Oncol. 2023;13:1285478.

34. Mu C, Chen L. A retrospective evaluation of short-term results from colonic stenting as a bridge to elective surgery versus emergency surgery for malignant colonic obstruction. Sci Rep. 2023 Jan 28;13(1):1600.

35. Hamada Y, Tanaka K, Ikenoyama Y, Horiki N, Tsuboi J, Yamada R, et al. Risk Factors Associated with Painful Colonoscopy and Prolonged Cecal Intubation Time in Female Patients. J Anus Rectum Colon. 2023;7(3):168–75.

36. Samalavicius NE, Dulskas A, Janusonis V, Klimasauskiene V, Eismontas V, Deduchovas O, et al. Robotic colorectal surgery using the Senhance® robotic system: a single center experience. Tech Coloproctology. 2022 Jun;26(6):437–42.

37. Baker S, Monlezun DJ, Wieghard N, Whitlow C, Margolin D. Are the current colonoscopy recommendations for interval surveillance in patients with polyps enough? Machine learning-augmented propensity score cohort analysis of 1840 patients. Surg Endosc. 2022 Feb;36(2):1284–92.

38. Cheng S, Matuguma SE, de Oliveira GHP, Silva GLR, Cheng H, Sánchez-Luna SA, et al. Colonoscopic Ultrasound-Guided Fine-Needle Aspiration Using a Curvilinear Array Transducer: A Single-Center Retrospective Cohort Study. Dis Colon Rectum. 2022 Feb 1;65(2):e80–4.

39. Duong A, Pohl H, Djinbachian R, Deshêtres A, Barkun AN, Marques PN, et al. Evaluation of the polyp-based resect and discard strategy: a retrospective study. Endoscopy. 2022 Feb;54(2):128–35.

40. Ghareeb WM, Wang X, Zhao X, Xie M, Emile SH, Shawki S, et al. The “terminal line”: a novel sign for the identification of distal mesorectum end during TME for rectal cancer. Gastroenterol Rep. 2022;10:goac050.

41. Hrebinko KA, Reitz KM, Mohammed MK, Nassour I, Watson AR, Cunningham KE, et al. Transanal excision with adjuvant therapy for pT1N0 rectal tumors with high-risk features offers equivalent survival to radical resection: A National Cancer Database analysis. J Surg Oncol. 2022 Mar;125(3):475–83.

42. El-Sharkawy F, Gushchin V, Plerhoples TA, Liu C, Emery EL, Collins DT, et al. Minimally invasive surgery for T4 colon cancer is associated with better outcomes compared to open surgery in the National Cancer Database. Eur J Surg Oncol J Eur Soc Surg Oncol Br Assoc Surg Oncol. 2021 Apr;47(4):818–27.

43. Alkrekshi A, Tamaskar I. Safety of Immune Checkpoint Inhibitors in Patients with Cancer and Hepatitis C Virus Infection. The Oncologist. 2021 May;26(5):e827–30.

44. Oki E, Shinto E, Shimokawa M, Yamaguchi S, Ishiguro M, Hasegawa S, et al. Evaluation of a 55-gene classifier as a prognostic biomarker for adjuvant chemotherapy in stage III colon cancer patients. BMC Cancer. 2021 Dec 14;21(1):1332.

45. Halamkova J, Kazda T, Pehalova L, Gonec R, Kozakova S, Bohovicova L, et al. Second primary malignancies in colorectal cancer patients. Sci Rep. 2021 Feb 2;11(1):2759.

46. Renelus BD, Dixit D, Nguyen PT, Njoku KK, Patel PB, Pintor-Jimenez K, et al. Short-term aspirin and statin chemoprophylaxis did not reduce the risk of developing advanced adenomatous polyps in Black patients. BMC Gastroenterol. 2021 Oct 17;21(1):379.

47. Milone M, Degiuli M, Allaix ME, Ammirati CA, Anania G, Barberis A, et al. Mid-transverse colon cancer and extended versus transverse colectomy: Results of the Italian society of surgical oncology colorectal cancer network (SICO CCN) multicenter collaborative study. Eur J Surg Oncol J Eur Soc Surg Oncol Br Assoc Surg Oncol. 2020 Sep;46(9):1683–8.

48. Mao C, Yang X, Zhu C, Xu J, Yu Y, Shen X, et al. Concordance Between Watson for Oncology and Multidisciplinary Teams in Colorectal Cancer: Prognostic Implications and Predicting Concordance. Front Oncol. 2020;10:595565.

49. Roberto M, Arrivi G, Lo Bianco F, Cascinu S, Gelsomino F, Caputo F, et al. Evaluation of Prognostic Factors for Survival in Transverse Colon Cancer. Cancers. 2020 Aug 30;12(9):2457.

50. Crippa J, Duchalais E, Machairas N, Merchea A, Kelley SR, Larson DW. Long-term Oncological Outcomes Following Anastomotic Leak in Rectal Cancer Surgery. Dis Colon Rectum. 2020 Jun;63(6):769–77.

51. Miller E, Nalin A, Diaz Pardo D, Arnett A, Abushahin L, Husain S, et al. Stage I Squamous Cell Carcinoma of the Anus: Is Radiation Therapy Alone Sufficient Treatment? Cancers. 2020 Nov 4;12(11):3248.

52. Shi Y, Song Z, Gu Y, Zhang Y, Zhang T, Zhao R. Short-Term Outcomes of Three-Port Laparoscopic Right Hemicolectomy Versus Five-Port Laparoscopic Right Hemicolectomy: With a Propensity Score Matching Analysis. J Investig Surg Off J Acad Surg Res. 2020 Oct;33(9):822–7.

53. Kawasaki K, Nakamura S, Esaki M, Kurahara K, Eizuka M, Nuki Y, et al. Clinical usefulness of magnifying colonoscopy for the diagnosis of ulcerative colitis-associated neoplasia. Dig Endosc Off J Jpn Gastroenterol Endosc Soc. 2019 Apr;31 Suppl 1:36–42.

54. Numata M, Sawazaki S, Aoyama T, Tamagawa H, Godai T, Sato T, et al. Laparoscopic surgery in patients diagnosed with clinical N2 colon cancer. Surg Today. 2019 Jun;49(6):507–12.

55. David Y, Ottaviano L, Park J, Iqbal S, Likhtshteyn M, Kumar S, et al. Confounders in Adenoma Detection at Initial Screening Colonoscopy: A Factor in the Assessment of Racial Disparities as a Risk for Colon Cancer. J Cancer Ther. 2019 Apr;10(4):269–89.

56. Woff E, Hendlisz A, Ameye L, Garcia C, Kamoun T, Guiot T, et al. Validation of Metabolically Active Tumor Volume and Total Lesion Glycolysis as 18F-FDG PET/CT–derived Prognostic Biomarkers in Chemorefractory Metastatic Colorectal Cancer. J Nucl Med Off Publ Soc Nucl Med. 2019 Feb 1;60(2):178–84.

57. Bhatt A, Bhamre R, Rohila J, Kalikar V, Desouza A, Saklani A. Patients with extensive regional lymph node involvement (pN2) following potentially curative surgery for colorectal cancer are at increased risk for developing peritoneal metastases: a retrospective single-institution study. Colorectal Dis Off J Assoc Coloproctology G B Irel. 2019 Mar;21(3):287–96.

58. Carriles C, Jimenez-Fonseca P, Sánchez-Cánovas M, Pimentel P, Carmona-Bayonas A, García T, et al. Trifluridine/Tipiracil (TAS-102) for refractory metastatic colorectal cancer in clinical practice: a feasible alternative for patients with good performance status. Clin Transl Oncol Off Publ Fed Span Oncol Soc Natl Cancer Inst Mex. 2019 Dec;21(12):1781–5.

59. Pelz JOW, Wagner J, Lichthardt S, Baur J, Kastner C, Matthes N, et al. Laparoscopic right-sided colon resection for colon cancer-has the control group so far been chosen correctly? World J Surg Oncol. 2018 Jun 28;16(1):117.

60. Kang BM, Kim HJ, Kye BH, Lee SC, Lee KY, Park SJ, et al. Multicenter, randomized single-port versus multiport laparoscopic surgery (SIMPLE) trial in colon cancer: an interim analysis. Surg Endosc. 2018 Mar;32(3):1540–9.

61. Dahdaleh FS, Sherman SK, Poli EC, Vigneswaran J, Polite BN, Sharma MR, et al. Obstruction predicts worse long-term outcomes in stage III colon cancer: A secondary analysis of the N0147 trial. Surgery. 2018 Dec;164(6):1223–9.

62. Manzenreiter L, Spaun G, Weitzendorfer M, Luketina R, Antoniou SA, Wundsam H, et al. A proposal for a tailored approach to diverting ostomy for colorectal anastomosis. Minerva Chir. 2018 Feb;73(1):29–35.

63. Sage PY, Trilling B, Waroquet PA, Voirin D, Girard E, Faucheron JL. Laparoscopic delayed coloanal anastomosis without diverting ileostomy for low rectal cancer surgery: 85 consecutive patients from a single institution. Tech Coloproctology. 2018 Jul;22(7):511–8.

64. Fritz CDL, Smith ZL, Elsner J, Hollander T, Early D, Kushnir V. Prolonged Cecal Insertion Time Is Not Associated with Decreased Adenoma Detection When a Longer Withdrawal Time Is Achieved. Dig Dis Sci. 2018 Nov;63(11):3120–5.

65. Kim MK, Kye BH, Lee IK, Oh ST, Ahn CH, Lee YS, et al. Outcome of bridge to surgery stenting for obstructive left colon cancer. ANZ J Surg. 2017 Dec;87(12):E245–50.

66. Burgess NG, Bassan MS, McLeod D, Williams SJ, Byth K, Bourke MJ. Deep mural injury and perforation after colonic endoscopic mucosal resection: a new classification and analysis of risk factors. Gut. 2017 Oct;66(10):1779–89.

67. Pecorelli N, Amodeo S, Frasson M, Vignali A, Zuliani W, Braga M. Ten-year outcomes following laparoscopic colorectal resection: results of a randomized controlled trial. Int J Colorectal Dis. 2016 Jul;31(7):1283–90.

68. Sharara AI, Harb AH, Sarkis FS, Chalhoub JM, Habib RH. Body mass index and quality of bowel preparation: Real life vs. clinical trials. Arab J Gastroenterol Off Publ Pan-Arab Assoc Gastroenterol. 2016 Mar;17(1):11–6.

69. Grade M, Ridwelski K, Voigt I, Ghadimi BM, Mann B. [Robotic Rectal Cancer Surgery]. Zentralbl Chir. 2016 Apr;141(2):165–9.

70. Plumb AA, Pathiraja F, Nickerson C, Wooldrage K, Burling D, Taylor SA, et al. Appearances of screen-detected versus symptomatic colorectal cancers at CT colonography. Eur Radiol. 2016 Dec;26(12):4313–22.

71. Lapeyre-Prost A, Hug de Larauze M, Chibaudel B, Garcia ML, Guering-Meyer V, Bouché O, et al. Feasibility of Capecitabine and Oxaliplatin Combination Chemotherapy Without Central Venous Access Device in Patients With Stage III Colorectal Cancer. Clin Colorectal Cancer. 2016 Sep;15(3):250–6.

72. Dos Anjos DA, Perez RO, Habr-Gama A, São Julião GP, Vailati BB, Fernandez LM, et al. Semiquantitative Volumetry by Sequential PET/CT May Improve Prediction of Complete Response to Neoadjuvant Chemoradiation in Patients With Distal Rectal Cancer. Dis Colon Rectum. 2016 Sep;59(9):805–12.

73. Imai K, Hotta K, Yamaguchi Y, Kakushima N, Tanaka M, Takizawa K, et al. Preoperative indicators of failure of en bloc resection or perforation in colorectal endoscopic submucosal dissection: implications for lesion stratification by technical difficulties during stepwise training. Gastrointest Endosc. 2016 May;83(5):954–62.

74. An MS, Yoo JH, Kim KH, Bae KB, Choi CS, Hwang JW, et al. T4 stage and preoperative anemia as prognostic factors for the patients with colon cancer treated with adjuvant FOLFOX chemotherapy. World J Surg Oncol. 2015 Feb 19;13:64.

75. Jeong IS, Yoo JH, Seo SH, An MS, Kim KH, Bae KB, et al. Association Between Time (Initiation and Length) and Oncological Outcomes for the Patients with Colon Cancer Treated with Adjuvant Chemotherapy. Indian J Surg. 2015 Dec;77(Suppl 3):1252–7.

76. Vogelaar FJ, Abegg R, van der Linden JC, Cornelisse HGJM, van Dorsten FRC, Lemmens VE, et al. Epidural analgesia associated with better survival in colon cancer. Int J Colorectal Dis. 2015 Aug;30(8):1103–7.

77. Mistrangelo M, Allaix ME, Cassoni P, Giraudo G, Arolfo S, Morino M. Laparoscopic versus open resection for transverse colon cancer. Surg Endosc. 2015 Aug;29(8):2196–202.

78. Wang L, Li ZY, Li ZW, Li YH, Sun YS, Ji JF, et al. Efficacy and safety of neoadjuvant intensity-modulated radiotherapy with concurrent capecitabine for locally advanced rectal cancer. Dis Colon Rectum. 2015 Feb;58(2):186–92.

79. Ross WA, Thirumurthi S, Lynch PM, Rashid A, Pande M, Shafi MA, et al. Detection rates of premalignant polyps during screening colonoscopy: time to revise quality standards? Gastrointest Endosc. 2015 Mar;81(3):567–74.

80. Thirunavukarasu P, Talati C, Munjal S, Attwood K, Edge SB, Francescutti V. Effect of Incorporation of Pretreatment Serum Carcinoembryonic Antigen Levels Into AJCC Staging for Colon Cancer on 5-Year Survival. JAMA Surg. 2015 Aug;150(8):747–55.

81. Kim CY, Seo SH, An MS, Kim KH, Bae KB, Hwang JW, et al. ERCC1 as a Predictive Marker for FOLFOX Chemotherapy in an Adjuvant Setting. Ann Coloproctology. 2015 Jun;31(3):92–7.

82. Daniels L, Ünlü Ç, de Wijkerslooth TR, Stockmann HB, Kuipers EJ, Boermeester MA, et al. Yield of colonoscopy after recent CT-proven uncomplicated acute diverticulitis: a comparative cohort study. Surg Endosc. 2015 Sep;29(9):2605–13.

83. Degener S, Pohle A, Strelow H, Mathers MJ, Zumbé J, Roth S, et al. Long-term experience of hyperbaric oxygen therapy for refractory radio- or chemotherapy-induced haemorrhagic cystitis. BMC Urol. 2015 May 8;15:38.

84. Lee KK, Jandorf L, Itzkowitz SH. Diminutive polyps among black and Latino populations undergoing screening colonoscopy: evidence supporting a resect and discard approach. Gastrointest Endosc. 2015 Mar;81(3):728–32.

85. Maeda K, Nagahara H, Shibutani M, Ohtani H, Sakurai K, Toyokawa T, et al. Efficacy of intracorporeal reinforcing sutures for anastomotic leakage after laparoscopic surgery for rectal cancer. Surg Endosc. 2015 Dec;29(12):3535–42.

86. Hugen N, van de Velde CJH, de Wilt JHW, Nagtegaal ID. Metastatic pattern in colorectal cancer is strongly influenced by histological subtype. Ann Oncol Off J Eur Soc Med Oncol. 2014 Mar;25(3):651–7.

87. Maitra RK, Acheson AG, Gornall C, Scholefield JH, Williams JP, Maxwell-Armstrong CA. Results of laparoscopic colorectal surgery from a national training center. Asian J Surg. 2014 Jan;37(1):1–7.

88. Amato B, Esposito G, Serra R, Compagna R, Vigliotti G, Bianco T, et al. One-step mini-invasive treatment of abdominal aortic-iliac aneurysm associated with colo-rectal cancer. Int J Surg Lond Engl. 2014;12 Suppl 2:S193–6.

89. Sammartino P, Sibio S, Biacchi D, Cardi M, Mingazzini P, Rosati MS, et al. Long-term results after proactive management for locoregional control in patients with colonic cancer at high risk of peritoneal metastases. Int J Colorectal Dis. 2014 Sep;29(9):1081–9.
