# Supplementary material for: Sex discrepancies in cancer research: a systematic review of prospective and retrospective investigations in lung, melanoma, and colorectal cancers
Source: Front Glob Womens Health. 2024 Nov 11;5:1445139. doi: 10.3389/fgwh.2024.1445139 (PMC11586390; doi:10.3389/fgwh.2024.1445139)
Supplement: Supplementary file 2 [file Table2.docx]

**Supplemental Table S2A.** Lung Retrospective Studies Included in Systematic Review

| **Author, year** | **Title** | **Male/female** |
| --- | --- | --- |
| (68)Sun et al., 2023 | Optimal therapy for concomitant EGFR and TP53 mutated non-small cell lung cancer: a real-world study | 52/72 |
| (69)Zhu et al., 2023 | Efficacy and safety of camrelizumab combined with albumin-bound paclitaxel as third- or later-line regimen in patients with advanced non-small cell lung cancer | 190/67 |
| (70)Sakamoto et al., 2023 | Biomarker Testing in Patients With Unresectable Advanced or Recurrent Non-Small Cell Lung Cancer | 1013/466 |
| (71)Saw et al., 2023 | PD-L1 score as a prognostic biomarker in asian early-stage epidermal growth factor receptor-mutated lung cancer | 286/169 |
| (72)Chen et al., 2023 | The predictive value of YAP-1 and POU2F3 for the efficacy of immuno-chemotherapy in extensive-stage SCLC patients | 19/2 |
| (73)Brose et al., 2023 | PET/CT reading for relapse in non-small cell lung cancer after chemoradiotherapy in the PET-Plan trial cohort | 62/20 |
| (74)McLaughlin et. al 2023 | Superior sulcus non-small cell lung cancers (Pancoast tumors): Current outcomes after multidisciplinary management | 81/74 |
| (75)Woo et al., 2023 | Predictive scoring of high-grade histology among early-stage lung cancer patients: The MOSS score | 211/289 |
| (76)Hizal et al., 2023 | The percentage of ALK-positive cells and the efficacy of first-line alectinib in advanced non-small cell lung cancer: is it a novel factor for stratification? (Turkish Oncology Group Study) | 109/102 |
| (77)Kayauchi et al., 2023 | Using Compression Therapy to Treat Bilateral Lower Limb Edema in Patients with Lung Cancer: A Case Series | 4/3 |
| (78)Hasegawa et al., 2023 | Impact of the Ablative Margin on Local Tumor Progression after Radiofrequency Ablation for Lung Metastases from Colorectal Carcinoma: Supplementary Analysis of a Phase II Trial (MLCSG-0802) | 49/21 |
| (79)Gallina et al., 2023 | A prognostic score from a multicentric retrospective analysis of patients affected by sarcoma with metachronous lung metastases undergoing metastasectomy | 134/117 |
| (80)Mutsaers et al., 2023 | Stereotactic or Conventional Radiation for Early-Stage Non-small Cell Lung Cancer: A Systematic Review and Meta-Analysis | 97/106 |
| (81)Gilardone et al., 2023 | Osimertinib vs. afatinib as first-line treatment for patients with metastatic non-small cell lung cancer with an EGFR exon 19 deletion or exon 21 L858R mutation | 22/64 |
| (82)Lovoli et al., 2023 | Long-Term Survival and Failure Outcomes of Single-Fraction Stereotactic Body Radiation Therapy in Early Stage NSCLC | 112/151 |
| (83)Kareff et al., 2023 | Efficacy and outcomes of ramucirumab and docetaxel in patients with metastatic non-small cell lung cancer after disease progression on immune checkpoint inhibitor therapy: Results of a monocentric, retrospective analysis | 18/17 |
| (84)Majam et al., 2023 | CYP450 and drug efflux transporters polymorphism influence clinical outcomes of Thai osimertinib-treated non-small cell lung cancer patients | 20/43 |
| (85)Arnon et al., 2023 | Clinical Characteristics, Response to Platinum-Based Chemotherapy and Poly (Adenosine Phosphate-Ribose) Polymerase Inhibitors in Advanced Lung Cancer Patients Harboring BRCA Mutations | 30/22 |
| (86)Knetki-Wróblewska et al., 2023 | Nivolumab or Atezolizumab in the Second-Line Treatment of Advanced Non-Small Cell Lung Cancer? A Prognostic Index Based on Data from Daily Practice | 149/111 |
| (87)Meng et al., 2023 | Enhanced Efficacy of Chemotherapy by Addition of Immune Checkpoint Inhibitors in Stage IV Large Cell Neuroendocrine Carcinoma of the Lung: A Real-World Analysis | 16/8 |
| (88)Lin et al., 2022 | Outcomes of SBRT for lung oligo-recurrence of non-small cell lung cancer: a retrospective analysis | 35/15 |
| (89)Dowlati et al., 2022 | Immune Checkpoint Blockade Outcome in Small-Cell Lung Cancer and Its Relationship With Retinoblastoma Mutation Status and Function | 27/15 |
| (90)Chen et al., 2022 | Prophylactic cranial irradiation (PCI) versus active surveillance in patients with limited-stage small cell lung cancer: a retrospective, multicentre study | 746/322 |
| (91)Yu et al., 2022 | Trends in Postoperative Intensity-Modulated Radiation Therapy Use and Its Association With Survival Among Patients With Incompletely Resected Non-Small Cell Lung Cancer | 2439/2044 |
| (92)Zhong et al., 2022 | PD-1/PD-L1 combined with LAG3 is associated with clinical activity of immune checkpoint inhibitors in metastatic primary pulmonary lymphoepithelioma-like carcinoma | 17/31 |
| (93)Shah et al., 2022 | The Risk of Opportunistic Infections and the Role of Antibiotic Prophylaxis in Patients on Checkpoint Inhibitors Requiring Steroids | 75/37 |
| (94)Maurer et al., 2022 | PET/CT-based adaptive radiotherapy of locally advanced non-small cell lung cancer in multicenter yDEGRO ARO 2017-01 cohort study | 269/74 |
| (95)Humbert et al., 2022 | Prognostic value of immunotherapy-induced organ inflammation assessed on 18FDG PET in patients with metastatic non-small cell lung cancer | 89/48 |
| (96)Hatton et al., 2022 | A study of non small cell lung cancer (NSCLC) patients with brain metastasis: A single centre experience | 224/229 |
| (97)Hattori et al., 2022 | Oncologic outcomes of segmentectomy for stage IA radiological solid-predominant lung cancer >2 cm in maximum tumour size | 100/115 |
| (98)Nieder et al., 2022 | Primary systemic therapy for patients with brain metastases from lung cancer ineligible for targeted agents | 18/20 |
| (99)Kapoor et al., 2022 | Long-Term Outcomes of Crizotinib Treated ALK-Positive Lung Cancer Patients: A Retrospective Audit of Prospective Data from Resource-Constrained Settings | 120/68 |
| (100)Wang et al., 2022 | Safety and Efficacy of Stereotactic Ablative Radiotherapy for Ultra-Central Lung Cancer | 43/15 |
| (101)Hu et al., 2022 | Efficacy and Safety of Gefitinib Plus Anlotinib for Patients with EGFR Positive Advanced Non-Small-Cell Lung Cancer: A Retrospective Exploratory Study | 17/19 |
| (102)Bassi et al., 2022 | Role of radiomics in predicting lung cancer spread through air spaces in a heterogeneous dataset | 85/64 |
| (103)Chen et al., 2022 | The efficacy and safety of immune checkpoint inhibitors combined with chemotherapy or anti-angiogenic therapy as a second-line or later treatment option for advanced non-small cell lung cancer: a retrospective comparative cohort study | 116/29 |
| (104)Zhao et al., 2022 | Comparison of perioperative outcomes among non-small cell lung cancer patients with neoadjuvant immune checkpoint inhibitor plus chemotherapy, EGFR-TKI, and chemotherapy alone: a real-world evidence study | 147/47 |
| (105)Benjamin et al., 2022 | The Role of Chemotherapy Plus Immune Checkpoint Inhibitors in Oncogenic-Driven NSCLC: A University of California Lung Cancer Consortium Retrospective Study | 112/134 |
| (106)Wang et al., 2022 | The efficacy and safety of intrapleural hyperthermic perfusion in patients with malignant pleural effusion undergoing video-assisted thoracic surgery: a single-arm clinical trial | 16/14 |
| (107)Keek et al., 2022 | Investigation of the added value of CT-based radiomics in predicting the development of brain metastases in patients with radically treated stage III NSCLC | 133/86 |
| (108)Hindocha et al., 2022 | Gross tumour volume radiomics for prognostication of recurrence & death following radical radiotherapy for NSCLC | 278/231 |
| (109)Zhong et al., 2022 | Deep Learning for Prediction of N2 Metastasis and Survival for Clinical Stage I Non-Small Cell Lung Cancer | 1470/1193 |
| (110)Abughararah et al., 2021 | Lobe-specific lymph node dissection in stage IA non-small-cell lung cancer: a retrospective cohort study | 544/658 |
| (111)Atkins et al., 2021 | Statin Use, Heart Radiation Dose, and Survival in Locally Advanced Lung Cancer | 380/368 |
| (112)Ke et al., 2021 | Feasibility of semiquantitative 18F-fluorodeoxyglucose PET/computed tomography in patients with advanced lung cancer for interim treatment evaluation of combining immunotherapy and chemotherapy | 35/16 |
| (113)Zalepugas et al., 2021 | Sleeve lobectomy versus lobectomy for primary treatment of non-small-cell lung cancer: A single-center retrospective analysis | 503/255 |
| (114)Zhao et al., 2021 | Utility of comprehensive genomic profiling in directing treatment and improving patient outcomes in advanced non-small cell lung cancer | 696/470 |
| (115)Liu et al., 2021 | Using a risk model for probability of cancer in pulmonary nodules | 235/223 |
| (116)Alkrekshi and Tamaskar, 2021 | Safety of Immune Checkpoint Inhibitors in Patients with Cancer and Hepatitis C Virus Infection | 30/10 |
| (117)Chan et al., 2021 | Outcomes with segmentectomy versus lobectomy in patients with clinical T1cN0M0 non-small cell lung cancer | 176/193 |
| (118)Sehgal et al., 2021 | Association of Extended Dosing Intervals or Delays in Pembrolizumab-based Regimens With Survival Outcomes in Advanced Non-small-cell Lung Cancer | 48/44 |
| (119)Takamori et al., 2021 | Survival benefit from immunocheckpoint inhibitors in stage IV non-small cell lung cancer patients with brain metastases: A National Cancer Database propensity-matched analysis | 22641/19871 |
| (120)Alipour et al., 2021 | Nodal metabolic tumour volume on baseline 18 F-FDG PET/CT and overall survival in stage II and III NSCLC patients undergoing curative-intent chemoradiotherapy/radiotherapy | 55/34 |
| (121)Zauderer et al., 2021 | The use of a next-generation sequencing-derived machine-learning risk-prediction model (OncoCast-MPM) for malignant pleural mesothelioma: a retrospective study | 209/59 |
| (122)Bongiovanni et al., 2021 | Immune Checkpoint Inhibitors With or Without Bone-Targeted Therapy in NSCLC Patients With Bone Metastases and Prognostic Significance of Neutrophil-to-Lymphocyte Ratio | 70/41 |
| (123)Cho et al., 2021 | Intracranial failure after hippocampal-avoidance prophylactic cranial irradiation in limited-stage small-cell lung cancer patients | 93/13 |
| (124)May et al., 2021 | Outcomes for localized treatment of large cell neuroendocrine carcinoma of the lung in the United States | 815/708 |
| (125)Cao et al.., 2021 | Does denosumab offer survival benefits? -Our experience with denosumab in metastatic non-small cell lung cancer patients treated with immune-checkpoint inhibitors | 37/32 |
| (126)Chaurasia et al., 2021 | Early-Stage Non-Small Cell Lung Cancer Stereotactic Body Radiation Therapy (SBRT) Outcomes in an Equal Access Military Setting | 60/45 |
| (127)Chen et al., 2021 | Pembrolizumab Alone or Combined With Chemotherapy in Advanced NSCLC With PD-L1 ≥50%: Results of a Retrospective Study | 182/24 |
| (128)Ding et al., 2021 | Plasma pre-treatment T790M relative allelic frequency in patients with advanced EGFR-mutated non-small cell lung cancer predicts treatment response to subsequent-line osimertinib | 48/91 |
| (129)Jiang et al., 2021 | Efficacy and Safety of Anlotinib Monotherapy as Third-Line Therapy for Elderly Patients with Non-Small Cell Lung Cancer: A Real-World Exploratory Study | 59/24 |
| (130)Hao et al., 2021 | Clinical Activity and Safety of Anlotinib Combined with PD-1 Blockades for Patients with Previously Treated Small Cell Lung Cancer | 26/10 |
| (131)Ksienski et al., 2021 | Prognostic significance of the neutrophil-to-lymphocyte ratio and platelet-to-lymphocyte ratio for advanced non-small cell lung cancer patients with high PD-L1 tumor expression receiving pembrolizumab | 99/121 |
| (132)Chen et al., 2020 | Response and outcomes after anti-CTLA4 versus anti-PD1 combined with stereotactic body radiation therapy for metastatic non-small cell lung cancer: retrospective analysis of two single-institution prospective trials | 22/11 |
| (133)Ahmed et al., 2020 | Performance Status and Age as Predictors of Immunotherapy Outcomes in Advanced Non-Small-Cell Lung Cancer | 159/126 |
| (134)Arias Ron et al., 2020 | Efficacy and safety of Nivolumab in older patients with pretreated lung cancer: A subgroup analysis of the Galician lung cancer group | 36/2 |
| (135)Chen et al., 2021 | Management of Non-Small-Cell Lung Cancer Patients Initially Diagnosed With 1 to 3 Synchronous Brain-Only Metastases: A Retrospective Study | 175/77 |
| (136)Li et al., 2020 | Development and Validation of a Risk Score for Prediction of Venous Thromboembolism in Patients With Lung Cancer | 527/300 |
| (137)Wang et al., 2020 | Retrospective analysis of the effectiveness and tolerability of nab-paclitaxel in Chinese elderly patients with advanced non-small-cell lung carcinoma | 52/24 |
| (138)Jacob et al., 2020 | Lung Cancer Survival in Patients With Autoimmune Disease | 87/309 |
| (139)Handy et al., 2020 | Results of Lung Cancer Screening in the Community | 1248/1265 |
| (140)Correale et al., 2020 | Distinctive germline expression of class I human leukocyte antigen (HLA) alleles and DRB1 heterozygosis predict the outcome of patients with non-small cell lung cancer receiving PD-1/PD-L1 immune checkpoint blockade | 99/20 |
| (141)Cifarelli et al., 2020 | Role of Gamma Knife Radiosurgery in Small Cell Lung Cancer: A Multi-Institutional Retrospective Study of the International Radiosurgery Research Foundation (IRRF) | 144/142 |
| (142)Friedes et al., 2020 | Consolidative Radiotherapy in Oligometastatic Lung Cancer: Patient Selection With a Prediction Nomogram | 42/49 |
| (143)Geier et al., 2020 | Duration of nivolumab for pretreated, advanced non-small-cell lung cancer | 187/72 |
| (144)Haentschel et al., 2020 | Cryobiopsy increases the EGFR detection rate in non-small cell lung cancer | 260/154 |
| (145)Shrimali et al., 2020 | Setting up a lung stereotactic body radiotherapy service in a tertiary center in Eastern India: The process, quality assurance, and early experience | 13/2 |
| (146)Park et al., 2020 | Association of the prognostic model iSEND with PD-1/L1 monotherapy outcome in non-small-cell lung cancer | 492/386 |
| (147)Yang et al., 2020 | Superior efficacy of immunotherapy-based combinations over monotherapy for EGFR-mutant non-small cell lung cancer acquired resistance to EGFR-TKIs | 12/19 |
| (148)Friedlaender et al., 2020 | Impact of performance status on non-small-cell lung cancer patients with a PD-L1 tumour proportion score ≥50% treated with front-line pembrolizumab | 196/106 |
| (149)Remon et al., 2020 | Outcomes in oncogenic-addicted advanced NSCLC patients with actionable mutations identified by liquid biopsy genomic profiling using a tagged amplicon-based NGS assay | 25/56 |
| (150)Al-Toubah | Capecitabine and Temozolomide in Advanced Lung Neuroendocrine Neoplasms | 10/10 |
| (151)Zhou et al., 2020 | Prognostic Value of Pretreatment Albumin-to-Alkaline Phosphatase Ratio in Extensive-Disease Small-Cell Lung Cancer: A Retrospective Cohort Study | 197/27 |
| (152)Xu et al., 2020 | Pretreatment neutrophil-to-lymphocyte ratio is a predictive biomarker for EGFR TKI-treated patients with advanced EGFR-mutant Non-small cell lung cancer | 31/35 |
| (153)Su et al., 2020 | Procedure-specific prognostic impact of micropapillary subtype may guide resection strategy in small-sized lung adenocarcinomas: a multicenter study | 375/397 |
| (154)Buttigliero et al., 2019 | Retrospective Assessment of a Serum Proteomic Test in a Phase III Study Comparing Erlotinib plus Placebo with Erlotinib plus Tivantinib (MARQUEE) in Previously Treated Patients with Advanced Non-Small Cell Lung Cancer | 591/405 |
| (155)Pavan et al., 2019 | Peripheral Blood Markers Identify Risk of Immune‐Related Toxicity in Advanced Non‐Small Cell Lung Cancer Treated with Immune‐Checkpoint Inhibitors | 125/59 |
| (156) Bjørnhart et al., 2019 | Efficacy and safety of immune checkpoint inhibitors in a Danish real life non-small cell lung cancer population: a retrospective cohort study | 44/74 |
| (157)Hersberger et al., 2019 | Quantitative Imaging Assessment for Clinical Trials in Oncology | 23/26 |
| (158)Zeng et al., 2019 | Association of Twice-Daily Radiotherapy With Subsequent Brain Metastases in Adults With Small Cell Lung Cancer | 574/2014 |
| (159)Weckler et al., 2019 | Survival following Multimodality Treatment Including Surgery for Stage IA-IIIB Small-Cell Lung Cancer | 29/18 |
| (160)Kyang et al., 2019 | Long-term survival outcomes of cytoreductive surgery and perioperative intraperitoneal chemotherapy: Single-institutional experience with 1225 cases | 538/687 |
| (161)Tubin et al., 2019 | Mono-institutional phase 2 study of innovative Stereotactic Body RadioTherapy targeting PArtial Tumor HYpoxic (SBRT-PATHY) clonogenic cells in unresectable bulky non-small cell lung cancer: profound non-targeted effects by sparing peri-tumoral immune microenvironment | 39/21 |
| (162)Abbas et al., 2019 | Survival and late toxicities following concurrent chemo-radiotherapy for locally advanced stage III non-small cell lung cancer: findings of a 10-year Australian single centre experience with long term clinical follow up | 41/22 |
| (163)Digesu et al., 2018 | Long-term outcomes after near-infrared sentinel lymph node mapping in non-small cell lung cancer | 10/32 |
| (164)Touati et al., 2018 | European Organisation for Research and Treatment of Cancer Soft Tissue and Bone Sarcoma Group Experience with Advanced/Metastatic Epithelioid Sarcoma Patients Treated in Prospective Trials: Clinical Profile and Response to Systemic Therapy | 17/10 |
| (165)Huang et al., 2018 | Perioperative Management May Improve Long-term Survival in Patients After Lung Cancer Surgery: A Retrospective Cohort Study | 374/214 |
| (166)Mallow et al., 2019 | Safety and diagnostic performance of pulmonologists performing electromagnetic guided percutaneous lung biopsy (SPiNperc) | 64/65 |
| (167)Vanhove et al., 2018 | The plasma glutamate concentration as a complementary tool to differentiate benign PET-positive lung lesions from lung cancer | 439/286 |
| (168)Brandt et al., 2018 | Factors associated with distant recurrence following R0 lobectomy for pN0 lung adenocarcinoma | 334/559 |
| (169)Caivano et al., 2018 | Re-irradiation in lung disease by SBRT: a retrospective, single institutional study | 15/7 |
| (170)Cornwell et al., 2018 | Video-assisted thoracoscopic lobectomy is associated with greater recurrence-free survival than stereotactic body radiotherapy for clinical stage I lung cancer | 172/11 |
| (171)Ma et al., 2018 | [The Initial Experience of Video-assisted Thoracic Surgery Segmentectomy for Early Stage Lung Cancer] | 11/24 |
| (172)Endoh et al., 2018 | Hyperbaric oxygen therapy for postoperative ischemic bronchitis after resection of lung cancer | 7/0 |
| (173)Li et al., 2018 | Prognostic factors of oligometastatic non-small cell lung cancer: a meta-analysis | 309/258 |
| (174)Jiang et al., 2018 | A retrospective study of shrinking field radiation therapy during chemoradiotherapy in stage III non-small cell lung cancer | 91/6 |
| (175)Shang et al., 2018 | Local ablative therapy with or without chemotherapy for non-small-cell lung cancer patients with postoperative oligometastases | 128/35 |
| (176)Imai et al., 2017 | A retrospective study of amrubicin monotherapy for the treatment of relapsed small cell lung cancer in elderly patients | 76/10 |
| (177)Wang et al., 2017 | Heart dosimetric analysis of three types of cardiac toxicity in patients treated on dose-escalation trials for Stage III non-small-cell lung cancer | 61/51 |
| (178)Karube et al., 2017 | Carbon-ion radiotherapy for patients with advanced stage non-small-cell lung cancer at multicenters | 49/15 |
| (179)Vinogradskiy et al., 2017 | Assessing the use of 4DCT-ventilation in pre-operative surgical lung cancer evaluation | 8/8 |
| (180)Baron et al., 2017 | Clinical Utility of Chromosomal Aneusomy in Individuals at High Risk of Lung Cancer | 612/226 |
| (181)Yang et al., 2017 | Polymorphisms in BMP2/BMP4, with estimates of mean lung dose, predict radiation pneumonitis among patients receiving definitive radiotherapy for non-small cell lung cancer | 362/201 |
| (182)Gautschi et al., 2017 | Targeting RET in Patients With RET-Rearranged Lung Cancers: Results From the Global, Multicenter RET Registry | 79/86 |
| (183)Bagley et al., 2017 | Pretreatment neutrophil-to-lymphocyte ratio as a marker of outcomes in nivolumab-treated patients with advanced non-small-cell lung cancer | 80/95 |
| (184)Peretti et al., 2016 | ALK gene copy number gains in non-small-cell lung cancer: prognostic impact and clinico-pathological correlations | 79/49 |
| (185)Salama et al., 2016 | Positive Interaction between Prophylactic Cranial Irradiation and Maintenance Sunitinib for Untreated Extensive-Stage Small Cell Lung Cancer Patients After Standard Chemotherapy: A Secondary Analysis of CALGB 30504 (ALLIANCE) | 38/47 |
| (186)Wang et al., 2016 | A propensity-matched analysis of surgery and stereotactic body radiotherapy for early stage non-small cell lung cancer in the elderly | 163/17 |
| (187)Zhai et al., 2016 | Impact of age on adjuvant chemotherapy after radical resection in patients with non-small cell lung cancer | 561/304 |
| (188)Reichert et al., 2016 | A standardized technique of systematic mediastinal lymph node dissection by video-assisted thoracoscopic surgery (VATS) leads to a high rate of nodal upstaging in early-stage non-small cell lung cancer | 62/15 |
| (189)Kodama et al., 2016 | Oncologic Outcomes of Segmentectomy Versus Lobectomy for Clinical T1a N0 M0 Non-Small Cell Lung Cancer | 157/155 |
| (190)Feng et al., 2016 | Prognostic value of tumor-infiltrating lymphocytes for patients with completely resected stage IIIA(N2) non-small cell lung cancer | 198/122 |
| (191)Billiet et al., 2016 | Patterns of Locoregional Relapses in Patients with Contemporarily Staged Stage III-N2 NSCLC Treated with Induction Chemotherapy and Resection: Implications for Postoperative Radiotherapy Target Volumes | 109/41 |
| (192)Ko et al., 2015 | Predictive value of 18F-FDG PET and CT morphologic features for recurrence in pathological stage IA non-small cell lung cancer | 48/97 |
| (193)Winther-Larsen et al., 2015 | Evaluation of factors associated with loco-regional failure and survival in limited disease small cell lung cancer patients treated with chemoradiotherapy | 69/78 |
| (194)Mazières et al., 2015 | Crizotinib therapy for advanced lung adenocarcinoma and a ROS1 rearrangement: results from the EUROS1 cohort | 11/21 |
| (195)Feng et al., 2015 | The emerging outcome of postoperative radiotherapy for stage IIIA(N2) non-small cell lung cancer patients: based on the three-dimensional conformal radiotherapy technique and institutional standard clinical target volume | 35/35 |
| (196)Davis et al., 2015 | Stereotactic body radiotherapy for centrally located early-stage non-small cell lung cancer or lung metastases from the RSSearch(®) patient registry | 59/52 |
| (197)Buti et al., 2015 | Predictive role of erythrocyte macrocytosis during treatment with pemetrexed in advanced non-small cell lung cancer patients | 115/76 |
| (198)Lo et al., 2015 | Delay of treatment change after objective progression on first-line erlotinib in epidermal growth factor receptor-mutant lung cancer | 20/72 |
| (199)Cho et al., 2015 | Modified one-day etoposide and cisplatin combination for previously untreated extensive-disease small-cell lung cancer: A retrospective evaluation of 36 cases | 25/11 |
| (200)Fabre et al., 2015 | Evolution of induction chemotherapy for non-small cell lung cancer over the last 30 years: A surgical appraisal | 591/141 |
| (201)Liu et al., 2015 | Video-assisted thoracoscopic surgery and thoracotomy during lobectomy for clinical stage I non-small-cell lung cancer have equivalent oncological outcomes: A single-center experience of 212 consecutive resections | 125/87 |
| (202)Liu et al., 2015 | Simultaneous integrated dose reduction intensity-modulated radiotherapy applied to an elective nodal area of limited-stage small-cell lung cancer | 35/17 |
| (203)Corrales-Rodriguez et al., 2014 | Mutations in NSCLC and their link with lung cancer-associated thrombosis: a case-control study | 80/79 |
| (204)Ryan et al., 2014 | A combined prognostic serum interleukin-8 and interleukin-6 classifier for stage 1 lung cancer in the prostate, lung, colorectal, and ovarian cancer screening trial | 370/178 |
| (205)Landreneau | Recurrence and survival outcomes after anatomic segmentectomy versus lobectomy for clinical stage I non-small-cell lung cancer: a propensity-matched analysis | 283/341 |
| (206)Liu et al., 2014 | Application of ThinPrep bronchial brushing cytology in the early diagnosis of lung cancer: a retrospective study | 381/214 |
| (207)Ji et al., 2014 | Simultaneous integrated boost intensity-modulated radiotherapy for treatment of locally advanced non-small-cell lung cancer: a retrospective clinical study | 37/11 |
| (208)Yang et al., 2014 | Surgical treatment of metachronous second primary lung cancer | 106/37 |
| (209)Askoxylakis | Trimodal therapy for stage III-N2 non-small-cell lung carcinoma: a single center retrospective analysis | 48/23 |
| (210)Altinbas | The effect of small-molecular-weight heparin added to chemotherapy on survival in small-cell lung cancer - A retrospective analysis | 60/7 |
| (211)Inal et al., 2014 | Is there any significance of lung cancer histology to compare the diagnostic accuracies of (18)F-FDG-PET/CT and (99m)Tc-MDP BS for the detection of bone metastases in advanced NSCLC? | 47/6 |
| (212)Takeshita et al., 2014 | Weekly administration of paclitaxel and carboplatin with concurrent thoracic radiation in previously untreated elderly patients with locally advanced non-small-cell lung cancer: A case series of 20 patients | 17/3 |
| (213)Kim et al., 2014 | Outcome of active anti-cancer treatment in elderly patients with advanced non-small cell lung cancer: A single center experience | 100/44 |

**Supplemental Table S2B.** Lung Prospective Studies Included in Systematic Review

| **Author, year** | **Title** | **Male/female** |
| --- | --- | --- |
| (1)Zhang et al., 2023 | Selective Mediastinal Lymph Node Dissection Strategy for Clinical T1N0 Invasive Lung Cancer: A Prospective, Multicenter, Clinical Trial | 281/439 |
| (2)Chung et al., 2023 | Prediction Models for Mediastinal Metastasis and Its Detection by Endobronchial Ultrasound-Guided Transbronchial Needle Aspiration in Potentially Operable Non-Small Cell Lung Cancer: A Prospective Study | 577/321 |
| (3)Bodensohn et al., 2023 | Stereotactic radiosurgery versus whole-brain radiotherapy in patients with 4-10 brain metastases: A nonrandomized controlled trial | 58/52 |
| (4)Takeda et al., 2023 | Correlation of toxicities and efficacies of pemetrexed with clinical factors and single-nucleotide polymorphisms: a prospective observational study | 49/22 |
| (5)Li et al., 2023 | Low-dose versus standard-dose computed tomography-guided biopsy for pulmonary nodules: a randomized controlled trial | 130/70 |
| (6)Citak et al., 2023 | N1 lymph node detection in lymph node harvesting in non-small cell lung cancer: Formaldehyde exposure is a drawback? | 153/17 |
| (7)Derosa et al., 2022 | Intestinal Akkermansia muciniphila predicts clinical response to PD-1 blockade in advanced non-small cell lung cancer patients | 226/112 |
| (8)Kim et al., 2022 | Blood-based tumor mutational burden as a biomarker for atezolizumab in non-small cell lung cancer: the phase 2 B-F1RST trial | 149/122 |
| (9)Stamatis et al., 2022 | Survival outcomes in a prospective randomized multicenter Phase III trial comparing patients undergoing anatomical segmentectomy versus standard lobectomy for non-small cell lung cancer up to 2 cm | 32/21 |
| (10)Tammemagi et al., 2022 | USPSTF2013 versus PLCOm2012 lung cancer screening eligibility criteria (International Lung Screening Trial): interim analysis of a prospective cohort study | 2494/2046 |
| (11)Gadgeel et al., 2022 | Comparison of SP142 and 22C3 Immunohistochemistry PD-L1 Assays for Clinical Efficacy of Atezolizumab in Non-Small Cell Lung Cancer: Results From the Randomized OAK Trial | 758/467 |
| (12)Recuerdo-Diaz et al., 2022 | Treatment and intention-to-treat propensity score analysis to evaluate the impact of video-assisted thoracic surgery on 90-day mortality after anatomical resection for lung cancer | 1885/836 |
| (12)Wang et al., 2022 | Beyond diabetes mellitus: role of metformin in non-muscle-invasive bladder cancer | 101/21 |
| (13)Zhang et al., 2022 | Dose-escalation by hypofractionated simultaneous integrated boost IMRT in unresectable stage III non-small-cell lung cancer | 19/6 |
| (14)Wurstbauer et al., 2022 | Locally advanced NSCLC: a plea for sparing the ipsilateral normal lung-prospective, clinical trial with DART-bid (dose-differentiated accelerated radiation therapy, 1.8 Gy twice daily) by VMAT | 67/33 |
| (15)Takahashi et al., 2022 | Real-world effectiveness of nivolumab in advanced gastric cancer: the DELIVER trial (JACCRO GC-08) | 347/140 |
| (16)Tanzawa et al., 2022 | Prospective analysis of factors precluding the initiation of durvalumab from an interim analysis of a phase II trial of S-1 and cisplatin with concurrent thoracic radiotherapy followed by durvalumab for unresectable, locally advanced non-small cell lung cancer in Japan (SAMURAI study) | 51/8 |
| (17)Jongen et al., 2022 | Clinical Outcomes After Proton Beam Therapy for Locally Advanced Non-Small Cell Lung Cancer: Analysis of a Multi-institutional Prospective Registry | 104/90 |
| (18)Wass et al., 2022 | Durvalumab after Sequential High Dose Chemoradiotherapy versus Standard of Care (SoC) for Stage III NSCLC: A Bi-Centric Trospective Comparison Focusing on Pulmonary Toxicity | 48/30 |
| (19)Ester et al., 2021 | Feasibility of a multimodal exercise, nutrition, and palliative care intervention in advanced lung cancer | 4/6 |
| (20)Chen et al., 2021 | Multiomics Analysis Reveals Distinct Immunogenomic Features of Lung Cancer with Ground-Glass Opacity | 49/52 |
| (21)Quaife et al., 2021 | Psychological Targets for Lung Cancer Screening Uptake: A Prospective Longitudinal Cohort Study | 4248/3438 |
| (22)Chau et al., 2021 | Prospective correlation between the patient microbiome with response to and development of immune-mediated adverse effects to immunotherapy in lung cancer | 18/16 |
| (23)Gargiulo et al., 2021 | Chemotherapy-induced neutropenia and treatment efficacy in advanced non-small-cell lung cancer: a pooled analysis of 6 randomized trials | 1172/357 |
| (24)Debieuvre et al., 2021 | Two-year survival with nivolumab in previously treated advanced non-small-cell lung cancer: A real-world pooled analysis of patients from France, Germany, and Canada | 1616/910 |
| (25)Martin et al., 2021 | Prospective Evaluation of the First Integrated Positron Emission Tomography/Dual-Energy Computed Tomography System in Patients With Lung Cancer | 14/7 |
| (26)Yeap et al., 2021 | Mesothelioma Risk Score: A New Prognostic Pretreatment, Clinical-Molecular Algorithm for Malignant Pleural Mesothelioma | 312/72 |
| (27)Weir-McCall et al., 2021 | Impact of solitary pulmonary nodule size on qualitative and quantitative assessment using 18F-fluorodeoxyglucose PET/CT: the SPUTNIK trial | 184/171 |
| (28)Tsim et al., 2021 | Serum Proteomics and Plasma Fibulin-3 in Differentiation of Mesothelioma From Asbestos-Exposed Controls and Patients With Other Pleural Diseases | 316/51 |
| (29)Carles et al., 2021 | FDG-PET Radiomics for Response Monitoring in Non-Small-Cell Lung Cancer Treated with Radiation Therapy | 30/18 |
| (30)Durm et al., 2020 | A Phase 2 Trial of Consolidation Pembrolizumab Following Concurrent Chemoradiation for Patients With Unresectable Stage III Non–Small Cell Lung Cancer: Hoosier Cancer Research Network LUN 14–179 | 59/33 |
| (31)Jabbour et al., 2020 | Phase 1 Trial of Pembrolizumab Administered Concurrently With Chemoradiotherapy for Locally Advanced Non–Small Cell Lung Cancer | 10/11 |
| (32)Lin et al., 2020 | Phase II Trial of Concurrent Atezolizumab With Chemoradiation for Unresectable NSCLC | 27/13 |
| (33)Welsh et al., 2020 | Pembrolizumab with or without radiation therapy for metastatic non-small cell lung cancer: a randomized phase I/II trial | 26/14 |
| (34)Ross et al., 2020 | AFT-16: Phase II trial of atezolizumab before and after definitive chemoradiation (CRT) for unresectable stage III non-small cell lung cancer (NSCLC). | 30/32 |
| (35)Donington et al., 2020 | Resection following concurrent chemotherapy and high-dose radiation for stage IIIA non-small cell lung cancer | 106/80 |
| (36)Li et al., 2020 | Gustave Roussy Immune Score based on a three-category risk assessment scale serves as a novel and effective prognostic indicator for surgically resectable early-stage non-small-cell lung cancer: A propensity score matching retrospective cohort study | 256/49 |
| (37)Wu et al., 2020 | Evaluation of a National Comprehensive Cancer Network Guidelines-Based Decision Support Tool in Patients With Non-Small Cell Lung Cancer: A Nonrandomized Clinical Trial | 135/98 |
| (38)Criscitiello et al., 2020 | Pretreatment Blood Parameters Predict Efficacy from Immunotherapy Agents in Early Phase Clinical Trials | 62/91 |
| (39)Dohm et al., 2020 | Identification of CD37, cystatin A, and IL-23A gene expression in association with brain metastasis: analysis of a prospective trial | 60/42 |
| (40)Akamatsu et al., 2020 | Immune-Related Adverse Events by Immune Checkpoint Inhibitors Significantly Predict Durable Efficacy Even in Responders with Advanced Non-Small Cell Lung Cancer | 18/5 |
| (41)Tsuchiya et al., 2020 | A single-arm, phase 2 study of adjuvant chemotherapy with oral tegafur-uracil for pathologically lymphovascular invasion positive stage IA non-small cell lung cancer: LOGIK0602 study | 39/13 |
| (42)Hurkmans et al., 2020 | Tumor mutational load, CD8+ T cells, expression of PD-L1 and HLA class I to guide immunotherapy decisions in NSCLC patients | 18/12 |
| (43)Owonikoko et al., 2020 | Randomized Phase II Study of Paclitaxel plus Alisertib versus Paclitaxel plus Placebo as Second-Line Therapy for SCLC: Primary and Correlative Biomarker Analyses | 101/77 |
| (44)Peters et al., 2019 | Safety evaluation of nivolumab added concurrently to radiotherapy in a standard first line chemo-radiotherapy regimen in stage III non-small cell lung cancer-The ETOP NICOLAS trial | 51/26 |
| (45)Ahn et al., 2019 | Phase II, prospective single-arm study of adjuvant pembrolizumab in N2 positive non-small cell lung cancer (NSCLC) treated with neoadjuvant concurrent chemoradiotherapy followed by curative resection: Preliminary results. | 23/14 |
| (46)Theelen et al., 2019 | Effect of Pembrolizumab After Stereotactic Body Radiotherapy vs Pembrolizumab Alone on Tumor Response in Patients With Advanced Non–Small Cell Lung Cancer | 20/15 |
| (47)Parl et al., 2019 | Sequencing of therapy following first-line afatinib in patients with EGFR mutation-positive non-small cell lung cancer | 146/260 |
| (48)Sequist et al., 2019 | Randomized Phase II Trial of Seribantumab in Combination with Erlotinib in Patients with EGFR Wild-Type Non-Small Cell Lung Cancer | 77/52 |
| (49)Dziadziusko et al., 2019 | Afatinib in NSCLC With HER2 Mutations: Results of the Prospective, Open-Label Phase II NICHE Trial of European Thoracic Oncology Platform (ETOP) | 4/9 |
| (50)Arrieta et al., 2019 | Effect of Metformin Plus Tyrosine Kinase Inhibitors Compared With Tyrosine Kinase Inhibitors Alone in Patients With Epidermal Growth Factor Receptor-Mutated Lung Adenocarcinoma: A Phase 2 Randomized Clinical Trial | 48/91 |
| (51)Gambazzi et al., 2019 | Image analysis in posttreatment non-small cell lung cancer surveillance: specialists' interpretations reviewed by the thoracic multidisciplinary tumor board | 62/27 |
| (52)Antonia et al., 2018 | Overall Survival with Durvalumab after Chemoradiotherapy in Stage III NSCLC | 500/213 |
| (53)Sagawa et al., 2018 | A prospective 5-year follow-up study after limited resection for lung cancer with ground-glass opacity | 25/28 |
| (54)Tibaldi et al., 2018 | Cytidine deaminase enzymatic activity is a prognostic biomarker in gemcitabine/platinum-treated advanced non-small-cell lung cancer: a prospective validation study | 94/27 |
| (55)Saji et al., 2018 | A proposal for a comprehensive risk scoring system for predicting postoperative complications in octogenarian patients with medically operable lung cancer: JACS1303 | 540/355 |
| (56)Ning et al., 2017 | Incidence and Predictors of Pericardial Effusion After Chemoradiation Therapy for Locally Advanced Non-Small Cell Lung Cancer | 288/214 |
| (57)Jamal-Hanjani et al., 2017 | Tracking the Evolution of Non-Small-Cell Lung Cancer | 62/38 |
| (58)Teraoka et al., 2017 | Early Immune-Related Adverse Events and Association with Outcome in Advanced Non-Small Cell Lung Cancer Patients Treated with Nivolumab: A Prospective Cohort Study | 27/16 |
| (59)Hata et al., 2017 | Adherence and feasibility of 2 treatment schedules of S-1 as adjuvant chemotherapy for patients with completely resected advanced lung cancer: a multicenter randomized controlled trial | 51/27 |
| (60)Boyer et al., 2016 | Toxicity of definitive and post-operative radiation following ipilimumab in non-small cell lung cancer | 9/7 |
| (61)Decaluwé et al., 2016 | Central tumour location should be considered when comparing N1 upstaging between thoracoscopic and open surgery for clinical stage I non-small-cell lung cancer | 226/108 |
| (62)Remon et al., 2016 | Sunitinib in patients with advanced thymic malignancies: Cohort from the French RYTHMIC network | 19/9 |
| (63)Saiag et al., 2016 | Prospective assessment of a gene signature potentially predictive of clinical benefit in metastatic melanoma patients following MAGE-A3 immunotherapeutic (PREDICT) | 65/56 |
| (64)Safi et al., 2015 | Sublobar Resection, Radiofrequency Ablation or Radiotherapy in Stage I Non-Small Cell Lung Cancer | 79/37 |
| (65)Endo et al., 2014 | A prospective study of surgical procedures for patients with oligometastatic non-small cell lung cancer | 4/1 |
| (66)Sozzi et al., 2014 | Clinical utility of a plasma-based miRNA signature classifier within computed tomography lung cancer screening: a correlative MILD trial study | 607/332 |
| (67)Wang et al., 2014 | Circulating endothelial-derived activated microparticle: a useful biomarker for predicting one-year mortality in patients with advanced non-small cell lung cancer | 61/46 |
