# Supplementary material for: Sex discrepancies in cancer research: a systematic review of prospective and retrospective investigations in lung, melanoma, and colorectal cancers
Source: Front Glob Womens Health. 2024 Nov 11;5:1445139. doi: 10.3389/fgwh.2024.1445139 (PMC11586390; doi:10.3389/fgwh.2024.1445139)
Supplement: Supplementary file 3 [file Table3.docx]

**Supplemental Table S3A.** Melanoma Retrospective Studies Included in Systematic Review

| **Author, year** | **Title** | **Males/Females** |
| --- | --- | --- |
| Zhong et al., 2023 (1) | Comparative analysis of adjuvant therapy for stage III BRAF-mut melanoma: A real-world retrospective study from single center in China | 40/53 |
| Piñero-Madrona et al., 2023 (2) | Lower limb cutaneous melanoma surgery: location matters | 160/61 |
| Van Not et al., 2023 (3) | Response to checkpoint inhibition and targeted therapy in melanoma patients with concurrent haematological malignancies | 2796/1842 |
| Schumann et al., 2023 (4) | Real-world outcomes using PD-1 antibodies and BRAF + MEK inhibitors for adjuvant melanoma treatment from 39 skin cancer centers in Germany, Austria and Switzerland | 653/462 |
| Bhave et al., 2023 (5) | Efficacy and toxicity of adjuvant radiotherapy in recurrent melanoma after adjuvant immunotherapy | 42/29 |
| Koch et al., 2023 (6) | Liver-directed treatment is associated with improved survival and increased response to immune checkpoint blockade in metastatic uveal melanoma: results from a retrospective multicenter trial | 91/91 |
| Zhong et al., 2023 (7) | Survival impact of immediate complete lymph node dissection for Chinese acral and cutaneous melanoma with micrometastasis in sentinel nodes: a retrospective study | 67/63 |
| Medri et al., 2023 (8) | A retrospective observational study on cutaneous adverse events induced by immune checkpoint inhibitors | 189/116 |
| Bai et al., 2023 (9) | Dabrafenib plus trametinib versus anti-PD-1 monotherapy as adjuvant therapy in *BRAF* V600-mutant stage III melanoma after definitive surgery: a multicenter, retrospective cohort study | 326/272 |
| Bravo et al., 2023 (10) | HEV-associated dendritic cells are observed in metastatic tumor-draining lymph nodes of cutaneous melanoma patients with longer distant metastasis-free survival after adjuvant immunotherapy | 17/12 |
| Cui et al., 2023 (11) | Safety and efficacy of Pucotenlimab (HX008) - a humanized immunoglobulin G4 monoclonal antibody in patients with locally advanced or metastatic melanoma: a single-arm, multicenter, phase II study | 51/68 |
| Hedderson et al., 2023 (12) | Rates of malignancies among patients with moderate to severe atopic dermatitis: a retrospective cohort study | 2507/4542 |
| Ayati et al., 2023 (13) | Predictive value and accuracy of [^18^F]FDG PET/CT modified response criteria for checkpoint immunotherapy in patients with advanced melanoma | 55/36 |
| Quildrian et al., 2023 (14) | Current management of patients with cutaneous melanoma with a positive sentinel lymph node | 18/13 |
| Zhang et al., 2023 (15) | Melanin-targeted [^18^F]-PFPN PET imaging for prognosticating patients with melanoma | 47/29 |
| Kähler et al., 2023 (16) | Preferences of German and Swiss melanoma patients for toxicities versus melanoma recurrence during adjuvant treatment (GERMELATOX-A-trial) | 74/86 |
| Broman et al., 2023 (17) | International Center-Level Variation in Utilization of Completion Lymph Node Dissection and Adjuvant Systemic Therapy for Sentinel Lymph Node-Positive Melanoma at Major Referral Centers | 672/437 |
| Brown et al., 2023 (18) | Five-year survival and clinical correlates among patients with advanced non-small cell lung cancer, melanoma and renal cell carcinoma treated with immune check-point inhibitors in Australian tertiary oncology centres | 137/65 |
| Afrăsânie et al., 2023 (19) | Real Check RIO: A Real-World Analysis of Nivolumab in First Line Metastatic Melanoma Assessing Efficacy, Safety and Predictive Factors | 32/19 |
| Augustin et al., 2023 (20) | Metformin is associated with improved clinical outcomes in patients with melanoma: a retrospective, multi-institutional study | 455/212 |
| Nardin et al., 2023 (21) | Efficacy of Immune Checkpoint Inhibitor (ICI) Rechallenge in Advanced Melanoma Patients' Responders to a First Course of ICI: A Multicenter National Retrospective Study of the French Group of Skin Cancers (Groupe de Cancérologie Cutanée, GCC) | 47/38 |
| Kalantari et al., 2023 (22) | Prognostic Value of Baseline ^18^F-FDG PET/CT to Predict Brain Metastasis Development in Melanoma Patients | 87/72 |
| Tomsitz et al., 2023 (23) | Tebentafusp in Patients with Metastatic Uveal Melanoma: A Real-Life Retrospective Multicenter Study | 39/39 |
| Dimitriou et al., 2022 (24) | Single-agent anti-PD-1 or combined with ipilimumab in patients with mucosal melanoma: an international, retrospective, cohort study | 187/358 |
| Bhave et al., 2022 (25) | Efficacy of anti-PD-1 and ipilimumab alone or in combination in acral melanoma | 171/154 |
| Serra-Bellver et al., 2022 (26) | Real-world outcomes with ipilimumab and nivolumab in advanced melanoma: a multicentre retrospective study | 401/296 |
| Jones et al., 2022 (27) | Retrospective review of outcomes associated with metastatic melanoma patients treated with 1st-line BRAF-targeted therapy | 12/13 |
| Salzmann et al., 2022 (28) | MEK inhibitors for pre-treated, NRAS-mutated metastatic melanoma: A multi-centre, retrospective study | 19/14 |
| Salaün et al., 2022 (29) | Nivolumab plus ipilimumab in metastatic uveal melanoma: a real-life, retrospective cohort of 47 patients | 24/23 |
| Kuzmanovszki et al., 2022 (30) | Anti-PD-1 Monotherapy in Advanced Melanoma-Real-World Data from a 77-Month-Long Retrospective Observational Study | 68/51 |
| Pandya et al., 2022 (31) | Can we reduce excision margins for head and neck melanoma? A 12-year retrospective study | 194/111 |
| Erglu et al., 2022 (32) | Outcomes with adjuvant anti-PD-1 therapy in patients with sentinel lymph node-positive melanoma without completion lymph node dissection | 245/141 |
| Williams et al., 2022 (33) | Melanoma sentinel lymph node biopsy and completion lymph node dissection: A regional hospital experience | 85/72 |
| Broman et al., 2022  (34) | Evidence and implementation gaps in management of sentinel node-positive melanoma in the United States | 6079/4161 |
| Montgomery et al., 2022 (35) | Real-World Adherence to Nodal Surveillance for Sentinel Lymph Node-Positive Melanoma | 61/48 |
| Hyeok Kim et al., 2022 (36) | Two-team-approached free flap reconstruction for plantar malignant melanoma: An observational (STROBE-compliant) trial | 16/5 |
| Googe et al., 2022 (37) | Theragnostic significance of tumor-infiltrating lymphocytes and Ki67 in BRAFV600-mutant metastatic melanoma (BRIM-3 trial) | 200/153 |
| Homan et al., 2022 (38) | Treatment related toxicities with combination BRAF and MEK inhibitor therapy in resected stage III melanoma | 11/9 |
| Tropea et al., 2022 (39) | The role of sentinel node tumor burden in modeling the prognosis of melanoma patients with positive sentinel node biopsy: an Italian melanoma intergroup study (N = 2,086) | 1203/883 |
| Tolstrup et al., 2022 (40) | Impact of patient-reported outcomes on symptom monitoring during treatment with checkpoint inhibitors: health-related quality of life among melanoma patients in a randomized controlled trial | 78/68 |
| Placzke et al., 2022 (41) | The Analysis of Trends in Survival for Patients with Melanoma Brain Metastases with Introduction of Novel Therapeutic Options before the Era of Combined Immunotherapy-Multicenter Italian-Polish Report | 516/373 |
| Lalonde et al., 2022 (42) | Improved Uveal Melanoma Copy Number Subtypes Including an Ultra-High-Risk Group | 440/481 |
| Darabi et al,. 2022 (43) | Transcriptional Profiling of Malignant Melanoma Reveals Novel and Potentially Targetable Gene Fusions | 777/478 |
| Lechner et al., 2022 (44) | International Multicenter Study of Clinical Outcomes of Sinonasal Melanoma Shows Survival Benefit for Patients Treated with Immune Checkpoint Inhibitors and Potential Improvements to the Current TNM Staging System | 233/271 |
| Qian et al., 2021 (45) | Effect of immunotherapy time-of-day infusion on overall survival among patients with advanced melanoma in the USA (MEMOIR): a propensity score-matched analysis of a single-centre, longitudinal study | 197/102 |
| Formozo et al., 2021 (46) | Retrospective Analysis of Rechallenge with Ipilimumab in Patients with Metastatic Melanoma | 10/12 |
| Rose et al., 2021 (47) | Biologic subtypes of melanoma predict survival benefit of combination anti-PD1+anti-CTLA4 immune checkpoint inhibitors versus anti-PD1 monotherapy | 145/104 |
| Versluis et al., 2021 (48) | Neoadjuvant ipilimumab plus nivolumab in synchronous clinical stage III melanoma | 6/1 |
| Aglietta et al., 2021 (49) | Retrospective Chart Review of Dabrafenib Plus Trametinib in Patients with Metastatic BRAF V600-Mutant Melanoma Treated in the Individual Patient Program (DESCRIBE Italy) | 269/230 |
| Phillips et al., 2021 (50) | A Contemporary Report of Clinical Outcomes in Patients with Melanoma Brain Metastases | 185/91 |
| Yu et al., 2021 (51) | Survival of advanced melanoma patients treated with immunotherapy and targeted therapy: A real-world study | 337/166 |
| Crosby et al., 2021 (52) | Associations of Physical Activity and Exercise with Health-related Outcomes in Patients with Melanoma During and After Treatment: A Systematic Review | 3/11 |
| Mejbel et al., 2021 (53) | Prognostic significance of acral lentiginous histologic type in T1 melanoma | 397/431 |
| Monestier et al., 2021 (54) | Effectiveness and safety of nivolumab in patients with advanced melanoma: A multicenter, observational study | 245/155 |
| Ogata et al., 2021 (55) | The efficacy of anti-programmed cell death protein 1 therapy among patients with metastatic acral and metastatic mucosal melanoma | 45/52 |
| Gibney et al., 2021 (56) | PET/CT scan and biopsy-driven approach for safe anti-PD-1 therapy discontinuation in patients with advanced melanoma | 34/18 |
| Broman et al., 2021 (57) | Surveillance of Sentinel Node-Positive Melanoma Patients Who Receive Adjuvant Therapy Without Undergoing Completion Lymph Node Dissection | 110/67 |
| Ra, et al., 2021 (58) | Factors associated with immune checkpoint inhibitor use among older adults with late-stage melanoma: A population-based study | 2902/1617 |
| Jiang et al., 2021 (59) | Single institutional outcomes of whole brain radiotherapy for metastatic melanoma brain metastases | 39/24 |
| Madonna et al., 2021 (60) | Clinical Categorization Algorithm (CLICAL) and Machine Learning Approach (SRF-CLICAL) to Predict Clinical Benefit to Immunotherapy in Metastatic Melanoma Patients: Real-World Evidence from the Istituto Nazionale Tumori IRCCS Fondazione Pascale, Napoli, Italy | 320/258 |
| Matsui et al., 2021 (61) | Observation policy for sentinel node metastasis of melanoma: Comparative study with completion lymph node dissection in Japanese patients | 33/26 |
| Ressler et al., 2021 (62) | Real-life use of talimogene laherparepvec (T-VEC) in melanoma patients in centers in Austria, Switzerland and Germany | 44/44 |
| Pescarmona et al., 2021 (63) | Evaluation of TTV replication as a biomarker of immune checkpoint inhibitors efficacy in melanoma patients | 27/16 |
| El Sharouni et al., 2021 (64) | High discordance rate in assessing sentinel node positivity in cutaneous melanoma: Expert review may reduce unjustified adjuvant treatment | 175/147 |
| Di Guardo et al., 2021 (65) | Liquid Biopsy and Radiological Response Predict Outcomes Following Discontinuation of Targeted Therapy in Patients with BRAF Mutated Melanoma | 12/12 |
| Moore et al., 2021 (66) | Automated digital TIL analysis (ADTA) adds prognostic value to standard assessment of depth and ulceration in primary melanoma | 56/24 |
| Bouchereau et al., 2021 (67) | Impact of prior treatment with immune checkpoint inhibitors on dacarbazine efficacy in metastatic melanoma | 53/19 |
| Mangin et al., 2021 (68) | Decreased survival in patients treated by chemotherapy after targeted therapy compared to immunotherapy in metastatic melanoma | 51/37 |
| Flaus et al., 2021 (69) | FDG PET biomarkers for prediction of survival in metastatic melanoma prior to anti-PD1 immunotherapy | 27/29 |
| Kowlblinger et al., 2021 (70) | Adjuvant anti-PD-1 antibody treatment in stage III/IV melanoma: real-world experience and health economic considerations | 55/45 |
| Zanoni et al., 2021 (71) | Use of Ultrasmall Core-Shell Fluorescent Silica Nanoparticles for Image-Guided Sentinel Lymph Node Biopsy in Head and Neck Melanoma: A Nonrandomized Clinical Trial | 18/6 |
| Tian et al., 2021 (72) | Safety Profile of Immunotherapy Combined With Antiangiogenic Therapy in Patients With Melanoma: Analysis of Three Clinical Studies | 33/39 |
| Tejera-Vaquerizo et al., 2021 (73) | Sentinel Lymph Node Biopsy vs. Observation in Thin Melanoma: A Multicenter Propensity Score Matching Study | 2155/2894 |
| Kelly et al., 2021 (74) | Development of a Metastatic Uveal Melanoma Prognostic Score (MUMPS) for Use in Patients Receiving Immune Checkpoint Inhibitors | 35/40 |
| Ksienski et al., 2021 (75) | Time to Treatment With Nivolumab or Pembrolizumab for Patients With Advanced Melanoma in Everyday Practice | 186/116 |
| Lopez-Obregon et al., 2021 (76) | Evaluation of Intra-Lesional Interleukin 2 for the Treatment of In-Transit Melanoma Disease: L'évaluation de l'interleukine-2 intralésionnelle pour traiter les mélanomes en transit | 20/29 |
| Orlova et al., 2021 (77) | Real-World Experience with Targeted Therapy in BRAF Mutant Advanced Melanoma Patients: Results from a Multicenter Retrospective Observational Study Advanced Melanoma in Russia (Experience) (ADMIRE) | 171/211 |
| Kleemann et al., 2021 (78) | Real-World Experience of Talimogene Laherparepvec (T-VEC) in Old and Oldest-Old Patients with Melanoma: A Retrospective Single Center Study | 6/6 |
| Wu et al., 2021 (79) | Survival Benefits of Anti-PD-1 Therapy in Combination With Radiotherapy in Chinese Melanoma Patients With Brain Metastasis | 12/13 |
| Valentin et al., 2021 (80) | Real-World Survival in Patients with Metastatic Melanoma after Discontinuation of Anti-PD-1 Immunotherapy for Objective Response or Adverse Effects: A Retrospective Study | 42/23 |
| Hribernik et al., 2020 (81) | Retrospective analysis of treatment-naive Slovenian patients with metastatic melanoma treated with pembrolizumab - real-world experience | 84/54 |
| Brown et al., 2021 (82) | Combination anti-PD1 and ipilimumab therapy in patients with advanced melanoma and pre-existing autoimmune disorders | 26/29 |
| Ridolfi et al., 2020 (83) | Anti-PD1 antibodies in patients aged ≥ 75 years with metastatic melanoma: A retrospective multicentre study | 103/71 |
| Berger et al., 2020 (84) | Left ventricular ejection fraction decrease related to BRAF and/or MEK inhibitors in metastatic melanoma patients: A retrospective analysis | 51/37 |
| Ziegler et al., 2020 (85) | Toxicity of combined targeted therapy and concurrent radiotherapy in metastatic melanoma patients: a single-center retrospective analysis | 23/9 |
| Mandel et al., 2020 (86) | Long-term vemurafenib therapy in advanced melanoma patients: cutaneous toxicity and prognostic implications | 42/20 |
| Spillane et al., 2020 (87) | Organ Dysfunction in Patients with Advanced Melanoma Treated with Immune Checkpoint Inhibitors | 1654/753 |
| Comito et al., 2020 (88) | Oligoprogression After Checkpoint Inhibition in Metastatic Melanoma Treated With Locoregional Therapy: A Single-center Retrospective Analysis | 22/14 |
| Suo et al., 2020 (89) | Anti-PD1-Induced Immune-Related Adverse Events and Survival Outcomes in Advanced Melanoma | 109/77 |
| Nakamura et al., 2020 (90) | Anti-PD1 checkpoint inhibitor therapy in acral melanoma: a multicenter study of 193 Japanese patients | 114/79 |
| Balatoni et al., 2020 (91) | Biomarkers Associated with Clinical Outcome of Advanced Melanoma Patients Treated with Ipilimumab | 27/20 |
| Boada et al., 2020 (92) | Factors associated with sentinel lymph node status and prognostic role of completion lymph node dissection for thick melanoma | 360/288 |
| Aiken et al., 2020 (93) | Sentinel lymph node biopsy is associated with increased cost in higher risk thin melanoma | 33/37 |
| Betof Warner et al., 2020 (94) | Long-Term Outcomes and Responses to Retreatment in Patients With Melanoma Treated With PD-1 Blockade | 255/141 |
| Uppal et al., 2020 (95) | Regional Node Basin Recurrence in Melanoma Patients: More Common After Node Dissection for Macroscopic Rather than Clinically Occult Nodal Disease | 393/184 |
| Warburton et al., 2020 (96) | Circulating Tumour DNA in Advanced Melanoma Patients Ceasing PD1 Inhibition in the Absence of Disease Progression | 49/21 |
| Rauwerdink et al., 2020 (97) | Adjuvant Therapy Failure Patterns in the Modern Era of Melanoma Management | 50/50 |
| Turiello et al., 2020 (98) | Serum CD73 is a prognostic factor in patients with metastatic melanoma and is associated with response to anti-PD-1 therapy | 324/222 |
| Atkinson et al., 2020 (99) | Dabrafenib plus trametinib is effective in the treatment of BRAF V600-mutated metastatic melanoma patients: analysis of patients from the dabrafenib plus trametinib Named Patient Program (DESCRIBE II) | 150/121 |
| Verver et al., 2020 (100) | The EORTC-DeCOG nomogram adequately predicts outcomes of patients with sentinel node-positive melanoma without the need for completion lymph node dissection | 569/509 |
| Boada et al., 2020 (101) | Age as a prognostic factor in thick and ultrathick melanomas without lymph node metastasis | 232/130 |
| Rajeshuni et al., 2020 (102) | Evaluation of Racial, Ethnic, and Socioeconomic Associations With Treatment and Survival in Uveal Melanoma, 2004-2014 | 2315/2160 |
| Eisendle et al., 2020 (103) | Combining chemotherapy and autologous peptide-pulsed dendritic cells provides survival benefit in stage IV melanoma patients | 99/110 |
| Amaral et al., 2020 (104) | Combined immunotherapy with nivolumab and ipilimumab with and without local therapy in patients with melanoma brain metastasis: a DeCOG* study in 380 patients | 240/140 |
| Takahashi et al., 2020 (105) | Real-world efficacy and safety data of nivolumab and ipilimumab combination therapy in Japanese patients with advanced melanoma | 28/29 |
| Cybulska-Stopa et al., 2020 (106) | Efficacy of ipilimumab after anti-PD-1 therapy in sequential treatment of metastatic melanoma patients - Real world evidence | 61/55 |
| Yamazaki et al., 2020 (107) | Assessment of SPECT-CT fusion images and semi-quantitative evaluation using SUV in 123I-IMP SPECT in patients with choroidal melanoma | 14/11 |
| Peng et al., 2020 (108) | Combining texture features of whole slide images improves prognostic prediction of recurrence-free survival for cutaneous melanoma patients | 92/60 |
| Huang et al., 2020 (109) | Completion lymph node dissection in patients with sentinel lymph node positive cutaneous head and neck melanoma | 397/133 |
| Iravani et al., 2020 (110) | FDG PET/CT for tumoral and systemic immune response monitoring of advanced melanoma during first-line combination ipilimumab and nivolumab treatment | 20/11 |
| Mason et al., 2020 (111) | Combined ipilimumab and nivolumab first-line and after BRAF-targeted therapy in advanced melanoma | 104/48 |
| Shi et al., 2020 (112) | Clinical characteristics of malignant melanoma in central China and predictors of metastasis | 74/93 |
| Huang et al., 2020 (113) | Comparative Analysis of Acral Melanoma in Chinese and Caucasian Patients | 187/155 |
| Jang et al., 2020 (114) | Real-World Recurrence Rates and Economic Burden in Patients with Resected Early-Stage Melanoma | 827/489 |
| Feigelson et al., 2019 (115) | Melanoma incidence, recurrence, and mortality in an integrated healthcare system: A retrospective cohort study | 1116/815 |
| Schilling et al., 2019 (116) | First-line therapy-stratified survival in BRAF-mutant melanoma: a retrospective multicenter analysis | 184/117 |
| Holmberg et al., 2019 (117) | Surgery for gastrointestinal metastases of malignant melanoma - a retrospective exploratory study | 12/3 |
| Márquez-Rodas et al., 2019 (118) | A retrospective chart review study describing metastatic melanoma patients profile and treatment patterns in Spain | 158/126 |
| Liu et al., 2019 (119) | Real-world experience with pembrolizumab in patients with advanced melanoma: A large retrospective observational study | 353/179 |
| Mohr et al., 2019 (120) | Real-World Use of Talimogene Laherparepvec in German Patients with Stage IIIB to IVM1a Melanoma: A Retrospective Chart Review and Physician Survey | 13/14 |
| Mowery et al., 2019 (121) | Retrospective analysis of safety and efficacy of anti-PD-1 therapy and radiation therapy in advanced melanoma: A bi-institutional study | 86/65 |
| Czirbesz et al., 2019 (122) | Efficacy of Vemurafenib Treatment in 43 Metastatic Melanoma Patients with BRAF Mutation. Single-Institute Retrospective Analysis, Early Real-Life Survival Data | 21/22 |
| Jørgensen et al., 2019 (123) | Prophylactic incisional negative pressure wound therapy shows promising results in prevention of wound complications following inguinal lymph node dissection for Melanoma: A retrospective case-control series | 18/23 |
| Cowey et al., 2019 (124) | Real-world treatment patterns and clinical outcomes among patients with advanced melanoma: A retrospective, community oncology-based cohort study (A STROBE-compliant article) | 323/161 |
| Verver et al., 2019 (125) | Upregulation of intratumoral HLA class I and peritumoral Mx1 in ulcerated melanomas | 84/88 |
| Liu et al., 2019 (126) | GDF11 upregulation independently predicts shorter overall-survival of uveal melanoma | 45/35 |
| Gartrell et al., 2019 (127) | Validation of Melanoma Immune Profile (MIP), a Prognostic Immune Gene Prediction Score for Stage II-III Melanoma | 59/19 |
| Ferguson et al., 2019 (128) | Predictors of survival in metastatic melanoma patients with leptomeningeal disease (LMD) | 111/67 |
| Cimminiello et al., 2019 (129) | Pembrolizumab in the treatment of advanced/metastatic melanoma: a single-center institution experience | 29/13 |
| Song et al., 2019 (130) | Survival Outcomes of Patients with Clinical Stage III Melanoma in the Era of Novel Systemic Therapies | 2449/1271 |
| Arheden et al., 2019 (131) | Real-world data on PD-1 inhibitor therapy in metastatic melanoma | 62/54 |
| Polkowska et al., 2019 (132) | Efficacy and safety of BRAF inhibitors and anti-CTLA4 antibody in melanoma patients-real-world data | 667/503 |
| Amaral et al., 2019 (133) | Immunotherapy plus surgery/radiosurgery is associated with favorable survival in patients with melanoma brain metastasis | 93/70 |
| Nan Tie et al., 2019 (134) | The Prognosis and Natural History of In-Transit Melanoma Metastases at a High-Volume Centre | 57/52 |
| Mahvi et al., 2019 (135) | Utility of Level III Axillary Node Dissection in Melanoma Patients with Palpable Axillary Lymph Node Disease | 66/19 |
| Parakh et al., 2019 (136) | Real-world efficacy and toxicity of combined nivolumab and ipilimumab in patients with metastatic melanoma | 30/15 |
| Martin-Algarra et al., 2019 (137) | Effectiveness of dabrafenib in the treatment of patients with BRAF V600-mutated metastatic melanoma in a Named Patient Program | 205/126 |
| Kulkarni et al., 2019 (138) | Deep Learning Based on Standard H&E Images of Primary Melanoma Tumors Identifies Patients at Risk for Visceral Recurrence and Death | 73/34 |
| Hamid et al., 2019 (139) | Safety, Clinical Activity, and Biological Correlates of Response in Patients with Metastatic Melanoma: Results from a Phase I Trial of Atezolizumab | 30/15 |
| Masoud et al., 2019 (140) | Efficacy of Talimogene Laherparepvec (T-VEC) Therapy in Patients with In-Transit Melanoma Metastasis Decreases with Increasing Lesion Size | 12/15 |
| Louveau et al., 2019 (141) | Baseline Genomic Features in BRAFV600-Mutated Metastatic Melanoma Patients Treated with BRAF Inhibitor + MEK Inhibitor in Routine Care | 13/14 |
| Castanares-Zapatero et al., 2019 (142) | Survival of patients with unfavorable prognosis cutaneous melanoma with increased use of immunotherapy agents: a population-based study in Belgium | 211/169 |
| McQuade et al., 2018 (143) | Association of body-mass index and outcomes in patients with metastatic melanoma treated with targeted therapy, immunotherapy, or chemotherapy: a retrospective, multicohort analysis | 347/252 |
| Lattanzi et al., 2018 (144) | Adjuvant NY-ESO-1 vaccine immunotherapy in high-risk resected melanoma: a retrospective cohort analysis | 13/9 |
| Ma et al., 2018 (145) | Autoimmune comorbidities in patients with metastatic melanoma: a retrospective analysis of us claims data | 7574/4454 |
| Sam et al., 2018 (146) | Generalizability of clinical trials of advanced melanoma in the real-world, population-based setting | 173/117 |
| De Caluwé et al., 2018 (147) | Dose-response in choroidal melanoma | 66/69 |
| Tucker et al., 2018 (148) | Risks of Melanoma and Other Cancers in Melanoma-Prone Families over 4 Decades | 410/457 |
| Rachidi et al., 2018 (149) | Postdiagnosis aspirin use and overall survival in patients with melanoma | 798/724 |
| Nakamura et al., 2018 (150) | Use of immune checkpoint inhibitors prolonged overall survival in a Japanese population of advanced malignant melanoma patients: Retrospective single institutional study | 25/20 |
| Okada et al., 2018 (151) | Association Between Immune-Related Adverse Events and Clinical Efficacy in Patients with Melanoma Treated With Nivolumab: A Multicenter Retrospective Study | 4/9 |
| Cowey et al., 2018 (152) | Pembrolizumab Utilization and Outcomes for Advanced Melanoma in US Community Oncology Practices | 110/58 |
| Hauschild et al., 2018 (153) | Modeled Prognostic Subgroups for Survival and Treatment Outcomes in BRAF V600-Mutated Metastatic Melanoma: Pooled Analysis of 4 Randomized Clinical Trials | 783/582 |
| Glitza et al., 2018 (154) | Retrospective review of metastatic melanoma patients with leptomeningeal disease treated with intrathecal interleukin-2 | 32/11 |
| Ibrahim et al., 2018 (155) | Older melanoma patients aged 75 and above retain responsiveness to anti-PD1 therapy: results of a retrospective single-institution cohort study | 53/46 |
| Kähler er al., 2018 (156) | Ipilimumab in metastatic melanoma patients with pre-existing autoimmune disorders | 18/23 |
| Haag et al., 2018 (157) | Phase II trial of ipilimumab in melanoma patients with preexisting humoural immune response to NY-ESO-1 | 20/5 |
| Perier-Muzet et al., 2018 (158) | Association of Immunotherapy With Overall Survival in Elderly Patients With Melanoma | 12/26 |
| Rosner et al., 2018 (159) | Peripheral blood clinical laboratory variables associated with outcomes following combination nivolumab and ipilimumab immunotherapy in melanoma | 124/85 |
| Hanna et al., 2018 (160) | A Population-based Study of Survival Impact of New Targeted and Immune-based Therapies for Metastatic or Unresectable Melanoma | 1733/1060 |
| Afzal et al., 2018 (161) | Efficacy of metformin in combination with immune checkpoint inhibitors (anti-PD-1/anti-CTLA-4) in metastatic malignant melanoma | 34/21 |
| Lee et al., 2018 (162) | Circulating tumor DNA predicts survival in patients with resected high-risk stage II/III melanoma | 77/84 |
| Lo et al., 2018 (163) | Long-Term Survival of Patients with Thin (T1) Cutaneous Melanomas: A Breslow Thickness Cut Point of 0.8 mm Separates Higher-Risk and Lower-Risk Tumors | 3663/2600 |
| Verver et al., 2018 (164) | Development and validation of a nomogram to predict recurrence and melanoma-specific mortality in patients with negative sentinel lymph nodes | 1510/1668 |
| Rossi et al., 2018 (165) | Prediction of Non-sentinel Node Status in Patients with Melanoma and Positive Sentinel Node Biopsy: An Italian Melanoma Intergroup (IMI) Study | 686/534 |
| Sinnamon et al., 2018 (166) | Prediction of Residual Nodal Disease at Completion Dissection Following Positive Sentinel Lymph Node Biopsy for Melanoma | 112/80 |
| Teterycz et al., 2018 (167) | High baseline neutrophil-to-lymphocyte ratio predicts worse outcome in patients with metastatic BRAF-positive melanoma treated with BRAF and MEK inhibitors | 110/105 |
| Dillman et al., 2018 (168) | Randomized phase II trial of autologous dendritic cell vaccines versus autologous tumor cell vaccines in metastatic melanoma: 5-year follow up and additional analyses | 27/15 |
| Kirchberger et al., 2018 (169) | Real world experience in low-dose ipilimumab in combination with PD-1 blockade in advanced melanoma patients | 24/9 |
| Mavor et al., 2018 (170) | Disparities in diagnosis of advanced melanoma: a population-based cohort study | 4287/3755 |
| Lu et al., 2018 (171) | Elevated Levels of BRAFV600 Mutant Circulating Tumor DNA and Circulating Hepatocyte Growth Factor Are Associated With Poor Prognosis in Patients With Metastatic Melanoma | 381/294 |
| Castaneda et al., 2017 (172) | Tumor infiltrating lymphocytes in acral lentiginous melanoma: a study of a large cohort of cases from Latin America | 415/409 |
| Samlowski et al., 2017 (173) | High frequency of brain metastases after adjuvant therapy for high-risk melanoma | 45/14 |
| Fujisawa et al., 2017 (174) | Fluctuations in routine blood count might signal severe immune-related adverse events in melanoma patients treated with nivolumab | 63/38 |
| Long et al., 2017 (175) | Nivolumab for Patients With Advanced Melanoma Treated Beyond Progression: Analysis of 2 Phase 3 Clinical Trials | 323/203 |
| Patel et al., 2017 (176) | Ipilimumab and Stereotactic Radiosurgery Versus Stereotactic Radiosurgery Alone for Newly Diagnosed Melanoma Brain Metastases | 41/13 |
| Zimmer et al., 2017 (177) | Ipilimumab alone or in combination with nivolumab after progression on anti-PD-1 therapy in advanced melanoma | 50/34 |
| Meerveld-Eggink et al., 2017 (178) | Short-term CTLA-4 blockade directly followed by PD-1 blockade in advanced melanoma patients: a single-center experience | 22/18 |
| Weber et al., 2017 (179) | Safety Profile of Nivolumab Monotherapy: A Pooled Analysis of Patients With Advanced Melanoma | 349/227 |
| Leontovich et al., 2017 (180) | Effect of the lymphocyte-to-monocyte ratio on the clinical outcome of chemotherapy administration in advanced melanoma patients | 14/10 |
| Smit et al., 2017 (181) | A Pilot Randomized Controlled Trial of the Feasibility, Acceptability, and Impact of Giving Information on Personalized Genomic Risk of Melanoma to the Public | 59/59 |
| Timerman et al., 2017 (182) | Vitamin D deficiency is associated with a worse prognosis in metastatic melanoma | 144/108 |
| O’Shea et al., 2017 (183) | Which symptoms are linked to a delayed presentation among melanoma patients? A retrospective study | 66/75 |
| Mosquera et al., 2017 (184) | Population-Based Analysis of Completion Lymphadenectomy in Intermediate-Thickness Melanoma | 1329/843 |
| Andtbacka et al., 2016 (185) | Cutaneous head and neck melanoma in OPTiM, a randomized phase 3 trial of talimogene laherparepvec versus granulocyte-macrophage colony-stimulating factor for the treatment of unresected stage IIIB/IIIC/IV melanoma | 68/19 |
| Algazi et al., 2016 (186) | Clinical outcomes in metastatic uveal melanoma treated with PD-1 and PD-L1 antibodies | 32/24 |
| Koskivuo et al., 2016 (187) | Whole body PET/CT in the follow-up of asymptomatic patients with stage IIB-IIIB cutaneous melanoma | 65/45 |
| Srisuttiyakorn et al., 2016 (188) | Intratumoral multinucleated giant cells are not a prognostic pathologic feature in cutaneous melanoma | 316/246 |
| Walter et al., 2016 (189) | Prognostic Implications of Tumor Diameter in Association With Gene Expression Profile for Uveal Melanoma | 164/175 |
| Bol et al., 2015 (190) | Favorable overall survival in stage III melanoma patients after adjuvant dendritic cell vaccination | 46/32 |
| Meani et al., 2015 (191) | The Victorian Melanoma Service: A 20-year review of an Australian multidisciplinary cancer service | 3495/3226 |
| Grignol et al., 2015 (192) | Increased visceral to subcutaneous fat ratio is associated with decreased overall survival in patients with metastatic melanoma receiving anti-angiogenic therapy | 27/15 |
| Ramirez et al., 2015 (193) | Defining the effects of age and gender on immune response and outcomes to melanoma vaccination: a retrospective analysis of a single-institution clinical trials' experience | 224/103 |
| Coupland et al., 2015 (194) | Concordant chromosome 3 results in paired choroidal melanoma biopsies and subsequent tumour resection specimens | 19/9 |
| Smith et al., 2015 (195) | Predicting Overall Survival in Patients With Metastatic Melanoma on Antiangiogenic Therapy and RECIST Stable Disease on Initial Posttherapy Images Using CT Texture Analysis | 25/17 |
| Larkin et al., 2015 (196) | Efficacy and Safety of Nivolumab in Patients With BRAF V600 Mutant and BRAF Wild-Type Advanced Melanoma: A Pooled Analysis of 4 Clinical Trials | 279/161 |
| Harding et al., 2015 (197) | A Retrospective Evaluation of Vemurafenib as Treatment for BRAF-Mutant Melanoma Brain Metastases | 15/12 |
| Takiar et al., 2014 (198) | A choice of radionuclide: Comparative outcomes and toxicity of ruthenium-106 and iodine-125 in the definitive treatment of uveal melanoma | 32/35 |
| Lee et al., 2014 (199) | The impact of socioeconomic status on melanoma clinical trial participation: an observational cohort study from Australia | 5126/3948 |
| Lee et al., 2014 (200) | Features and management of pyrexia with combined dabrafenib and trametinib in metastatic melanoma | 20/12 |
| Choe et al., 2014 (201) | Ocular toxicity in BRAF mutant cutaneous melanoma patients treated with vemurafenib | 12/11 |
| T van der Ploeg et al., 2014 (202) | Melanoma patients with an unknown primary tumor site have a better outcome than those with a known primary following therapeutic lymph node dissection for macroscopic (clinically palpable) nodal disease | 389/162 |
| Egger et al., 2014 (203) | Addition of an iliac/obturator lymph node dissection does not improve nodal recurrence or survival in melanoma | 54/80 |
| Kan et al., 2014 (204) | Podoplanin expression in cancer-associated fibroblasts predicts aggressive behavior in melanoma | 21/34 |
| Bedikian et al., 2014 (205) | Dacarbazine with or without oblimersen (a Bcl-2 antisense oligonucleotide) in chemotherapy-naive patients with advanced melanoma and low-normal serum lactate dehydrogenase: 'The AGENDA trial' | 195/119 |
| Gupta et al., 2014 (206) | DOC-MEK: a double-blind randomized phase II trial of docetaxel with or without selumetinib in wild-type BRAF advanced melanoma | 58/25 |
| Egger et al., 2014 (207) | Comparison of sentinel lymph node micrometastatic tumor burden measurements in melanoma | 92/65 |
| Lau et al., 2014 (208) | A single-centre experience of patients with metastatic melanoma enrolled in a dabrafenib named patient programme | 19/12 |

**Supplemental Table S3B.** Melanoma Prospective Studies Included in Systematic Review

| **Author, year** | **Title** | **Males/Females** |  |
| --- | --- | --- | --- |
| Gómez-Olivas et al., 2023 (209) | Role of Sleep Apnea and Long-Term CPAP Treatment in the Prognosis of Patients With Melanoma: A Prospective Multicenter Study of 443 Patients | 191/200 |  |
| Atkins et al., 2023 (210) | Combination Dabrafenib and Trametinib Versus Combination Nivolumab and Ipilimumab for Patients With Advanced BRAF-Mutant Melanoma: The DREAMseq Trial-ECOG-ACRIN EA6134 | 167/98 |  |
| Ascierto et al., 2023 (211) | Sequencing of Ipilimumab Plus Nivolumab and Encorafenib Plus Binimetinib for Untreated BRAF-Mutated Metastatic Melanoma (SECOMBIT): A Randomized, Three-Arm, Open-Label Phase II Trial | 118/91 |  |
| Carpenter et al., 2023 (212) | Prospective, randomized, double-blind phase 2B trial of the TLPO and TLPLDC vaccines to prevent recurrence of resected stage III/IV melanoma: a prespecified 36-month analysis | 125/62 |  |
| Marchetti et al., 2023 (213) | Prospective validation of dermoscopy-based open-source artificial intelligence for melanoma diagnosis (PROVE-AI study) | 200/235 |  |
| Berking et al., 2023 (214) | COMBI-r: A Prospective, Non-Interventional Study of Dabrafenib Plus Trametinib in Unselected Patients with Unresectable or Metastatic BRAF V600-Mutant Melanoma | 262/210 |  |
| Oldan et al., 2023 (215) | Increased tryptophan, but not increased glucose metabolism, predict resistance of pembrolizumab in stage III/IV melanoma | 20/6 |  |
| Brunsgaard et al., 2023 (216) | Feasibility of personalized circulating tumor DNA detection in stage II and III melanoma | 14/14 |  |
| Meyer et al., 2023 (217) | Identification of high-risk patients with a seven-biomarker prognostic signature for adjuvant treatment trial recruitment in American Joint Committee on Cancer v8 stage I-IIA cutaneous melanoma | 226/213 |  |
| Bartula et al., 2023 (218) | Longitudinal trajectory of quality of life for patients with melanoma brain metastases: A secondary analysis from a whole brain radiotherapy randomized clinical trial | 96/43 |  |
| Cerminara et al., 2023 (219) | Diagnostic performance of augmented intelligence with 2D and 3D total body photography and convolutional neural networks in a high-risk population for melanoma under real-world conditions: A new era of skin cancer screening? | 74/69 |  |
| Gente et al., 2023 (220) | Sex and anti-inflammatory treatment affect outcome of melanoma and non-small cell lung cancer patients with rheumatic immune-related adverse events | 24/26 |  |
| Holmber et al., 2023 (221) | Prognostic Significance of Sentinel Lymph Node Status in Thick Primary Melanomas (> 4 mm) | 5668/4823 |  |
| Pavoine et al., 2023 (222) | Clinical application of a population-based input function (PBIF) for a shortened dynamic whole-body FDG-PET/CT protocol in patients with metastatic melanoma treated by immunotherapy | 20/17 |  |
| Del Vecchio et al., 2023 (223) | The Pattern of Progression to First-Line Treatment with Dabrafenib and Trametinib in Patients with Unresectable or Metastatic, BRAF-Mutated, Cutaneous Melanoma: Results of the Observational T-WIN Study | 127/74 |  |
| Sadrolashrafi et al., 2023 (224) | Retreatment of Patients With Metastatic Cutaneous Melanoma Who Relapse After Elective Checkpoint Inhibitor Discontinuation After a Complete Remission | 5/5 |  |
| Crystal et al., 2022 (225) | Therapeutic Value of Sentinel Lymph Node Biopsy in Patients With Melanoma: A Randomized Clinical Trial | 479/344 |  |
| Pellacani et al., 2022 (226) | Effect of Reflectance Confocal Microscopy for Suspect Lesions on Diagnostic Accuracy in Melanoma: A Randomized Clinical Trial | 1608/1557 |  |
| Jørgensen et al., 2022 (227) | Can prophylactic incisional negative pressure wound therapy Reduce Wound Complications After Inguinal Lymph Node Dissection for Melanoma? Results from a Randomized Controlled Trial | 10/10 |  |
| Naeser et al., 2022 (228) | Quality of Life in the First Year of Follow-Up in a Randomized Multicenter Trial Assessing the Role of Imaging after Radical Surgery of Stage IIB-C and III Cutaneous Melanoma (TRIM Study) | 124/79 |  |
| Aung et al., 2022 (229) | Objective assessment of tumor infiltrating lymphocytes as a prognostic marker in melanoma using machine learning algorithms | 223/196 |  |
| Friedman et al., 2022 (230) | Ipilimumab alone or in combination with nivolumab in patients with advanced melanoma who have progressed or relapsed on PD-1 blockade: clinical outcomes and translational biomarker analyses | 15/4 |  |
| Jahn et al., 2022 (231) | Over-Detection of Melanoma-Suspect Lesions by a CE-Certified Smartphone App: Performance in Comparison to Dermatologists, 2D and 3D Convolutional Neural Networks in a Prospective Data Set of 1204 Pigmented Skin Lesions Involving Patients' Perception | 56/58 |  |
| Sanjuda et al., 2022 (232) | In-Depth Characterisation of Real-World Advanced Melanoma Patients Receiving Immunotherapies and/or Targeted Therapies: A Case Series | 26/15 |  |
| Zhou et al., 2022 (233) | Safety, activity, and pharmacokinetics of camrelizumab in advanced Asian melanoma patients: a phase I study | 17/19 |  |
| Yan et al., 2022 (234) | An Immune-Related Gene Pair Index Predicts Clinical Response and Survival Outcome of Immune Checkpoint Inhibitors in Melanoma | 91/75 |  |
| Aamdal et al., 2022 (235) | Health-related quality of life in patients with advanced melanoma treated with ipilimumab: prognostic implications and changes during treatment | 88/53 |  |
| Voglis et al., 2022 (236) | Maximal surgical tumour load reduction in immune-checkpoint inhibitor naïve patients with melanoma brain metastases correlates with prolonged survival | 80/41 |  |
| Aldenhoven et al., 2022 (237) | Sentinel lymph node mapping with superparamagnetic iron oxide for melanoma: a pilot study in healthy participants to establish an optimal MRI workflow protocol | 5/1 |  |
| Jakub et al., 2022 (238) | Association of tumor molecular factors with in-transit metastasis in primary cutaneous melanoma | 535/319 |  |
| Holmberg et al., 2022 (239) | The efficacy of immune checkpoint blockade for melanoma in-transit with or without nodal metastases - A multicenter cohort study | 161/126 |  |
| Wiens et al., 2021 (240) | Psychological Distress of Metastatic Melanoma Patients during Treatment with Immune Checkpoint Inhibitors: Results of a Prospective Study | 59/54 |  |
| Lan et al., 2021 (241) | Prospective study of clinical characteristics of melanoma patients with retinopathy caused by a high-dose interferon α-2b | 18/25 |  |
| MacLellan et al., 2021 (242) | The use of noninvasive imaging techniques in the diagnosis of melanoma: a prospective diagnostic accuracy study | 100/84 |  |
| LoRusso et al., 2021 (243) | Identifying treatment options for BRAFV600 wild-type metastatic melanoma: A SU2C/MRA genomics-enabled clinical trial | 14/15 |  |
| Herts et al., 2021 (244) | A prospective study of cancer-related benefit finding in uveal melanoma patients | 58/49 |  |
| Guadagni et al., 2021 (245) | A Prospective Study of Intraarterial Infusion Chemotherapy in Advanced Wild-Type BRAF Melanoma Patients | 20/42 |  |
| Blankenstein et al., 2021 (246) | Neoadjuvant Cytoreductive Treatment With BRAF/MEK Inhibition of Prior Unresectable Regionally Advanced Melanoma to Allow Complete Surgical Resection, REDUCTOR: A Prospective, Single-arm, Open-label Phase II Trial | 10/11 |  |
| Bakshi et al., 2021 (247) | Genomic Risk Score for Melanoma in a Prospective Study of Older Individuals | 5722/6990 |  |
| Gollrad et al., 2021 (248) | Quality of life and treatment-related burden during ocular proton therapy: a prospective trial of 131 patients with uveal melanoma | 66/65 |  |
| M de Meza et al., 2021 (249) | Adjuvant treatment for melanoma in clinical practice - Trial versus reality | 362/206 |  |
| Aamdal et al., 2021 (250) | Ipilimumab in a real-world population: A prospective Phase IV trial with long-term follow-up | 96/55 |  |
| Yamazaki et al., 2021 (251) | Prospective observational study of the efficacy of nivolumab in Japanese patients with advanced melanoma (CREATIVE study) | 72/52 |  |
| Watts et al., 2021 (252) | Association Between Melanoma Detected During Routine Skin Checks and Mortality | 1502/950 |  |
| Gómez Olivas et al., 2021 (253) | Sleep Duration and Cutaneous Melanoma Aggressiveness. A Prospective Observational Study in 443 Patients | 224/219 |  |
| Storkus et al., 2021 (254) | Dendritic cell vaccines targeting tumor blood vessel antigens in combination with dasatinib induce therapeutic immune responses in patients with checkpoint-refractory advanced melanoma | 8/7 |  |
| Reijers et al., 2021 (255) | Representativeness of the Index Lymph Node for Total Nodal Basin in Pathologic Response Assessment After Neoadjuvant Checkpoint Inhibitor Therapy in Patients With Stage III Melanoma | 48/34 |  |
| Becquart et al., 2021 (256) | Tolerance and Effectiveness of Targeted Therapies in Aged Patients with Metastatic Melanoma | 207/146 |  |
| Dalle et al., 2021 (257) | Long-term real-world experience with ipilimumab and non-ipilimumab therapies in advanced melanoma: the IMAGE study | 819/537 |  |
| Guitera et al., 2021 (258) | Efficiency of Detecting New Primary Melanoma Among Individuals Treated in a High-risk Clinic for Skin Surveillance | 340/253 |  |
| Saiag et al., 2021 (259) | Efficacy, safety and factors associated with disease progression in patients with unresectable (stage III) or distant metastatic (stage IV) BRAF V600-mutant melanoma: An open label, non-randomized, phase IIIb study of trametinib in combination with dabrafenib | 474/382 |  |
| Gulati et al., 2021 (260) | Preexisting immune-mediated inflammatory disease is associated with improved survival and increased toxicity in melanoma patients who receive immune checkpoint inhibitors | 295/188 |  |
| Pokorny et al., 2021 (261) | Real-world experience with elective discontinuation of PD-1 inhibitors at 1 year in patients with metastatic melanoma | 38/14 |  |
| Tarhini et al., 2021 (262) | A matching-adjusted indirect comparison of combination nivolumab plus ipilimumab with BRAF plus MEK inhibitors for the treatment of BRAF-mutant advanced melanoma | 206/108 |  |
| Patel et al., 2021 (263) | The utility of ICG fluorescence for sentinel lymph node identification in head and neck melanoma | 299/100 |  |
| Bulgarelli et al., 2021 (264) | Radiotherapy and High-Dose Interleukin-2: Clinical and Immunological Results of a Proof of Principle Study in Metastatic Melanoma and Renal Cell Carcinoma | 4/6 |  |
| Bai et al., 2021 (265) | Risk Models for Advanced Melanoma Patients Under Anti-PD-1 Monotherapy- Ad hoc Analyses of Pooled Data From Two Clinical Trial | 44/45 |  |
| Kobayashi et al., 2020 (266) | Pituitary dysfunction induced by immune checkpoint inhibitors is associated with better overall survival in both malignant melanoma and non-small cell lung carcinoma: a prospective study | 39/27 |  |
| Nomura et al., 2020 (267) | Multicenter prospective phase II trial of nivolumab in patients with unresectable or metastatic mucosal melanoma | 13/7 |  |
| Binkley et al., 2020 (268) | A prospective trial of adjuvant therapy for high-risk uveal melanoma: assessing 5-year survival outcomes | 16/17 |  |
| Postow et al., 2020 (269) | A Prospective, Phase 1 Trial of Nivolumab, Ipilimumab, and Radiotherapy in Patients with Advanced Melanoma | 12/8 |  |
| Boudewijns et al., 2020 (270) | Autologous monocyte-derived DC vaccination combined with cisplatin in stage III and IV melanoma patients: a prospective, randomized phase 2 trial | 36/18 |  |
| Barbosa de Carvalho er al., 2020 (271) | Near Infrared (NIR) Fluorescence is Not a Substitute for Lymphoscintigraphy and Gamma Probe for Melanoma Sentinel Node Detection: Results from a Prospective Trial | 62/59 |  |
| Dummer et al., 2020 (272) | Adjuvant dabrafenib plus trametinib versus placebo in patients with resected, BRAFV600-mutant, stage III melanoma (COMBI-AD): exploratory biomarker analyses from a randomised, phase 3 trial | 970/775 |  |
| Tang et al., 2020 (273) | Safety, Efficacy, and Biomarker Analysis of Toripalimab in Previously Treated Advanced Melanoma: Results of the POLARIS-01 Multicenter Phase II Trial | 57/70 |  |
| Ali et al., 2020 (274) | Correlation between initial tumour volume and treatment duration on Dabrafenib: observation study of subjects with BRAF mutant melanoma on the BRF112680 trial | 8/2 |  |
| Jansen et al., 2020 (275) | Salivary cortisol levels and anxiety in melanoma patients undergoing sentinel lymph node excision under local anesthesia versus general anesthesia: a prospective study | 36/26 |  |
| Salama et al., 2020 (276) | Ipilimumab and Radiation in Patients with High-risk Resected or Regionally Advanced Melanoma | 10/14 |  |
| Sinnamon et al., 2020 (277) | Survival Outcomes Following Lymph Node Biopsy in Thin Melanoma-A Propensity-Matched Analysis | 15118/13728 |  |
| Van Zejil et al., 2020 (278) | Real-world outcomes of advanced melanoma patients not represented in phase III trials | 1507/1029 |  |
| Von Schuckmann et al., 2020 (279) | Statins may reduce disease recurrence in patients with ulcerated primary melanoma | 410/290 |  |
| Asher et al., 2020 (280) | Real World Outcomes of Ipilimumab and Nivolumab in Patients with Metastatic Melanoma | 99/73 |  |
| Algazi et al., 2020 (281) | Intratumoral delivery of tavokinogene telseplasmid yields systemic immune responses in metastatic melanoma patients | 16/14 |  |
| Alves Wainstein et al., 2020 (282) | Assessment of retraction in surgical specimens in melanoma patients submitted to oncological amplification of margins | 85/60 |  |
| Liermann et al., 2020 (283) | Stereotactic Radiosurgery With Concurrent Immunotherapy in Melanoma Brain Metastases Is Feasible and Effective | 28/8 |  |
| Gill et al., 2020 (284) | Correlating Radiomic Features of Heterogeneity on CT with Circulating Tumor DNA in Metastatic Melanoma | 10/5 |  |
| Hadi et al., 2020 (285) | Stereotactic radiosurgery combined with targeted/ immunotherapy in patients with melanoma brain metastasis | 17/11 |  |
| Curti et al., 2020 (286) | Randomized phase II study of stereotactic body radiotherapy and interleukin-2 versus interleukin-2 in patients with metastatic melanoma | 34/10 |  |
| Holbrook et al., 2020 (287) | Intracranial antitumor activity with encorafenib plus binimetinib in patients with melanoma brain metastases: A case series | 14/10 |  |
| Ponti et al., 2020 (288) | First-Line Selective Internal Radiation Therapy in Patients with Uveal Melanoma Metastatic to the Liver | 11/11 |  |
| Wong et al., 2020 (289) | 18F-FDG PET/CT based spleen to liver ratio associates with clinical outcome to ipilimumab in patients with metastatic melanoma | 60/30 |  |
| Verver et al., 2020 (290) | The EORTC-DeCOG nomogram adequately predicts outcomes of patients with sentinel node-positive melanoma without the need for completion lymph node dissection | 425/267 |  |
| Ellison et al., 2019 (291) | Mohs micrographic surgery for melanoma: A prospective multicenter study | 327/191 |  |
| Gonsalves et al., 2019 (292) | A Prospective Phase II Trial of Radioembolization for Treatment of Uveal Melanoma Hepatic Metastasis | 22/26 |  |
| Phillips et al., 2019 (293) | Assessment of Accuracy of an Artificial Intelligence Algorithm to Detect Melanoma in Images of Skin Lesions | 222/279 |  |
| Luong et al., 2019 (294) | Reduction of lymphocele rate in patients undergoing sentinel node biopsy for melanoma by intraoperative placement of plant-based hemostatic powder: Results of a prospective trial | 57/59 |  |
| Bradbury et al., 2019 (295) | Circulating insulin-like growth factor I in relation to melanoma risk in the European prospective investigation into cancer and nutrition | 523/698 |  |
| Takayasu et al., 2019 (296) | Carbon-ion radiotherapy combined with chemotherapy for head and neck mucosal melanoma: Prospective observational study | 13/8 |  |
| Forschner et al., 2019 (297) | Tumor mutation burden and circulating tumor DNA in combined CTLA-4 and PD-1 antibody therapy in metastatic melanoma - results of a prospective biomarker study | 16/19 |  |
| Hauswald et al., 2019 (298) | Whole-brain helical tomotherapy with integrated boost for brain metastases in patients with malignant melanoma - final results of the BRAIN-RT trial | 4/3 |  |
| Henderson et al., 2019 (299) | Inguinal and Ilio-inguinal Lymphadenectomy in Management of Palpable Melanoma Lymph Node Metastasis: A Long-Term Prospective Evaluation of Morbidity and Quality of Life | 46/23 |  |
| Alvarez-Breckenridge et al., 2019 (300) | Upfront Surgical Resection of Melanoma Brain Metastases Provides a Bridge Toward Immunotherapy-Mediated Systemic Control | 94/48 |  |
| Banting et al., 2019 (301) | Negative Sentinel Lymph Node Biopsy in Patients with Melanoma: The Patient's Perspective | 56/46 |  |
| Czarnecka et al., 2019 (302) | Treatment Sequencing and Clinical Outcomes in BRAF-Positive and BRAF-Negative Unresectable and Metastatic Melanoma Patients Treated with New Systemic Therapies in Routine Practice | 133/120 |  |
| Ipenburg et al., 2019 (303) | External validation of a prognostic model to predict survival of patients with sentinel node-negative melanoma | 4131/3282 |  |
| Chin Lo et al., 2019 (304) | Reconstructive burden and financial implications of wider excision margins for invasive primary cutaneous melanoma | 621/563 |  |
| Lang et al., 2019 (305) | Clinical significance of signs of autoimmune colitis in 18F-fluorodeoxyglucose positron emission tomography-computed tomography of 100 stage-IV melanoma patients | 71/29 |  |
| Nijhuis et al., 2019 (306) | False-Positive Results and Incidental Findings with Annual CT or PET/CT Surveillance in Asymptomatic Patients with Resected Stage III Melanoma | 105/49 |  |
| Jansen et al., 2019 (307) | Discontinuation of anti-PD-1 antibody therapy in the absence of disease progression or treatment limiting toxicity: clinical outcomes in advanced melanoma | 113/70 |  |
| Grätz et al., 2019 (308) | Sequential Treatment With Targeted and Immune Checkpoint Therapy in Patients With BRAF Positive Metastatic Melanoma: The Importance of Timing? | 9/2 |  |
| De Giorgi et al., 2018 (309) | Propranolol for Off-label Treatment of Patients With Melanoma: Results From a Cohort Study | 33/20 |  |
| Forschner et al., 2018 (310) | Diagnostic accuracy of dermatofluoroscopy in cutaneous melanoma detection: results of a prospective multicentre clinical study in 476 pigmented lesions | 211/158 |  |
| Read et al., 2018 (311) | Intralesional PV-10 for the treatment of in-transit melanoma metastases-Results of a prospective, non-randomized, single center study | 27/18 |  |
| O’Leary et al., 2018 (312) | Survival outcomes and interval between lymphoscintigraphy and SLNB in cutaneous melanoma- findings of a large prospective cohort study | 521/494 |  |
| Dusingize et al., 2018 (313) | Smoking and Cutaneous Melanoma: Findings from the QSkin Sun and Health Cohort Study | 17697/21000 |  |
| Amini-Adle et al., 2018 (314) | Ineffective anti PD-1 therapy after BRAF inhibitor failure in advanced melanoma | 23/10 |  |
| Couto et al., 2018 (315) | Determining the False-Negative Rate Using Fluorescence Image-Assisted Sentinel Lymph Node Biopsy in Cutaneous Melanoma | 65/60 |  |
| Shoushtari et al., 2018 (316) | Measuring Toxic Effects and Time to Treatment Failure for Nivolumab Plus Ipilimumab in Melanoma | 32/32 |  |
| Pameijer et al., 2018 (317) | Indocyanine green and fluorescence lymphangiography for sentinel node identification in patients with melanoma | 43/44 |  |
| Du Four et al., 2018 (318) | Focal radiation necrosis of the brain in patients with melanoma brain metastases treated with pembrolizumab | 14/29 |  |
| Mooradian et al., 2018 (319) | A phase II study of combined therapy with a BRAF inhibitor (vemurafenib) and interleukin-2 (aldesleukin) in patients with metastatic melanoma | 3/3 |  |
| Read et al., 2018 (320) | Follow-Up Recommendations after Diagnosis of Primary Cutaneous Melanoma: A Population-Based Study in New South Wales, Australia | 1195/777 |  |
| Si et al., 2018 (321) | Vemurafenib in Chinese patients with BRAFV600 mutation-positive unresectable or metastatic melanoma: an open-label, multicenter phase I study | 21/25 |  |
| Yao et al., 2018 (322) | Efficacy and safety of primary surgery with postoperative radiotherapy in head and neck mucosal melanoma: a single-arm Phase II study | 21/13 |  |
| Kashani-Sabet et al., 2017 (323) | Prospective Validation of Molecular Prognostic Markers in Cutaneous Melanoma: A Correlative Analysis of E1690 | 167/81 |  |
| Rostas et al., 2017 (324) | Health-related quality of life during trans-arterial chemoembolization with drug-eluting beads loaded with doxorubicin (DEBDOX) for unresectable hepatic metastases from ocular melanoma | 13/7 |  |
| Patel et al., 2017 (325) | A phase II study of ipilimumab plus temozolomide in patients with metastatic melanoma | 45/19 |  |
| Williams et al., 2017 (326) | Phase 1 Study of Ipilimumab Combined With Whole Brain Radiation Therapy or Radiosurgery for Melanoma Patients With Brain Metastases | 12/4 |  |
| Davies et al., 2017 (327) | Dabrafenib plus trametinib in patients with BRAFV600-mutant melanoma brain metastases (COMBI-MB): a multicentre, multicohort, open-label, phase 2 trial | 72/53 |  |
| De la Cruz-Merino et al., 2017 (328) | Clinical features of serous retinopathy observed with cobimetinib in patients with BRAF-mutated melanoma treated in the randomized coBRIM study | 286/209 |  |
| Montaudié et al., 2017 (329) | Metformin monotherapy in melanoma: a pilot, open-label, prospective, and multicentric study indicates no benefit | 10/7 |  |
| Fabian et al., 2017 (330) | Primary photodynamic therapy with verteporfin for small pigmented posterior pole choroidal melanoma | 5/10 |  |
| Frankel et al., 2017 (331) | Digoxin Plus Trametinib Therapy Achieves Disease Control in BRAF Wild-Type Metastatic Melanoma Patients | 8/12 |  |
| Schreuer et al., 2017 (332) | Combination of dabrafenib plus trametinib for BRAF and MEK inhibitor pretreated patients with advanced BRAFV600-mutant melanoma: an open-label, single arm, dual-centre, phase 2 clinical trial | 15/10 |  |
| Weide et al., 2017 (333) | Combined treatment with ipilimumab and intratumoral interleukin-2 in pretreated patients with stage IV melanoma-safety and efficacy in a phase II study | 9/6 |  |
| Goldman et al., 2017 (334) | Outcomes in Melanoma Patients Treated with BRAF/MEK-Directed Therapy or Immune Checkpoint Inhibition Stratified by Clinical Trial versus Standard of Care | 214/104 |  |
| Jakub et al., 2017 (335) | Safety and Feasibility of Minimally Invasive Inguinal Lymph Node Dissection in Patients With Melanoma (SAFE-MILND): Report of a Prospective Multi-institutional Trial | 35/52 |  |
| Saiag et al., 2016 (336) | Prospective assessment of a gene signature potentially predictive of clinical benefit in metastatic melanoma patients following MAGE-A3 immunotherapeutic (PREDICT) | 58/65 |  |
| Rossi et al., 2016 (337) | Evaluation of the Upper Limb Lymphatic System: A Prospective Lymphoscintigraphic Study in Melanoma Patients and Healthy Controls | 8/8 |  |
| Hiniker et al., 2016 (338) | A Prospective Clinical Trial Combining Radiation Therapy With Systemic Immunotherapy in Metastatic Melanoma | 15/7 |  |
| Damude et al., 2016 (339) | The MELFO-Study: Prospective, Randomized, Clinical Trial for the Evaluation of a Stage-adjusted Reduced Follow-up Schedule in Cutaneous Melanoma Patients-Results after 1 Year | 87/93 |  |
| Rule et al., 2016 (340) | Results of NCCTG N0275 (Alliance) - a phase II trial evaluating resection followed by adjuvant radiation therapy for patients with desmoplastic melanoma | 10/10 |  |
| Eigentler et al., 2016 (341) | Adjuvant treatment with pegylated interferon α-2a versus low-dose interferon α-2a in patients with high-risk melanoma: a randomized phase III DeCOG trial | 476/433 |  |
| Chen et al., 2016 (342) | Clinical, Molecular, and Immune Analysis of Dabrafenib-Trametinib Combination Treatment for BRAF Inhibitor-Refractory Metastatic Melanoma: A Phase 2 Clinical Trial | 16/7 |  |
| Krogh et al., 2016 (343) | Prognostic and predictive value of YKL-40 in stage IIB-III melanoma | 758/476 |  |
| Goff et al., 2016 (344) | Randomized, Prospective Evaluation Comparing Intensity of Lymphodepletion Before Adoptive Transfer of Tumor-Infiltrating Lymphocytes for Patients With Metastatic Melanoma | 64/37 |  |
| Fang et al., 2016 (345) | Association of Vitamin D Levels With Outcome in Patients With Melanoma After Adjustment For C-Reactive Protein | 590/452 |  |
| Weber et al., 2016 (346) | Phase I/II Study of Metastatic Melanoma Patients Treated with Nivolumab Who Had Progressed after Ipilimumab | 60/32 |  |
| McMasters et al., 2016 (347) | Final Results of the Sunbelt Melanoma Trial: A Multi-Institutional Prospective Randomized Phase III Study Evaluating the Role of Adjuvant High-Dose Interferon Alfa-2b and Completion Lymph Node Dissection for Patients Staged by Sentinel Lymph Node Biopsy | 418/356 |  |
| Zenda et al., 2016 (348) | Phase II study of proton beam therapy as a nonsurgical approach for mucosal melanoma of the nasal cavity or para-nasal sinuses | 12/20 |  |
| Kibrité et al., 2015 (349) | Predictive factors for sentinel lymph nodes and non-sentinel lymph nodes metastatic involvement: a database study of 1,041 melanoma patients | 499/458 |  |
| Mohr et al., 2015 (350) | Intermittent High-Dose Intravenous Interferon Alfa-2b for Adjuvant Treatment of Stage III Melanoma: Final Analysis of a Randomized Phase III Dermatologic Cooperative Oncology Group Trial | 360/267 |  |
| Lawson et al., 2015 (351) | Randomized, Placebo-Controlled, Phase III Trial of Yeast-Derived Granulocyte-Macrophage Colony-Stimulating Factor (GM-CSF) Versus Peptide Vaccination Versus GM-CSF Plus Peptide Vaccination Versus Placebo in Patients With No Evidence of Disease After Complete Surgical Resection of Locally Advanced and/or Stage IV Melanoma: A Trial of the Eastern Cooperative Oncology Group-American College of Radiology Imaging Network Cancer Research Group (E4697) | 483/331 |  |
| C van Dijk et al., 2015 (352) | Serous Retinopathy Associated with Mitogen-Activated Protein Kinase Kinase Inhibition (Binimetinib) for Metastatic Cutaneous and Uveal Melanoma | 22/13 |  |
| Speijers et al., 2015 (353) | Tumor mitotic rate added to the equation: melanoma prognostic factors changed? : a single-institution database study on the prognostic value of tumor mitotic rate for sentinel lymph node status and survival of cutaneous melanoma patients | 231/222 |  |
| Ferrucci et al., 2015 (354) | Dacarbazine in combination with bevacizumab for the treatment of unresectable/metastatic melanoma: a phase II study | 24/13 |  |
| Algazi et al., 2015 (355) | The combination of axitinib followed by paclitaxel/carboplatin yields extended survival in advanced BRAF wild-type melanoma: results of a clinical/correlative prospective phase II clinical trial | 26/12 |  |
| Bol et al., 2014 (356) | Long overall survival after dendritic cell vaccination in metastatic uveal melanoma patients |  |  |
| Malvehy et al., 2014 (357) | Clinical performance of the Nevisense system in cutaneous melanoma detection: an international, multicentre, prospective and blinded clinical trial on efficacy and safety | 929/1013 |  |
| Ribas et al., 2014 (358) | Combination of vemurafenib and cobimetinib in patients with advanced BRAF(V600)-mutated melanoma: a phase 1b study | 77/52 |  |
| Jiang et al., 2014 (359) | Immunotherapy following regional chemotherapy treatment of advanced extremity melanoma | 16/17 |  |
| Fujiyama et al., 2014 (360) | Induction of cytotoxic T cells as a novel independent survival factor in malignant melanoma with percutaneous peptide immunization | 22/13 |  |
| T van der Ploeg et al., 2014 (361) | Outcome following sentinel node biopsy plus wide local excision versus wide local excision only for primary cutaneous melanoma: analysis of 5840 patients treated at a single institution | 3420/2420 |  |
| Wilson et al., 2014 (362) | Correlation of somatic mutations and clinical outcome in melanoma patients treated with Carboplatin, Paclitaxel, and sorafenib | 113/65 |  |
| Kelderman et al., 2014 (363) | Lactate dehydrogenase as a selection criterion for ipilimumab treatment in metastatic melanoma | 134/96 |  |
| Perier-Muzet et al., 2014 (364) | Melanoma patients under vemurafenib: prospective follow-up of melanocytic lesions by digital dermoscopy | 29/13 |  |
| Shi et al., 2014 (365) | Metastatic malignant melanoma: computed tomography-guided 125I seed implantation treatment | 16/8 |  |
| Azer et al., 2014 (366) | Patterns of response and progression in patients with BRAF-mutant melanoma metastatic to the brain who were treated with dabrafenib | 15/8 |  |
| Sanmamed et al., 2014 (367) | Relevance of MIA and S100 serum tumor markers to monitor BRAF inhibitor therapy in metastatic melanoma patients | 13/5 |  |
| Yuan et al., 2014 (368) | Pretreatment serum VEGF is associated with clinical response and overall survival in advanced melanoma patients treated with ipilimumab | 123/53 |  |
| Paulsen et al., 2014 (369) | Tumour response after hyperthermic isolated limb perfusion for locally advanced melanoma | 31/53 |  |
| Romero et al., 2014 (370) | Regulation of CD4(+)NKG2D(+) Th1 cells in patients with metastatic melanoma treated with sorafenib: role of IL-15Rα and NKG2D triggering | 35/28 |  |

1. Zhong J, Sun W, Hu T, Wang C, Yan W, Luo Z, et al. Comparative analysis of adjuvant therapy for stage III BRAF-mut melanoma: A real-world retrospective study from single center in China. Cancer Med. 2023 May;12(10):11475–82.

2. Piñero-Madrona A, Cerezuela-Fuentes P, Ruiz-Merino G, Martínez-Barba E, Ortiz-Reina S, Martínez-Ortiz MJ, et al. Lower limb cutaneous melanoma surgery: location matters. Arch Dermatol Res. 2023 Sep;315(7):1971–8.

3. Van Not OJ, van den Eertwegh AJM, Haanen JB, van Rijn RS, Aarts MJB, van den Berkmortel FWPJ, et al. Response to checkpoint inhibition and targeted therapy in melanoma patients with concurrent haematological malignancies. Eur J Cancer Oxf Engl 1990. 2023 Jun;186:27–37.

4. Schumann K, Mauch C, Klespe KC, Loquai C, Nikfarjam U, Schlaak M, et al. Real-world outcomes using PD-1 antibodies and BRAF + MEK inhibitors for adjuvant melanoma treatment from 39 skin cancer centers in Germany, Austria and Switzerland. J Eur Acad Dermatol Venereol JEADV. 2023 May;37(5):894–906.

5. Bhave P, Hong A, Lo SN, Johnson R, Mangana J, Johnson DB, et al. Efficacy and toxicity of adjuvant radiotherapy in recurrent melanoma after adjuvant immunotherapy. J Immunother Cancer. 2023 Mar;11(3):e006629.

6. Koch EAT, Petzold A, Wessely A, Dippel E, Eckstein M, Gesierich A, et al. Liver-directed treatment is associated with improved survival and increased response to immune checkpoint blockade in metastatic uveal melanoma: results from a retrospective multicenter trial. Front Med. 2023 Oct;17(5):878–88.

7. Zhong J, Zou Z, Hu T, Sun W, Wang C, Yan W, et al. Survival impact of immediate complete lymph node dissection for Chinese acral and cutaneous melanoma with micrometastasis in sentinel nodes: a retrospective study. Clin Exp Med. 2023 Nov;23(7):4003–10.

8. Medri M, Savoia F, Foca F, Miserocchi A, Quaglino P, Rubatto M, et al. A retrospective observational study on cutaneous adverse events induced by immune checkpoint inhibitors. Ital J Dermatol Venereol. 2023 Dec;158(6):437–44.

9. Bai X, Shaheen A, Grieco C, d’Arienzo PD, Mina F, Czapla JA, et al. Dabrafenib plus trametinib versus anti-PD-1 monotherapy as adjuvant therapy in BRAF V600-mutant stage III melanoma after definitive surgery: a multicenter, retrospective cohort study. EClinicalMedicine. 2023 Nov;65:102290.

10. Bravo AI, Aris M, Panouillot M, Porto M, Dieu-Nosjean MC, Teillaud JL, et al. HEV-associated dendritic cells are observed in metastatic tumor-draining lymph nodes of cutaneous melanoma patients with longer distant metastasis-free survival after adjuvant immunotherapy. Front Immunol. 2023;14:1231734.

11. Cui C, Chen Y, Luo Z, Zou Z, Jiang Y, Pan H, et al. Safety and efficacy of Pucotenlimab (HX008) - a humanized immunoglobulin G4 monoclonal antibody in patients with locally advanced or metastatic melanoma: a single-arm, multicenter, phase II study. BMC Cancer. 2023 Feb 6;23(1):121.

12. Hedderson MM, Asgari MM, Xu F, Quesenberry CP, Sridhar S, Geier J, et al. Rates of malignancies among patients with moderate to severe atopic dermatitis: a retrospective cohort study. BMJ Open. 2023 Mar 10;13(3):e071172.

13. Ayati N, Jamshidi-Araghi Z, Hoellwerth M, Schweighofer-Zwink G, Hitzl W, Koelblinger P, et al. Predictive value and accuracy of [18F]FDG PET/CT modified response criteria for checkpoint immunotherapy in patients with advanced melanoma. Eur J Nucl Med Mol Imaging. 2023 Jul;50(9):2715–26.

14. Quildrian SD, Nardi WS, Scasso V, Novas C, Bella Quero L, Silva C. Current management of patients with cutaneous melanoma with a positive sentinel lymph node. Medicina (Mex). 2023;83(3):376–83.

15. Zhang X, Lin Z, Li M, Gai Y, Zheng H, Fan L, et al. Melanin-targeted [18F]-PFPN PET imaging for prognosticating patients with melanoma. Eur J Nucl Med Mol Imaging. 2023 Aug;50(10):3062–71.

16. Kähler KC, Hüning S, Nashan D, Meiss F, Rafei-Shamsabadi DA, Rissmann H, et al. Preferences of German and Swiss melanoma patients for toxicities versus melanoma recurrence during adjuvant treatment (GERMELATOX-A-trial). J Cancer Res Clin Oncol. 2023 Oct;149(13):11705–18.

17. Broman KK, Hughes TM, Bredbeck BC, Sun J, Kirichenko D, Carr MJ, et al. International Center-Level Variation in Utilization of Completion Lymph Node Dissection and Adjuvant Systemic Therapy for Sentinel Lymph Node-Positive Melanoma at Major Referral Centers. Ann Surg. 2023 May 1;277(5):e1106–15.

18. Brown LJ, da Silva IP, Moujaber T, Gao B, Hui R, Gurney H, et al. Five-year survival and clinical correlates among patients with advanced non-small cell lung cancer, melanoma and renal cell carcinoma treated with immune check-point inhibitors in Australian tertiary oncology centres. Cancer Med. 2023 Mar;12(6):6788–801.

19. Afrăsânie VA, Alexa-Stratulat T, Gafton B, Froicu EM, Sur D, Lungulescu CV, et al. Real Check RIO: A Real-World Analysis of Nivolumab in First Line Metastatic Melanoma Assessing Efficacy, Safety and Predictive Factors. Cancers. 2023 Feb 16;15(4):1265.

20. Augustin RC, Huang Z, Ding F, Zhai S, McArdle J, Santisi A, et al. Metformin is associated with improved clinical outcomes in patients with melanoma: a retrospective, multi-institutional study. Front Oncol. 2023;13:1075823.

21. Nardin C, Hennemann A, Diallo K, Funck-Brentano E, Puzenat E, Heidelberger V, et al. Efficacy of Immune Checkpoint Inhibitor (ICI) Rechallenge in Advanced Melanoma Patients’ Responders to a First Course of ICI: A Multicenter National Retrospective Study of the French Group of Skin Cancers (Groupe de Cancérologie Cutanée, GCC). Cancers. 2023 Jul 10;15(14):3564.

22. Kalantari F, Mirshahvalad SA, Hoellwerth M, Schweighofer-Zwink G, Huber-Schönauer U, Hitzl W, et al. Prognostic Value of Baseline 18F-FDG PET/CT to Predict Brain Metastasis Development in Melanoma Patients. Cancers. 2023 Dec 26;16(1):127.

23. Tomsitz D, Ruf T, Heppt M, Staeger R, Ramelyte E, Dummer R, et al. Tebentafusp in Patients with Metastatic Uveal Melanoma: A Real-Life Retrospective Multicenter Study. Cancers. 2023 Jun 30;15(13):3430.

24. Dimitriou F, Namikawa K, Reijers ILM, Buchbinder EI, Soon JA, Zaremba A, et al. Single-agent anti-PD-1 or combined with ipilimumab in patients with mucosal melanoma: an international, retrospective, cohort study. Ann Oncol Off J Eur Soc Med Oncol. 2022 Sep;33(9):968–80.

25. Bhave P, Ahmed T, Lo SN, Shoushtari A, Zaremba A, Versluis JM, et al. Efficacy of anti-PD-1 and ipilimumab alone or in combination in acral melanoma. J Immunother Cancer. 2022 Jul;10(7):e004668.

26. Serra-Bellver P, Versluis JM, Oberoi HK, Zhou C, Slattery TD, Khan Y, et al. Real-world outcomes with ipilimumab and nivolumab in advanced melanoma: a multicentre retrospective study. Eur J Cancer Oxf Engl 1990. 2022 Nov;176:121–32.

27. Jones J, Lucey R, Corrie P. Retrospective review of outcomes associated with metastatic melanoma patients treated with 1st-line BRAF-targeted therapy. Pigment Cell Melanoma Res. 2022 Nov;35(6):595–604.

28. Salzmann M, Pawlowski J, Loquai C, Rafei-Shamsabadi DA, Meiss F, Ugurel S, et al. MEK inhibitors for pre-treated, NRAS-mutated metastatic melanoma: A multi-centre, retrospective study. Eur J Cancer Oxf Engl 1990. 2022 May;166:24–32.

29. Salaün H, de Koning L, Saint-Ghislain M, Servois V, Ramtohul T, Garcia A, et al. Nivolumab plus ipilimumab in metastatic uveal melanoma: a real-life, retrospective cohort of 47 patients. Oncoimmunology. 2022;11(1):2116845.

30. Kuzmanovszki D, Kiss N, Tóth B, Kerner T, Tóth V, Szakonyi J, et al. Anti-PD-1 Monotherapy in Advanced Melanoma-Real-World Data from a 77-Month-Long Retrospective Observational Study. Biomedicines. 2022 Jul 19;10(7):1737.

31. Pandya R, Basra M, Twohig E, McVeigh K. Can we reduce excision margins for head and neck melanoma? A 12-year retrospective study. Br J Oral Maxillofac Surg. 2022 Feb;60(2):134–9.

32. Eroglu Z, Broman KK, Thompson JF, Nijhuis A, Hieken TJ, Kottschade L, et al. Outcomes with adjuvant anti-PD-1 therapy in patients with sentinel lymph node-positive melanoma without completion lymph node dissection. J Immunother Cancer. 2022 Aug;10(8):e004417.

33. Williams TS, Tallon B, Adams BM. Melanoma sentinel lymph node biopsy and completion lymph node dissection: A regional hospital experience. J Plast Reconstr Aesthetic Surg JPRAS. 2022 Feb;75(2):730–6.

34. Broman KK, Richman J, Bhatia S. Evidence and implementation gaps in management of sentinel node-positive melanoma in the United States. Surgery. 2022 Jul;172(1):226–33.

35. Montgomery KB, Correya TA, Broman KK. Real-World Adherence to Nodal Surveillance for Sentinel Lymph Node-Positive Melanoma. Ann Surg Oncol. 2022 Sep;29(9):5961–8.

36. Kim JH, Lee CR, Kwon HJ, Oh DY, Jun YJ, Rhie JW, et al. Two-team-approached free flap reconstruction for plantar malignant melanoma: An observational (STROBE-compliant) trial. Medicine (Baltimore). 2022 Jul 29;101(30):e29442.

37. Googe PB, Theocharis S, Pergaris A, Li H, Yan Y, McKenna EF, et al. Theragnostic significance of tumor-infiltrating lymphocytes and Ki67 in BRAFV600-mutant metastatic melanoma (BRIM-3 trial). Curr Probl Cancer. 2022 Jun;46(3):100862.

38. Homan M, Warrier G, Lao CD, Yentz S, Kraft S, Fecher LA. Treatment related toxicities with combination BRAF and MEK inhibitor therapy in resected stage III melanoma. Front Oncol. 2022;12:855794.

39. Tropea S, Del Fiore P, Maurichi A, Patuzzo R, Santinami M, Ribero S, et al. The role of sentinel node tumor burden in modeling the prognosis of melanoma patients with positive sentinel node biopsy: an Italian melanoma intergroup study (N = 2,086). BMC Cancer. 2022 Jun 3;22(1):610.

40. Tolstrup LK, Pappot H, Bastholt L, Möller S, Dieperink KB. Impact of patient-reported outcomes on symptom monitoring during treatment with checkpoint inhibitors: health-related quality of life among melanoma patients in a randomized controlled trial. J Patient-Rep Outcomes. 2022 Jan 21;6(1):8.

41. Placzke J, Teterycz P, Quaglino P, Cybulska-Stopa B, Tucci M, Rubatto M, et al. The Analysis of Trends in Survival for Patients with Melanoma Brain Metastases with Introduction of Novel Therapeutic Options before the Era of Combined Immunotherapy-Multicenter Italian-Polish Report. Cancers. 2022 Nov 23;14(23):5763.

42. Lalonde E, Ewens K, Richards-Yutz J, Ebrahimzedeh J, Terai M, Gonsalves CF, et al. Improved Uveal Melanoma Copy Number Subtypes Including an Ultra-High-Risk Group. Ophthalmol Sci. 2022 Jun;2(2):100121.

43. Darabi S, Elliott A, Braxton DR, Zeng J, Hodges K, Poorman K, et al. Transcriptional Profiling of Malignant Melanoma Reveals Novel and Potentially Targetable Gene Fusions. Cancers. 2022 Mar 15;14(6):1505.

44. Lechner M, Takahashi Y, Turri-Zanoni M, Ferrari M, Liu J, Counsell N, et al. International Multicenter Study of Clinical Outcomes of Sinonasal Melanoma Shows Survival Benefit for Patients Treated with Immune Checkpoint Inhibitors and Potential Improvements to the Current TNM Staging System. J Neurol Surg Part B Skull Base. 2023 Aug;84(4):307–19.

45. Qian DC, Kleber T, Brammer B, Xu KM, Switchenko JM, Janopaul-Naylor JR, et al. Effect of immunotherapy time-of-day infusion on overall survival among patients with advanced melanoma in the USA (MEMOIR): a propensity score-matched analysis of a single-centre, longitudinal study. Lancet Oncol. 2021 Dec;22(12):1777–86.

46. Formozo AA, Gomes JR, Schmerling RA, Buzaid AC. Retrospective Analysis of Rechallenge with Ipilimumab in Patients with Metastatic Melanoma. J Skin Cancer. 2021;2021:5531864.

47. Rose AAN, Armstrong SM, Hogg D, Butler MO, Saibil SD, Arteaga DP, et al. Biologic subtypes of melanoma predict survival benefit of combination anti-PD1+anti-CTLA4 immune checkpoint inhibitors versus anti-PD1 monotherapy. J Immunother Cancer. 2021 Jan;9(1):e001642.

48. Versluis JM, Reijers ILM, Rozeman EA, Menzies AM, van Akkooi ACJ, Wouters MW, et al. Neoadjuvant ipilimumab plus nivolumab in synchronous clinical stage III melanoma. Eur J Cancer Oxf Engl 1990. 2021 May;148:51–7.

49. Aglietta M, Chiarion-Sileni V, Fava P, Guidoboni M, Depenni R, Minisini A, et al. Retrospective Chart Review of Dabrafenib Plus Trametinib in Patients with Metastatic BRAF V600-Mutant Melanoma Treated in the Individual Patient Program (DESCRIBE Italy). Target Oncol. 2021 Nov;16(6):789–99.

50. Phillips WJ, Baghai T, Ong M, Lo B, Ibrahim AM, Smith TKT, et al. A Contemporary Report of Clinical Outcomes in Patients with Melanoma Brain Metastases. Curr Oncol Tor Ont. 2021 Jan 13;28(1):428–39.

51. Yu SY, Mckavanagh D, McPherson I, Walpole E, Atkinson V, Hollingworth S. Survival of advanced melanoma patients treated with immunotherapy and targeted therapy: A real-world study. Pharmacoepidemiol Drug Saf. 2021 Oct;30(10):1371–9.

52. Crosby BJ, Lopez P, Galvão DA, Newton RU, Taaffe DR, Meniawy TM, et al. Associations of Physical Activity and Exercise with Health-related Outcomes in Patients with Melanoma During and After Treatment: A Systematic Review. Integr Cancer Ther. 2021;20:15347354211040757.

53. Mejbel HA, Torres-Cabala CA, Milton DR, Ivan D, Feldmeyer L, Namikawa K, et al. Prognostic significance of acral lentiginous histologic type in T1 melanoma. Mod Pathol Off J U S Can Acad Pathol Inc. 2021 Mar;34(3):572–83.

54. Monestier S, Dalle S, Mortier L, Dutriaux C, Dalac-Rat S, Meyer N, et al. Effectiveness and safety of nivolumab in patients with advanced melanoma: A multicenter, observational study. Int J Cancer. 2021 Jun 1;148(11):2789–98.

55. Ogata D, Haydu LE, Glitza IC, Patel SP, Tawbi HA, McQuade JL, et al. The efficacy of anti-programmed cell death protein 1 therapy among patients with metastatic acral and metastatic mucosal melanoma. Cancer Med. 2021 Apr;10(7):2293–9.

56. Gibney GT, Zaemes J, Shand S, Shah NJ, Swoboda D, Gardner K, et al. PET/CT scan and biopsy-driven approach for safe anti-PD-1 therapy discontinuation in patients with advanced melanoma. J Immunother Cancer. 2021 Oct;9(10):e002955.

57. Broman KK, Bettampadi D, Pérez-Morales J, Sun J, Kirichenko D, Carr MJ, et al. Surveillance of Sentinel Node-Positive Melanoma Patients Who Receive Adjuvant Therapy Without Undergoing Completion Lymph Node Dissection. Ann Surg Oncol. 2021 Nov;28(12):6978–85.

58. Rai P, Shen C, Kolodney J, Kelly KM, Scott VG, Sambamoorthi U. Factors associated with immune checkpoint inhibitor use among older adults with late-stage melanoma: A population-based study. Medicine (Baltimore). 2021 Feb 19;100(7):e24782.

59. Jiang C, Kleber TJ, Switchenko JM, Khan MK. Single institutional outcomes of whole brain radiotherapy for metastatic melanoma brain metastases. Radiat Oncol Lond Engl. 2021 Feb 8;16(1):31.

60. Madonna G, Masucci GV, Capone M, Mallardo D, Grimaldi AM, Simeone E, et al. Clinical Categorization Algorithm (CLICAL) and Machine Learning Approach (SRF-CLICAL) to Predict Clinical Benefit to Immunotherapy in Metastatic Melanoma Patients: Real-World Evidence from the Istituto Nazionale Tumori IRCCS Fondazione Pascale, Napoli, Italy. Cancers. 2021 Aug 19;13(16):4164.

61. Matsui Y, Sasaki J, Takatsuka S, Takenouchi T. Observation policy for sentinel node metastasis of melanoma: Comparative study with completion lymph node dissection in Japanese patients. J Dermatol. 2021 Aug;48(8):1221–8.

62. Ressler JM, Karasek M, Koch L, Silmbrod R, Mangana J, Latifyan S, et al. Real-life use of talimogene laherparepvec (T-VEC) in melanoma patients in centers in Austria, Switzerland and Germany. J Immunother Cancer. 2021 Feb;9(2):e001701.

63. Pescarmona R, Mouton W, Walzer T, Dalle S, Eberhardt A, Brengel-Pesce K, et al. Evaluation of TTV replication as a biomarker of immune checkpoint inhibitors efficacy in melanoma patients. PloS One. 2021;16(8):e0255972.

64. El Sharouni MA, Laeijendecker AE, Suijkerbuijk KP, Witkamp AJ, Sigurdsson V, van Diest PJ, et al. High discordance rate in assessing sentinel node positivity in cutaneous melanoma: Expert review may reduce unjustified adjuvant treatment. Eur J Cancer Oxf Engl 1990. 2021 May;149:105–13.

65. Di Guardo L, Randon G, Corti F, Vallacchi V, Raimondi A, Fucà G, et al. Liquid Biopsy and Radiological Response Predict Outcomes Following Discontinuation of Targeted Therapy in Patients with BRAF Mutated Melanoma. The Oncologist. 2021 Dec;26(12):1079–84.

66. Moore MR, Friesner ID, Rizk EM, Fullerton BT, Mondal M, Trager MH, et al. Automated digital TIL analysis (ADTA) adds prognostic value to standard assessment of depth and ulceration in primary melanoma. Sci Rep. 2021 Feb 2;11(1):2809.

67. Bouchereau S, Chaplain L, Fort M, Beauchet A, Sidibé T, Chapalain M, et al. Impact of prior treatment with immune checkpoint inhibitors on dacarbazine efficacy in metastatic melanoma. Br J Cancer. 2021 Sep;125(7):948–54.

68. Mangin MA, Boespflug A, Maucort Boulch D, Vacheron CH, Carpentier I, Thomas L, et al. Decreased survival in patients treated by chemotherapy after targeted therapy compared to immunotherapy in metastatic melanoma. Cancer Med. 2021 May;10(10):3155–64.

69. Flaus A, Habouzit V, De Leiris N, Vuillez JP, Leccia MT, Perrot JL, et al. FDG PET biomarkers for prediction of survival in metastatic melanoma prior to anti-PD1 immunotherapy. Sci Rep. 2021 Sep 22;11(1):18795.

70. Koelblinger P, Hoellwerth M, Dernoscheg MT, Koch L, Richtig E, Wanner M, et al. Adjuvant anti-PD-1 antibody treatment in stage III/IV melanoma: real-world experience and health economic considerations. J Dtsch Dermatol Ges J Ger Soc Dermatol JDDG. 2021 Aug;19(8):1186–98.

71. Zanoni DK, Stambuk HE, Madajewski B, Montero PH, Matsuura D, Busam KJ, et al. Use of Ultrasmall Core-Shell Fluorescent Silica Nanoparticles for Image-Guided Sentinel Lymph Node Biopsy in Head and Neck Melanoma: A Nonrandomized Clinical Trial. JAMA Netw Open. 2021 Mar 1;4(3):e211936.

72. Tian H, Wang X, Lian B, Yan X, Si L, Chi Z, et al. Safety Profile of Immunotherapy Combined With Antiangiogenic Therapy in Patients With Melanoma: Analysis of Three Clinical Studies. Front Pharmacol. 2021;12:747416.

73. Tejera-Vaquerizo A, Boada A, Ribero S, Puig S, Paradela S, Moreno-Ramírez D, et al. Sentinel Lymph Node Biopsy vs. Observation in Thin Melanoma: A Multicenter Propensity Score Matching Study. J Clin Med. 2021 Dec 15;10(24):5878.

74. Kelly D, Rose AAN, Muniz TP, Hogg D, Butler MO, Saibil SD, et al. Development of a Metastatic Uveal Melanoma Prognostic Score (MUMPS) for Use in Patients Receiving Immune Checkpoint Inhibitors. Cancers. 2021 Jul 20;13(14):3640.

75. Ksienski D, Truong PT, Croteau NS, Chan A, Sonke E, Patterson T, et al. Time to Treatment With Nivolumab or Pembrolizumab for Patients With Advanced Melanoma in Everyday Practice. Cureus. 2021 Nov;13(11):e19835.

76. Lopez-Obregon B, Barreto MP, Fyfe A, McKinnon G, Webb C, Temple-Oberle C. Evaluation of Intra-Lesional Interleukin 2 for the Treatment of In-Transit Melanoma Disease: L’évaluation de l’interleukine-2 intralésionnelle pour traiter les mélanomes en transit. Plast Surg Oakv Ont. 2021 Feb;29(1):4–9.

77. Orlova KV, Ledin EV, Zhukova NV, Orlova RV, Karabina EV, Volkonskiy MV, et al. Real-World Experience with Targeted Therapy in BRAF Mutant Advanced Melanoma Patients: Results from a Multicenter Retrospective Observational Study Advanced Melanoma in Russia (Experience) (ADMIRE). Cancers. 2021 May 21;13(11):2529.

78. Kleemann J, Jäger M, Valesky E, Kippenberger S, Kaufmann R, Meissner M. Real-World Experience of Talimogene Laherparepvec (T-VEC) in Old and Oldest-Old Patients with Melanoma: A Retrospective Single Center Study. Cancer Manag Res. 2021;13:5699–709.

79. Wu S, Yuan C, Chen L, Guo L, Chen Y, Peng Z, et al. Survival Benefits of Anti-PD-1 Therapy in Combination With Radiotherapy in Chinese Melanoma Patients With Brain Metastasis. Front Oncol. 2021;11:646328.

80. Valentin J, Ferté T, Dorizy-Vuong V, Dousset L, Prey S, Dutriaux C, et al. Real-World Survival in Patients with Metastatic Melanoma after Discontinuation of Anti-PD-1 Immunotherapy for Objective Response or Adverse Effects: A Retrospective Study. J Oncol. 2021;2021:5524685.

81. Hribernik N, Boc M, Ocvirk J, Knez-Arbeiter J, Mesti T, Ignjatovic M, et al. Retrospective analysis of treatment-naive Slovenian patients with metastatic melanoma treated with pembrolizumab - real-world experience. Radiol Oncol. 2020 Jan 19;54(1):119–27.

82. Brown LJ, Weppler A, Bhave P, Allayous C, Patrinely JR, Ott P, et al. Combination anti-PD1 and ipilimumab therapy in patients with advanced melanoma and pre-existing autoimmune disorders. J Immunother Cancer. 2021 May;9(5):e002121.

83. Ridolfi L, De Rosa F, Petracci E, Tanda ET, Marra E, Pigozzo J, et al. Anti-PD1 antibodies in patients aged ≥ 75 years with metastatic melanoma: A retrospective multicentre study. J Geriatr Oncol. 2020 Apr;11(3):515–22.

84. Berger M, Amini-Adlé M, Maucort-Boulch D, Robinson P, Thomas L, Dalle S, et al. Left ventricular ejection fraction decrease related to BRAF and/or MEK inhibitors in metastatic melanoma patients: A retrospective analysis. Cancer Med. 2020 Apr;9(8):2611–20.

85. Ziegler JS, Kroeze S, Hilbers ML, Imhof L, Guckenberger M, Levesque MP, et al. Toxicity of combined targeted therapy and concurrent radiotherapy in metastatic melanoma patients: a single-center retrospective analysis. Melanoma Res. 2020 Dec;30(6):552–61.

86. Mandel VD, Medri M, Manganoni AM, Pavoni L, De Rosa F, Ribero S, et al. Long-term vemurafenib therapy in advanced melanoma patients: cutaneous toxicity and prognostic implications. J Dermatol Treat. 2022 May;33(3):1368–75.

87. Spillane S, Baxi S, Torres AZ, Lenis D, Freedman AN, Mariotto AB, et al. Organ Dysfunction in Patients with Advanced Melanoma Treated with Immune Checkpoint Inhibitors. The Oncologist. 2020 Nov;25(11):e1753–62.

88. Comito F, Leslie I, Boos L, Furness A, Pickering L, Turajlic S, et al. Oligoprogression After Checkpoint Inhibition in Metastatic Melanoma Treated With Locoregional Therapy: A Single-center Retrospective Analysis. J Immunother Hagerstown Md 1997. 2020 Oct;43(8):250–5.

89. Suo A, Chan Y, Beaulieu C, Kong S, Cheung WY, Monzon JG, et al. Anti-PD1-Induced Immune-Related Adverse Events and Survival Outcomes in Advanced Melanoma. The Oncologist. 2020 May;25(5):438–46.

90. Nakamura Y, Namikawa K, Yoshino K, Yoshikawa S, Uchi H, Goto K, et al. Anti-PD1 checkpoint inhibitor therapy in acral melanoma: a multicenter study of 193 Japanese patients. Ann Oncol Off J Eur Soc Med Oncol. 2020 Sep;31(9):1198–206.

91. Balatoni T, Ladányi A, Fröhlich G, Czirbesz K, Kovács P, Pánczél G, et al. Biomarkers Associated with Clinical Outcome of Advanced Melanoma Patients Treated with Ipilimumab. Pathol Oncol Res POR. 2020 Jan;26(1):317–25.

92. Boada A, Tejera-Vaquerizo A, Ribero S, Puig S, Moreno-Ramírez D, Quaglino P, et al. Factors associated with sentinel lymph node status and prognostic role of completion lymph node dissection for thick melanoma. Eur J Surg Oncol J Eur Soc Surg Oncol Br Assoc Surg Oncol. 2020 Feb;46(2):263–71.

93. Aiken TJ, Stahl CC, Schwartz PB, Barrett J, Acher AW, Lemaster D, et al. Sentinel lymph node biopsy is associated with increased cost in higher risk thin melanoma. J Surg Oncol. 2021 Jan;123(1):104–9.

94. Betof Warner A, Palmer JS, Shoushtari AN, Goldman DA, Panageas KS, Hayes SA, et al. Long-Term Outcomes and Responses to Retreatment in Patients With Melanoma Treated With PD-1 Blockade. J Clin Oncol Off J Am Soc Clin Oncol. 2020 May 20;38(15):1655–63.

95. Uppal A, Stern S, Thompson JF, Foshag L, Mizzollo N, Nieweg OE, et al. Regional Node Basin Recurrence in Melanoma Patients: More Common After Node Dissection for Macroscopic Rather than Clinically Occult Nodal Disease. Ann Surg Oncol. 2020 Jun;27(6):1970–7.

96. Warburton L, Calapre L, Pereira MR, Reid A, Robinson C, Amanuel B, et al. Circulating Tumour DNA in Advanced Melanoma Patients Ceasing PD1 Inhibition in the Absence of Disease Progression. Cancers. 2020 Nov 23;12(11):3486.

97. Rauwerdink DJW, Molina G, Frederick DT, Sharova T, Carmichael H, Boland GM. Adjuvant Therapy Failure Patterns in the Modern Era of Melanoma Management. Ann Surg Oncol. 2020 Dec;27(13):5128–36.

98. Turiello R, Capone M, Giannarelli D, Morretta E, Monti MC, Madonna G, et al. Serum CD73 is a prognostic factor in patients with metastatic melanoma and is associated with response to anti-PD-1 therapy. J Immunother Cancer. 2020 Dec;8(2):e001689.

99. Atkinson V, Sandhu S, Hospers G, Long GV, Aglietta M, Ferrucci PF, et al. Dabrafenib plus trametinib is effective in the treatment of BRAF V600-mutated metastatic melanoma patients: analysis of patients from the dabrafenib plus trametinib Named Patient Program (DESCRIBE II). Melanoma Res. 2020 Jun;30(3):261–7.

100. Verver D, Rekkas A, Garbe C, van Klaveren D, van Akkooi ACJ, Rutkowski P, et al. The EORTC-DeCOG nomogram adequately predicts outcomes of patients with sentinel node-positive melanoma without the need for completion lymph node dissection. Eur J Cancer Oxf Engl 1990. 2020 Jul;134:9–18.

101. Boada A, Tejera-Vaquerizo A, Ribero S, Puig S, Moreno-Ramírez D, Osella-Abate S, et al. Age as a prognostic factor in thick and ultrathick melanomas without lymph node metastasis. J Eur Acad Dermatol Venereol JEADV. 2020 Sep;34(9):e513–7.

102. Rajeshuni N, Zubair T, Ludwig CA, Moshfeghi DM, Mruthyunjaya P. Evaluation of Racial, Ethnic, and Socioeconomic Associations With Treatment and Survival in Uveal Melanoma, 2004-2014. JAMA Ophthalmol. 2020 Aug 1;138(8):876–84.

103. Eisendle K, Weinlich G, Ebner S, Forstner M, Reider D, Zelle-Rieser C, et al. Combining chemotherapy and autologous peptide-pulsed dendritic cells provides survival benefit in stage IV melanoma patients. J Dtsch Dermatol Ges J Ger Soc Dermatol JDDG. 2020 Nov;18(11):1270–7.

104. Amaral T, Kiecker F, Schaefer S, Stege H, Kaehler K, Terheyden P, et al. Combined immunotherapy with nivolumab and ipilimumab with and without local therapy in patients with melanoma brain metastasis: a DeCOG* study in 380 patients. J Immunother Cancer. 2020 Mar;8(1):e000333.

105. Takahashi A, Namikawa K, Ogata D, Nakano E, Jinnai S, Nakama K, et al. Real-world efficacy and safety data of nivolumab and ipilimumab combination therapy in Japanese patients with advanced melanoma. J Dermatol. 2020 Nov;47(11):1267–75.

106. Cybulska-Stopa B, Rogala P, Czarnecka AM, Ługowska I, Teterycz P, Galus Ł, et al. Efficacy of ipilimumab after anti-PD-1 therapy in sequential treatment of metastatic melanoma patients - Real world evidence. Adv Med Sci. 2020 Sep;65(2):316–23.

107. Yamazaki K, Nishii R, Maeda T, Makishima H, Kasuya G, Chang T, et al. Assessment of SPECT-CT fusion images and semi-quantitative evaluation using SUV in 123I-IMP SPECT in patients with choroidal melanoma. Ann Nucl Med. 2020 Nov;34(11):864–72.

108. Peng Y, Chu Y, Chen Z, Zhou W, Wan S, Xiao Y, et al. Combining texture features of whole slide images improves prognostic prediction of recurrence-free survival for cutaneous melanoma patients. World J Surg Oncol. 2020 Jun 16;18(1):130.

109. Huang K, Misra S, Lemini R, Chen Y, Speicher LL, Dawson NL, et al. Completion lymph node dissection in patients with sentinel lymph node positive cutaneous head and neck melanoma. J Surg Oncol. 2020 Nov;122(6):1057–65.

110. Iravani A, Osman MM, Weppler AM, Wallace R, Galligan A, Lasocki A, et al. FDG PET/CT for tumoral and systemic immune response monitoring of advanced melanoma during first-line combination ipilimumab and nivolumab treatment. Eur J Nucl Med Mol Imaging. 2020 Nov;47(12):2776–86.

111. Mason R, Dearden HC, Nguyen B, Soon JA, Smith JL, Randhawa M, et al. Combined ipilimumab and nivolumab first-line and after BRAF-targeted therapy in advanced melanoma. Pigment Cell Melanoma Res. 2020 Mar;33(2):358–65.

112. Shi K, Zhu X, Liu Z, Sun N, Gu L, Wei Y, et al. Clinical characteristics of malignant melanoma in central China and predictors of metastasis. Oncol Lett. 2020 Feb;19(2):1452–64.

113. Huang K, Xu Y, Gabriel EM, Misra S, Chen Y, Bagaria SP. Comparative Analysis of Acral Melanoma in Chinese and Caucasian Patients. J Skin Cancer. 2020;2020:5169051.

114. Jang S, Poretta T, Bhagnani T, Harshaw Q, Burke M, Rao S. Real-World Recurrence Rates and Economic Burden in Patients with Resected Early-Stage Melanoma. Dermatol Ther. 2020 Oct;10(5):985–99.

115. Feigelson HS, Powers JD, Kumar M, Carroll NM, Pathy A, Ritzwoller DP. Melanoma incidence, recurrence, and mortality in an integrated healthcare system: A retrospective cohort study. Cancer Med. 2019 Aug;8(9):4508–16.

116. Schilling B, Martens A, Geukes Foppen MH, Gebhardt C, Hassel JC, Rozeman EA, et al. First-line therapy-stratified survival in BRAF-mutant melanoma: a retrospective multicenter analysis. Cancer Immunol Immunother CII. 2019 May;68(5):765–72.

117. Holmberg CJ, Alwan G, Ny L, Olofsson Bagge R, Katsarelias D. Surgery for gastrointestinal metastases of malignant melanoma - a retrospective exploratory study. World J Surg Oncol. 2019 Jul 12;17(1):123.

118. Márquez-Rodas I, Arance A, Berrocal A, Larios CL, Curto-García J, Campos-Tapias IX, et al. A retrospective chart review study describing metastatic melanoma patients profile and treatment patterns in Spain. Clin Transl Oncol Off Publ Fed Span Oncol Soc Natl Cancer Inst Mex. 2019 Dec;21(12):1754–62.

119. Liu FX, Ou W, Diede SJ, Whitman ED. Real-world experience with pembrolizumab in patients with advanced melanoma: A large retrospective observational study. Medicine (Baltimore). 2019 Jul;98(30):e16542.

120. Mohr P, Haferkamp S, Pinter A, Weishaupt C, Huber MA, Downey G, et al. Real-World Use of Talimogene Laherparepvec in German Patients with Stage IIIB to IVM1a Melanoma: A Retrospective Chart Review and Physician Survey. Adv Ther. 2019 Jan;36(1):101–17.

121. Mowery YM, Patel K, Chowdhary M, Rushing CN, Roy Choudhury K, Lowe JR, et al. Retrospective analysis of safety and efficacy of anti-PD-1 therapy and radiation therapy in advanced melanoma: A bi-institutional study. Radiother Oncol J Eur Soc Ther Radiol Oncol. 2019 Sep;138:114–20.

122. Czirbesz K, Gorka E, Balatoni T, Pánczél G, Melegh K, Kovács P, et al. Efficacy of Vemurafenib Treatment in 43 Metastatic Melanoma Patients with BRAF Mutation. Single-Institute Retrospective Analysis, Early Real-Life Survival Data. Pathol Oncol Res POR. 2019 Jan;25(1):45–50.

123. Jørgensen MG, Toyserkani NM, Thomsen JB, Sørensen JA. Prophylactic incisional negative pressure wound therapy shows promising results in prevention of wound complications following inguinal lymph node dissection for Melanoma: A retrospective case-control series. J Plast Reconstr Aesthetic Surg JPRAS. 2019 Jul;72(7):1178–83.

124. Cowey CL, Liu FX, Boyd M, Aguilar KM, Krepler C. Real-world treatment patterns and clinical outcomes among patients with advanced melanoma: A retrospective, community oncology-based cohort study (A STROBE-compliant article). Medicine (Baltimore). 2019 Jul;98(28):e16328.

125. Verver D, Poirier-Colame V, Tomasic G, Cherif-Rebai K, Grunhagen DJ, Verhoef C, et al. Upregulation of intratumoral HLA class I and peritumoral Mx1 in ulcerated melanomas. Oncoimmunology. 2019;8(11):e1660121.

126. Liu X, Zhang Q, Fan C, Tian J, Liu X, Li G. GDF11 upregulation independently predicts shorter overall-survival of uveal melanoma. PloS One. 2019;14(3):e0214073.

127. Gartrell RD, Marks DK, Rizk EM, Bogardus M, Gérard CL, Barker LW, et al. Validation of Melanoma Immune Profile (MIP), a Prognostic Immune Gene Prediction Score for Stage II-III Melanoma. Clin Cancer Res Off J Am Assoc Cancer Res. 2019 Apr 15;25(8):2494–502.

128. Ferguson SD, Bindal S, Bassett RL, Haydu LE, McCutcheon IE, Heimberger AB, et al. Predictors of survival in metastatic melanoma patients with leptomeningeal disease (LMD). J Neurooncol. 2019 May;142(3):499–509.

129. Cimminiello C, Indini A, Di Guardo L, Prisciandaro M, Randon G, Tolomio E, et al. Pembrolizumab in the treatment of advanced/metastatic melanoma: a single-center institution experience. Melanoma Res. 2019 Jun;29(3):289–94.

130. Song Y, Tieniber AD, Gimotty PA, Mitchell TC, Amaravadi RK, Schuchter LM, et al. Survival Outcomes of Patients with Clinical Stage III Melanoma in the Era of Novel Systemic Therapies. Ann Surg Oncol. 2019 Dec;26(13):4621–30.

131. Arheden A, Skalenius J, Bjursten S, Stierner U, Ny L, Levin M, et al. Real-world data on PD-1 inhibitor therapy in metastatic melanoma. Acta Oncol Stockh Swed. 2019 Jul;58(7):962–6.

132. Polkowska M, Ekk-Cierniakowski P, Czepielewska E, Kozłowska-Wojciechowska M. Efficacy and safety of BRAF inhibitors and anti-CTLA4 antibody in melanoma patients-real-world data. Eur J Clin Pharmacol. 2019 Mar;75(3):329–34.

133. Amaral T, Tampouri I, Eigentler T, Keim U, Klumpp B, Heinrich V, et al. Immunotherapy plus surgery/radiosurgery is associated with favorable survival in patients with melanoma brain metastasis. Immunotherapy. 2019 Mar;11(4):297–309.

134. Nan Tie E, Na LH, Hicks RJ, Spillane J, Speakman D, Henderson MA, et al. The Prognosis and Natural History of In-Transit Melanoma Metastases at a High-Volume Centre. Ann Surg Oncol. 2019 Dec;26(13):4673–80.

135. Mahvi DA, Fairweather M, Yoon CH, Cho NL. Utility of Level III Axillary Node Dissection in Melanoma Patients with Palpable Axillary Lymph Node Disease. Ann Surg Oncol. 2019 Sep;26(9):2846–54.

136. Parakh S, Randhawa M, Nguyen B, Warburton L, Hussain MA, Cebon J, et al. Real-world efficacy and toxicity of combined nivolumab and ipilimumab in patients with metastatic melanoma. Asia Pac J Clin Oncol. 2019 Feb;15(1):26–30.

137. Martin-Algarra S, Hinshelwood R, Mesnage S, Cebon J, Ferrucci PF, Aglietta M, et al. Effectiveness of dabrafenib in the treatment of patients with BRAF V600-mutated metastatic melanoma in a Named Patient Program. Melanoma Res. 2019 Oct;29(5):527–32.

138. Kulkarni PM, Robinson EJ, Sarin Pradhan J, Gartrell-Corrado RD, Rohr BR, Trager MH, et al. Deep Learning Based on Standard H&E Images of Primary Melanoma Tumors Identifies Patients at Risk for Visceral Recurrence and Death. Clin Cancer Res Off J Am Assoc Cancer Res. 2020 Mar 1;26(5):1126–34.

139. Hamid O, Molinero L, Bolen CR, Sosman JA, Muñoz-Couselo E, Kluger HM, et al. Safety, Clinical Activity, and Biological Correlates of Response in Patients with Metastatic Melanoma: Results from a Phase I Trial of Atezolizumab. Clin Cancer Res Off J Am Assoc Cancer Res. 2019 Oct 15;25(20):6061–72.

140. Masoud SJ, Hu JB, Beasley GM, Stewart JH, Mosca PJ. Efficacy of Talimogene Laherparepvec (T-VEC) Therapy in Patients with In-Transit Melanoma Metastasis Decreases with Increasing Lesion Size. Ann Surg Oncol. 2019 Dec;26(13):4633–41.

141. Louveau B, Jouenne F, Reger de Moura C, Sadoux A, Baroudjian B, Delyon J, et al. Baseline Genomic Features in BRAFV600-Mutated Metastatic Melanoma Patients Treated with BRAF Inhibitor + MEK Inhibitor in Routine Care. Cancers. 2019 Aug 18;11(8):1203.

142. Castanares-Zapatero D, Verleye L, Devos C, Thiry N, Silversmit G, Van Damme N, et al. Survival of patients with unfavorable prognosis cutaneous melanoma with increased use of immunotherapy agents: a population-based study in Belgium. Int J Dermatol. 2024 Jan 31;

143. McQuade JL, Daniel CR, Hess KR, Mak C, Wang DY, Rai RR, et al. Association of body-mass index and outcomes in patients with metastatic melanoma treated with targeted therapy, immunotherapy, or chemotherapy: a retrospective, multicohort analysis. Lancet Oncol. 2018 Mar;19(3):310–22.

144. Lattanzi M, Han J, Moran U, Utter K, Tchack J, Sabado RL, et al. Adjuvant NY-ESO-1 vaccine immunotherapy in high-risk resected melanoma: a retrospective cohort analysis. J Immunother Cancer. 2018 May 18;6(1):38.

145. Ma Q, Shilkrut M, Zhao Z, Li M, Batty N, Barber B. Autoimmune comorbidities in patients with metastatic melanoma: a retrospective analysis of us claims data. BMC Cancer. 2018 Feb 6;18(1):145.

146. Sam D, Gresham G, Abdel-Rahman O, Cheung WY. Generalizability of clinical trials of advanced melanoma in the real-world, population-based setting. Med Oncol Northwood Lond Engl. 2018 Jun 18;35(7):110.

147. De Caluwé A, Termote K, Van Gestel D, Van Limbergen E. Dose-response in choroidal melanoma. Radiother Oncol J Eur Soc Ther Radiol Oncol. 2018 Jun;127(3):374–8.

148. Tucker MA, Elder DE, Curry M, Fraser MC, Pichler V, Zametkin D, et al. Risks of Melanoma and Other Cancers in Melanoma-Prone Families over 4 Decades. J Invest Dermatol. 2018 Jul;138(7):1620–6.

149. Rachidi S, Wallace K, Li H, Lautenschlaeger T, Li Z. Postdiagnosis aspirin use and overall survival in patients with melanoma. J Am Acad Dermatol. 2018 May;78(5):949-956.e1.

150. Nakamura Y, Fujisawa Y, Tanaka R, Maruyama H, Ishitsuka Y, Okiyama N, et al. Use of immune checkpoint inhibitors prolonged overall survival in a Japanese population of advanced malignant melanoma patients: Retrospective single institutional study. J Dermatol. 2018 Nov;45(11):1337–9.

151. Okada N, Kawazoe H, Takechi K, Matsudate Y, Utsunomiya R, Zamami Y, et al. Association Between Immune-Related Adverse Events and Clinical Efficacy in Patients with Melanoma Treated With Nivolumab: A Multicenter Retrospective Study. Clin Ther. 2019 Jan;41(1):59–67.

152. Cowey CL, Liu FX, Black-Shinn J, Stevinson K, Boyd M, Frytak JR, et al. Pembrolizumab Utilization and Outcomes for Advanced Melanoma in US Community Oncology Practices. J Immunother Hagerstown Md 1997. 2018;41(2):86–95.

153. Hauschild A, Larkin J, Ribas A, Dréno B, Flaherty KT, Ascierto PA, et al. Modeled Prognostic Subgroups for Survival and Treatment Outcomes in BRAF V600-Mutated Metastatic Melanoma: Pooled Analysis of 4 Randomized Clinical Trials. JAMA Oncol. 2018 Oct 1;4(10):1382–8.

154. Glitza IC, Rohlfs M, Guha-Thakurta N, Bassett RL, Bernatchez C, Diab A, et al. Retrospective review of metastatic melanoma patients with leptomeningeal disease treated with intrathecal interleukin-2. ESMO Open. 2018;3(1):e000283.

155. Ibrahim T, Mateus C, Baz M, Robert C. Older melanoma patients aged 75 and above retain responsiveness to anti-PD1 therapy: results of a retrospective single-institution cohort study. Cancer Immunol Immunother CII. 2018 Oct;67(10):1571–8.

156. Kähler KC, Eigentler TK, Gesierich A, Heinzerling L, Loquai C, Meier F, et al. Ipilimumab in metastatic melanoma patients with pre-existing autoimmune disorders. Cancer Immunol Immunother CII. 2018 May;67(5):825–34.

157. Haag GM, Zoernig I, Hassel JC, Halama N, Dick J, Lang N, et al. Phase II trial of ipilimumab in melanoma patients with preexisting humoural immune response to NY-ESO-1. Eur J Cancer Oxf Engl 1990. 2018 Feb;90:122–9.

158. Perier-Muzet M, Gatt E, Péron J, Falandry C, Amini-Adlé M, Thomas L, et al. Association of Immunotherapy With Overall Survival in Elderly Patients With Melanoma. JAMA Dermatol. 2018 Jan 1;154(1):82–7.

159. Rosner S, Kwong E, Shoushtari AN, Friedman CF, Betof AS, Brady MS, et al. Peripheral blood clinical laboratory variables associated with outcomes following combination nivolumab and ipilimumab immunotherapy in melanoma. Cancer Med. 2018 Mar;7(3):690–7.

160. Hanna TP, Nguyen P, Baetz T, Booth CM, Eisenhauer E. A Population-based Study of Survival Impact of New Targeted and Immune-based Therapies for Metastatic or Unresectable Melanoma. Clin Oncol R Coll Radiol G B. 2018 Oct;30(10):609–17.

161. Afzal MZ, Mercado RR, Shirai K. Efficacy of metformin in combination with immune checkpoint inhibitors (anti-PD-1/anti-CTLA-4) in metastatic malignant melanoma. J Immunother Cancer. 2018 Jul 2;6(1):64.

162. Lee RJ, Gremel G, Marshall A, Myers KA, Fisher N, Dunn JA, et al. Circulating tumor DNA predicts survival in patients with resected high-risk stage II/III melanoma. Ann Oncol Off J Eur Soc Med Oncol. 2018 Feb 1;29(2):490–6.

163. Lo SN, Scolyer RA, Thompson JF. Long-Term Survival of Patients with Thin (T1) Cutaneous Melanomas: A Breslow Thickness Cut Point of 0.8 mm Separates Higher-Risk and Lower-Risk Tumors. Ann Surg Oncol. 2018 Apr;25(4):894–902.

164. Verver D, van Klaveren D, Franke V, van Akkooi ACJ, Rutkowski P, Keilholz U, et al. Development and validation of a nomogram to predict recurrence and melanoma-specific mortality in patients with negative sentinel lymph nodes. Br J Surg. 2019 Feb;106(3):217–25.

165. Rossi CR, Mocellin S, Campana LG, Borgognoni L, Sestini S, Giudice G, et al. Prediction of Non-sentinel Node Status in Patients with Melanoma and Positive Sentinel Node Biopsy: An Italian Melanoma Intergroup (IMI) Study. Ann Surg Oncol. 2018 Jan;25(1):271–9.

166. Sinnamon AJ, Song Y, Sharon CE, Yang YX, Elder DE, Zhang PJ, et al. Prediction of Residual Nodal Disease at Completion Dissection Following Positive Sentinel Lymph Node Biopsy for Melanoma. Ann Surg Oncol. 2018 Nov;25(12):3469–75.

167. Teterycz P, Jagodzińska-Mucha P, Cybulska-Stopa B, Mariuk-Jarema A, Kozak K, Koseła-Paterczyk H, et al. High baseline neutrophil-to-lymphocyte ratio predicts worse outcome in patients with metastatic BRAF-positive melanoma treated with BRAF and MEK inhibitors. Melanoma Res. 2018 Oct;28(5):435–41.

168. Dillman RO, Cornforth AN, Nistor GI, McClay EF, Amatruda TT, Depriest C. Randomized phase II trial of autologous dendritic cell vaccines versus autologous tumor cell vaccines in metastatic melanoma: 5-year follow up and additional analyses. J Immunother Cancer. 2018 Mar 6;6(1):19.

169. Kirchberger MC, Moreira A, Erdmann M, Schuler G, Heinzerling L. Real world experience in low-dose ipilimumab in combination with PD-1 blockade in advanced melanoma patients. Oncotarget. 2018 Jun 22;9(48):28903–9.

170. Mavor ME, Richardson H, Miao Q, Asai Y, Hanna TP. Disparities in diagnosis of advanced melanoma: a population-based cohort study. CMAJ Open. 2018;6(4):E502–12.

171. Lu W, Burton L, Larkin J, Chapman PB, Ascierto PA, Ribas A, et al. Elevated Levels of BRAFV600 Mutant Circulating Tumor DNA and Circulating Hepatocyte Growth Factor Are Associated With Poor Prognosis in Patients With Metastatic Melanoma. JCO Precis Oncol. 2018 Nov;2:1–17.

172. Castaneda CA, Torres-Cabala C, Castillo M, Villegas V, Casavilca S, Cano L, et al. Tumor infiltrating lymphocytes in acral lentiginous melanoma: a study of a large cohort of cases from Latin America. Clin Transl Oncol Off Publ Fed Span Oncol Soc Natl Cancer Inst Mex. 2017 Dec;19(12):1478–88.

173. Samlowski WE, Moon J, Witter M, Atkins MB, Kirkwood JM, Othus M, et al. High frequency of brain metastases after adjuvant therapy for high-risk melanoma. Cancer Med. 2017 Nov;6(11):2576–85.

174. Fujisawa Y, Yoshino K, Otsuka A, Funakoshi T, Fujimura T, Yamamoto Y, et al. Fluctuations in routine blood count might signal severe immune-related adverse events in melanoma patients treated with nivolumab. J Dermatol Sci. 2017 Nov;88(2):225–31.

175. Long GV, Weber JS, Larkin J, Atkinson V, Grob JJ, Schadendorf D, et al. Nivolumab for Patients With Advanced Melanoma Treated Beyond Progression: Analysis of 2 Phase 3 Clinical Trials. JAMA Oncol. 2017 Nov 1;3(11):1511–9.

176. Patel KR, Shoukat S, Oliver DE, Chowdhary M, Rizzo M, Lawson DH, et al. Ipilimumab and Stereotactic Radiosurgery Versus Stereotactic Radiosurgery Alone for Newly Diagnosed Melanoma Brain Metastases. Am J Clin Oncol. 2017 Oct;40(5):444–50.

177. Zimmer L, Apuri S, Eroglu Z, Kottschade LA, Forschner A, Gutzmer R, et al. Ipilimumab alone or in combination with nivolumab after progression on anti-PD-1 therapy in advanced melanoma. Eur J Cancer Oxf Engl 1990. 2017 Apr;75:47–55.

178. Meerveld-Eggink A, Rozeman EA, Lalezari F, van Thienen JV, Haanen JB a. G, Blank CU. Short-term CTLA-4 blockade directly followed by PD-1 blockade in advanced melanoma patients: a single-center experience. Ann Oncol Off J Eur Soc Med Oncol. 2017 Apr 1;28(4):862–7.

179. Weber JS, Hodi FS, Wolchok JD, Topalian SL, Schadendorf D, Larkin J, et al. Safety Profile of Nivolumab Monotherapy: A Pooled Analysis of Patients With Advanced Melanoma. J Clin Oncol Off J Am Soc Clin Oncol. 2017 Mar;35(7):785–92.

180. Leontovich AA, Dronca RS, Nevala WK, Thompson MA, Kottschade LA, Ivanov LV, et al. Effect of the lymphocyte-to-monocyte ratio on the clinical outcome of chemotherapy administration in advanced melanoma patients. Melanoma Res. 2017 Feb;27(1):32–42.

181. Smit AK, Espinoza D, Newson AJ, Morton RL, Fenton G, Freeman L, et al. A Pilot Randomized Controlled Trial of the Feasibility, Acceptability, and Impact of Giving Information on Personalized Genomic Risk of Melanoma to the Public. Cancer Epidemiol Biomark Prev Publ Am Assoc Cancer Res Cosponsored Am Soc Prev Oncol. 2017 Feb;26(2):212–21.

182. Timerman D, McEnery-Stonelake M, Joyce CJ, Nambudiri VE, Hodi FS, Claus EB, et al. Vitamin D deficiency is associated with a worse prognosis in metastatic melanoma. Oncotarget. 2017 Jan 24;8(4):6873–82.

183. O’Shea SJ, Rogers Z, Warburton F, Ramirez AJ, Newton-Bishop JA, Forbes LJL. Which symptoms are linked to a delayed presentation among melanoma patients? A retrospective study. BMC Cancer. 2017 Jan 3;17(1):5.

184. Mosquera C, Vora HS, Vohra N, Fitzgerald TL. Population-Based Analysis of Completion Lymphadenectomy in Intermediate-Thickness Melanoma. Ann Surg Oncol. 2017 Jan;24(1):127–34.

185. Andtbacka RHI, Agarwala SS, Ollila DW, Hallmeyer S, Milhem M, Amatruda T, et al. Cutaneous head and neck melanoma in OPTiM, a randomized phase 3 trial of talimogene laherparepvec versus granulocyte-macrophage colony-stimulating factor for the treatment of unresected stage IIIB/IIIC/IV melanoma. Head Neck. 2016 Dec;38(12):1752–8.

186. Algazi AP, Tsai KK, Shoushtari AN, Munhoz RR, Eroglu Z, Piulats JM, et al. Clinical outcomes in metastatic uveal melanoma treated with PD-1 and PD-L1 antibodies. Cancer. 2016 Nov 15;122(21):3344–53.

187. Koskivuo I, Kemppainen J, Giordano S, Seppänen M, Veräjänkorva E, Vihinen P, et al. Whole body PET/CT in the follow-up of asymptomatic patients with stage IIB-IIIB cutaneous melanoma. Acta Oncol Stockh Swed. 2016 Nov;55(11):1355–9.

188. Srisuttiyakorn C, Bulloch K, Rodic N, Bosenberg M, Ariyan S, Narayan D, et al. Intratumoral multinucleated giant cells are not a prognostic pathologic feature in cutaneous melanoma. J Cutan Pathol. 2016 Oct;43(10):821–9.

189. Walter SD, Chao DL, Feuer W, Schiffman J, Char DH, Harbour JW. Prognostic Implications of Tumor Diameter in Association With Gene Expression Profile for Uveal Melanoma. JAMA Ophthalmol. 2016 Jul 1;134(7):734–40.

190. Bol KF, Aarntzen EHJG, Hout FEMI ’t, Schreibelt G, Creemers JHA, Lesterhuis WJ, et al. Favorable overall survival in stage III melanoma patients after adjuvant dendritic cell vaccination. Oncoimmunology. 2016;5(1):e1057673.

191. Meani RE, Pan Y, McLean C, Haydon A, Leung M, Kelly JW. The Victorian Melanoma Service: A 20-year review of an Australian multidisciplinary cancer service. Australas J Dermatol. 2016 Aug;57(3):235–7.

192. Grignol VP, Smith AD, Shlapak D, Zhang X, Del Campo SM, Carson WE. Increased visceral to subcutaneous fat ratio is associated with decreased overall survival in patients with metastatic melanoma receiving anti-angiogenic therapy. Surg Oncol. 2015 Dec;24(4):353–8.

193. Ramirez AG, Wages NA, Hu Y, Smolkin ME, Slingluff CL. Defining the effects of age and gender on immune response and outcomes to melanoma vaccination: a retrospective analysis of a single-institution clinical trials’ experience. Cancer Immunol Immunother CII. 2015 Dec;64(12):1531–9.

194. Coupland SE, Kalirai H, Ho V, Thornton S, Damato BE, Heimann H. Concordant chromosome 3 results in paired choroidal melanoma biopsies and subsequent tumour resection specimens. Br J Ophthalmol. 2015 Oct;99(10):1444–50.

195. Smith AD, Gray MR, del Campo SM, Shlapak D, Ganeshan B, Zhang X, et al. Predicting Overall Survival in Patients With Metastatic Melanoma on Antiangiogenic Therapy and RECIST Stable Disease on Initial Posttherapy Images Using CT Texture Analysis. AJR Am J Roentgenol. 2015 Sep;205(3):W283-293.

196. Larkin J, Lao CD, Urba WJ, McDermott DF, Horak C, Jiang J, et al. Efficacy and Safety of Nivolumab in Patients With BRAF V600 Mutant and BRAF Wild-Type Advanced Melanoma: A Pooled Analysis of 4 Clinical Trials. JAMA Oncol. 2015 Jul;1(4):433–40.

197. Harding JJ, Catalanotti F, Munhoz RR, Cheng DT, Yaqubie A, Kelly N, et al. A Retrospective Evaluation of Vemurafenib as Treatment for BRAF-Mutant Melanoma Brain Metastases. The Oncologist. 2015 Jul;20(7):789–97.

198. Takiar V, Voong KR, Gombos DS, Mourtada F, Rechner LA, Lawyer AA, et al. A choice of radionuclide: Comparative outcomes and toxicity of ruthenium-106 and iodine-125 in the definitive treatment of uveal melanoma. Pract Radiat Oncol. 2015;5(3):e169–76.

199. Lee JL, Haydu LE, Thompson JF, Morton RL. The impact of socioeconomic status on melanoma clinical trial participation: an observational cohort study from Australia. Melanoma Manag. 2014 Nov;1(2):95–103.

200. Lee CI, Menzies AM, Haydu LE, Azer M, Clements A, Kefford RF, et al. Features and management of pyrexia with combined dabrafenib and trametinib in metastatic melanoma. Melanoma Res. 2014 Oct;24(5):468–74.

201. Choe CH, McArthur GA, Caro I, Kempen JH, Amaravadi RK. Ocular toxicity in BRAF mutant cutaneous melanoma patients treated with vemurafenib. Am J Ophthalmol. 2014 Oct;158(4):831-837.e2.

202. van der Ploeg APT, Haydu LE, Spillane AJ, Scolyer RA, Quinn MJ, Saw RPM, et al. Melanoma patients with an unknown primary tumor site have a better outcome than those with a known primary following therapeutic lymph node dissection for macroscopic (clinically palpable) nodal disease. Ann Surg Oncol. 2014 Sep;21(9):3108–16.

203. Egger ME, Brown RE, Roach BA, Quillo AR, Martin RCG, Scoggins CR, et al. Addition of an iliac/obturator lymph node dissection does not improve nodal recurrence or survival in melanoma. J Am Coll Surg. 2014 Jul;219(1):101–8.

204. Kan S, Konishi E, Arita T, Ikemoto C, Takenaka H, Yanagisawa A, et al. Podoplanin expression in cancer-associated fibroblasts predicts aggressive behavior in melanoma. J Cutan Pathol. 2014 Jul;41(7):561–7.

205. Bedikian AY, Garbe C, Conry R, Lebbe C, Grob JJ, Genasense Melanoma Study Group. Dacarbazine with or without oblimersen (a Bcl-2 antisense oligonucleotide) in chemotherapy-naive patients with advanced melanoma and low-normal serum lactate dehydrogenase: “The AGENDA trial.” Melanoma Res. 2014 Jun;24(3):237–43.

206. Gupta A, Love S, Schuh A, Shanyinde M, Larkin JM, Plummer R, et al. DOC-MEK: a double-blind randomized phase II trial of docetaxel with or without selumetinib in wild-type BRAF advanced melanoma. Ann Oncol Off J Eur Soc Med Oncol. 2014 May;25(5):968–74.

207. Egger ME, Bower MR, Czyszczon IA, Farghaly H, Noyes RD, Reintgen DS, et al. Comparison of sentinel lymph node micrometastatic tumor burden measurements in melanoma. J Am Coll Surg. 2014 Apr;218(4):519–28.

208. Lau DK, Andrews MC, Turner N, Azad AA, Davis ID, Cebon JS. A single-centre experience of patients with metastatic melanoma enrolled in a dabrafenib named patient programme. Melanoma Res. 2014 Apr;24(2):144–9.

209. Gómez-Olivas JD, Campos-Rodriguez F, Nagore E, Martorell A, García-Rio F, Cubillos C, et al. Role of Sleep Apnea and Long-Term CPAP Treatment in the Prognosis of Patients With Melanoma: A Prospective Multicenter Study of 443 Patients. Chest. 2023 Dec;164(6):1551–9.

210. Atkins MB, Lee SJ, Chmielowski B, Tarhini AA, Cohen GI, Truong TG, et al. Combination Dabrafenib and Trametinib Versus Combination Nivolumab and Ipilimumab for Patients With Advanced BRAF-Mutant Melanoma: The DREAMseq Trial-ECOG-ACRIN EA6134. J Clin Oncol Off J Am Soc Clin Oncol. 2023 Jan 10;41(2):186–97.

211. Ascierto PA, Mandalà M, Ferrucci PF, Guidoboni M, Rutkowski P, Ferraresi V, et al. Sequencing of Ipilimumab Plus Nivolumab and Encorafenib Plus Binimetinib for Untreated BRAF-Mutated Metastatic Melanoma (SECOMBIT): A Randomized, Three-Arm, Open-Label Phase II Trial. J Clin Oncol Off J Am Soc Clin Oncol. 2023 Jan 10;41(2):212–21.

212. Carpenter EL, Van Decar S, Adams AM, O’Shea AE, McCarthy P, Chick RC, et al. Prospective, randomized, double-blind phase 2B trial of the TLPO and TLPLDC vaccines to prevent recurrence of resected stage III/IV melanoma: a prespecified 36-month analysis. J Immunother Cancer. 2023 Aug;11(8):e006665.

213. Marchetti MA, Cowen EA, Kurtansky NR, Weber J, Dauscher M, DeFazio J, et al. Prospective validation of dermoscopy-based open-source artificial intelligence for melanoma diagnosis (PROVE-AI study). NPJ Digit Med. 2023 Jul 12;6(1):127.

214. Berking C, Livingstone E, Debus D, Loquai C, Weichenthal M, Leiter U, et al. COMBI-r: A Prospective, Non-Interventional Study of Dabrafenib Plus Trametinib in Unselected Patients with Unresectable or Metastatic BRAF V600-Mutant Melanoma. Cancers. 2023 Sep 6;15(18):4436.

215. Oldan JD, Giglio BC, Smith E, Zhao W, Bouchard DM, Ivanovic M, et al. Increased tryptophan, but not increased glucose metabolism, predict resistance of pembrolizumab in stage III/IV melanoma. Oncoimmunology. 2023;12(1):2204753.

216. Brunsgaard EK, Bowles TL, Asare EA, Grossmann K, Boucher KM, Grossmann A, et al. Feasibility of personalized circulating tumor DNA detection in stage II and III melanoma. Melanoma Res. 2023 Jun 1;33(3):184–91.

217. Meyer S, Buser L, Haferkamp S, Berneburg M, Maisch T, Klinkhammer-Schalke M, et al. Identification of high-risk patients with a seven-biomarker prognostic signature for adjuvant treatment trial recruitment in American Joint Committee on Cancer v8 stage I-IIA cutaneous melanoma. Eur J Cancer Oxf Engl 1990. 2023 Mar;182:77–86.

218. Bartula I, Tran AD, Nowak AK, Ahmed T, Morton RL, Burmeister BH, et al. Longitudinal trajectory of quality of life for patients with melanoma brain metastases: A secondary analysis from a whole brain radiotherapy randomized clinical trial. Clin Transl Radiat Oncol. 2023 Jul;41:100597.

219. Cerminara SE, Cheng P, Kostner L, Huber S, Kunz M, Maul JT, et al. Diagnostic performance of augmented intelligence with 2D and 3D total body photography and convolutional neural networks in a high-risk population for melanoma under real-world conditions: A new era of skin cancer screening? Eur J Cancer Oxf Engl 1990. 2023 Sep;190:112954.

220. Gente K, Diekmann L, Daniello L, Will J, Feisst M, Olsavszky V, et al. Sex and anti-inflammatory treatment affect outcome of melanoma and non-small cell lung cancer patients with rheumatic immune-related adverse events. J Immunother Cancer. 2023 Sep;11(9):e007557.

221. Holmberg CJ, Mikiver R, Isaksson K, Ingvar C, Moncrieff M, Nielsen K, et al. Prognostic Significance of Sentinel Lymph Node Status in Thick Primary Melanomas (> 4 mm). Ann Surg Oncol. 2023 Dec;30(13):8026–33.

222. Pavoine M, Thuillier P, Karakatsanis N, Legoupil D, Amrane K, Floch R, et al. Clinical application of a population-based input function (PBIF) for a shortened dynamic whole-body FDG-PET/CT protocol in patients with metastatic melanoma treated by immunotherapy. EJNMMI Phys. 2023 Dec 8;10(1):79.

223. Del Vecchio M, Chiarion Sileni V, Quaglino P, Rinaldi G, Minisini A, Troiani T, et al. The Pattern of Progression to First-Line Treatment with Dabrafenib and Trametinib in Patients with Unresectable or Metastatic, BRAF-Mutated, Cutaneous Melanoma: Results of the Observational T-WIN Study. Cancers. 2023 Mar 26;15(7):1980.

224. Sadrolashrafi K, Samlowski W. Retreatment of Patients With Metastatic Cutaneous Melanoma Who Relapse After Elective Checkpoint Inhibitor Discontinuation After a Complete Remission. The Oncologist. 2023 May 8;28(5):e270–5.

225. Multicenter Selective Lymphadenectomy Trials Study Group, Crystal JS, Thompson JF, Hyngstrom J, Caracò C, Zager JS, et al. Therapeutic Value of Sentinel Lymph Node Biopsy in Patients With Melanoma: A Randomized Clinical Trial. JAMA Surg. 2022 Sep 1;157(9):835–42.

226. Pellacani G, Farnetani F, Ciardo S, Chester J, Kaleci S, Mazzoni L, et al. Effect of Reflectance Confocal Microscopy for Suspect Lesions on Diagnostic Accuracy in Melanoma: A Randomized Clinical Trial. JAMA Dermatol. 2022 Jul 1;158(7):754–61.

227. Jørgensen MG, Chakera AH, Hölmich LR, Drejøe JB, Andersen PCL, Khorasani H, et al. Can prophylactic incisional negative pressure wound therapy Reduce Wound Complications After Inguinal Lymph Node Dissection for Melanoma? Results from a Randomized Controlled Trial. JPRAS Open. 2022 Dec;34:134–43.

228. Naeser Y, Helgadottir H, Hansson J, Ingvar C, Elander NO, Flygare P, et al. Quality of Life in the First Year of Follow-Up in a Randomized Multicenter Trial Assessing the Role of Imaging after Radical Surgery of Stage IIB-C and III Cutaneous Melanoma (TRIM Study). Cancers. 2022 Feb 18;14(4):1040.

229. Aung TN, Shafi S, Wilmott JS, Nourmohammadi S, Vathiotis I, Gavrielatou N, et al. Objective assessment of tumor infiltrating lymphocytes as a prognostic marker in melanoma using machine learning algorithms. EBioMedicine. 2022 Aug;82:104143.

230. Friedman CF, Spencer C, Cabanski CR, Panageas KS, Wells DK, Ribas A, et al. Ipilimumab alone or in combination with nivolumab in patients with advanced melanoma who have progressed or relapsed on PD-1 blockade: clinical outcomes and translational biomarker analyses. J Immunother Cancer. 2022 Jan;10(1):e003853.

231. Jahn AS, Navarini AA, Cerminara SE, Kostner L, Huber SM, Kunz M, et al. Over-Detection of Melanoma-Suspect Lesions by a CE-Certified Smartphone App: Performance in Comparison to Dermatologists, 2D and 3D Convolutional Neural Networks in a Prospective Data Set of 1204 Pigmented Skin Lesions Involving Patients’ Perception. Cancers. 2022 Aug 7;14(15):3829.

232. Sanjida S, Betz-Stablein B, Atkinson V, Janda M, Barsoum R, Edwards HA, et al. In-Depth Characterisation of Real-World Advanced Melanoma Patients Receiving Immunotherapies and/or Targeted Therapies: A Case Series. Cancers. 2022 Jun 4;14(11):2801.

233. Zhou L, Wu X, Chi Z, Si L, Sheng X, Kong Y, et al. Safety, activity, and pharmacokinetics of camrelizumab in advanced Asian melanoma patients: a phase I study. BMC Cancer. 2022 May 20;22(1):565.

234. Yan J, Wu X, Yu J, Kong Y, Cang S. An Immune-Related Gene Pair Index Predicts Clinical Response and Survival Outcome of Immune Checkpoint Inhibitors in Melanoma. Front Immunol. 2022;13:839901.

235. Aamdal E, Skovlund E, Jacobsen KD, Straume O, Kersten C, Herlofsen O, et al. Health-related quality of life in patients with advanced melanoma treated with ipilimumab: prognostic implications and changes during treatment. ESMO Open. 2022 Oct;7(5):100588.

236. Voglis S, Schaller V, Müller T, Gönel M, Winklhofer S, Mangana J, et al. Maximal surgical tumour load reduction in immune-checkpoint inhibitor naïve patients with melanoma brain metastases correlates with prolonged survival. Eur J Cancer Oxf Engl 1990. 2022 Nov;175:158–68.

237. Aldenhoven L, Frotscher C, Körver-Steeman R, Martens MH, Kuburic D, Janssen A, et al. Sentinel lymph node mapping with superparamagnetic iron oxide for melanoma: a pilot study in healthy participants to establish an optimal MRI workflow protocol. BMC Cancer. 2022 Oct 14;22(1):1062.

238. Jakub JW, Weaver AL, Meves A. Association of tumor molecular factors with in-transit metastasis in primary cutaneous melanoma. Int J Dermatol. 2022 Sep;61(9):1117–23.

239. Holmberg CJ, Ny L, Hieken TJ, Block MS, Carr MJ, Sondak VK, et al. The efficacy of immune checkpoint blockade for melanoma in-transit with or without nodal metastases - A multicenter cohort study. Eur J Cancer Oxf Engl 1990. 2022 Jul;169:210–22.

240. Wiens L, Schäffeler N, Eigentler T, Garbe C, Forschner A. Psychological Distress of Metastatic Melanoma Patients during Treatment with Immune Checkpoint Inhibitors: Results of a Prospective Study. Cancers. 2021 May 27;13(11):2642.

241. Lan S, Cui Z, Yin Q, Liu Z, Liang L, He H, et al. Prospective study of clinical characteristics of melanoma patients with retinopathy caused by a high-dose interferon α-2b. Melanoma Res. 2021 Dec 1;31(6):550–4.

242. MacLellan AN, Price EL, Publicover-Brouwer P, Matheson K, Ly TY, Pasternak S, et al. The use of noninvasive imaging techniques in the diagnosis of melanoma: a prospective diagnostic accuracy study. J Am Acad Dermatol. 2021 Aug;85(2):353–9.

243. LoRusso PM, Sekulic A, Sosman JA, Liang WS, Carpten J, Craig DW, et al. Identifying treatment options for BRAFV600 wild-type metastatic melanoma: A SU2C/MRA genomics-enabled clinical trial. PloS One. 2021;16(4):e0248097.

244. Herts KL, Jorge-Miller A, Beran TM, McCannel TA, Wiley JF, Stanton AL. A prospective study of cancer-related benefit finding in uveal melanoma patients. J Behav Med. 2021 Feb;44(1):131–7.

245. Guadagni S, Zoras O, Fiorentini G, Masedu F, Lasithiotakis K, Sarti D, et al. A Prospective Study of Intraarterial Infusion Chemotherapy in Advanced Wild-Type BRAF Melanoma Patients. J Surg Res. 2021 Dec;268:737–47.

246. Blankenstein SA, Rohaan MW, Klop WMC, van der Hiel B, van de Wiel BA, Lahaye MJ, et al. Neoadjuvant Cytoreductive Treatment With BRAF/MEK Inhibition of Prior Unresectable Regionally Advanced Melanoma to Allow Complete Surgical Resection, REDUCTOR: A Prospective, Single-arm, Open-label Phase II Trial. Ann Surg. 2021 Aug 1;274(2):383–9.

247. Bakshi A, Yan M, Riaz M, Polekhina G, Orchard SG, Tiller J, et al. Genomic Risk Score for Melanoma in a Prospective Study of Older Individuals. J Natl Cancer Inst. 2021 Oct 1;113(10):1379–85.

248. Gollrad J, Rabsahl C, Riechardt AI, Heufelder J, Stroux A, Goerling U, et al. Quality of life and treatment-related burden during ocular proton therapy: a prospective trial of 131 patients with uveal melanoma. Radiat Oncol Lond Engl. 2021 Sep 8;16(1):174.

249. de Meza MM, Ismail RK, Rauwerdink D, van Not OJ, van Breeschoten J, Blokx WAM, et al. Adjuvant treatment for melanoma in clinical practice - Trial versus reality. Eur J Cancer Oxf Engl 1990. 2021 Nov;158:234–45.

250. Aamdal E, Jacobsen KD, Straume O, Kersten C, Herlofsen O, Karlsen J, et al. Ipilimumab in a real-world population: A prospective Phase IV trial with long-term follow-up. Int J Cancer. 2022 Jan 1;150(1):100–11.

251. Yamazaki N, Takenouchi T, Nakamura Y, Takahashi A, Namikawa K, Kitano S, et al. Prospective observational study of the efficacy of nivolumab in Japanese patients with advanced melanoma (CREATIVE study). Jpn J Clin Oncol. 2021 Aug 1;51(8):1232–41.

252. Watts CG, McLoughlin K, Goumas C, van Kemenade CH, Aitken JF, Soyer HP, et al. Association Between Melanoma Detected During Routine Skin Checks and Mortality. JAMA Dermatol. 2021 Dec 1;157(12):1425–36.

253. Gómez Olivas JD, Campos-Rodriguez F, Nagore E, Hernández L, Cabriada V, Abad J, et al. Sleep Duration and Cutaneous Melanoma Aggressiveness. A Prospective Observational Study in 443 Patients. Arch Bronconeumol. 2021 Dec;57(12):776–8.

254. Storkus WJ, Maurer D, Lin Y, Ding F, Bose A, Lowe D, et al. Dendritic cell vaccines targeting tumor blood vessel antigens in combination with dasatinib induce therapeutic immune responses in patients with checkpoint-refractory advanced melanoma. J Immunother Cancer. 2021 Nov;9(11):e003675.

255. Reijers ILM, Rawson RV, Colebatch AJ, Rozeman EA, Menzies AM, van Akkooi ACJ, et al. Representativeness of the Index Lymph Node for Total Nodal Basin in Pathologic Response Assessment After Neoadjuvant Checkpoint Inhibitor Therapy in Patients With Stage III Melanoma. JAMA Surg. 2022 Apr 1;157(4):335–42.

256. Becquart O, Oriano B, Dalle S, Mortier L, Leccia MT, Dutriaux C, et al. Tolerance and Effectiveness of Targeted Therapies in Aged Patients with Metastatic Melanoma. Cancers. 2021 Jun 18;13(12):3042.

257. Dalle S, Mortier L, Corrie P, Lotem M, Board R, Arance AM, et al. Long-term real-world experience with ipilimumab and non-ipilimumab therapies in advanced melanoma: the IMAGE study. BMC Cancer. 2021 May 29;21(1):642.

258. Guitera P, Menzies SW, Coates E, Azzi A, Fernandez-Penas P, Lilleyman A, et al. Efficiency of Detecting New Primary Melanoma Among Individuals Treated in a High-risk Clinic for Skin Surveillance. JAMA Dermatol. 2021 May 1;157(5):521–30.

259. Saiag P, Robert C, Grob JJ, Mortier L, Dereure O, Lebbe C, et al. Efficacy, safety and factors associated with disease progression in patients with unresectable (stage III) or distant metastatic (stage IV) BRAF V600-mutant melanoma: An open label, non-randomized, phase IIIb study of trametinib in combination with dabrafenib. Eur J Cancer Oxf Engl 1990. 2021 Sep;154:57–65.

260. Gulati N, Celen A, Johannet P, Mehnert JM, Weber J, Krogsgaard M, et al. Preexisting immune-mediated inflammatory disease is associated with improved survival and increased toxicity in melanoma patients who receive immune checkpoint inhibitors. Cancer Med. 2021 Nov;10(21):7457–65.

261. Pokorny R, McPherson JP, Haaland B, Grossmann KF, Luckett C, Voorhies BN, et al. Real-world experience with elective discontinuation of PD-1 inhibitors at 1 year in patients with metastatic melanoma. J Immunother Cancer. 2021 Jan;9(1):e001781.

262. Tarhini AA, Toor K, Chan K, McDermott DF, Mohr P, Larkin J, et al. A matching-adjusted indirect comparison of combination nivolumab plus ipilimumab with BRAF plus MEK inhibitors for the treatment of BRAF-mutant advanced melanoma☆. ESMO Open. 2021 Apr;6(2):100050.

263. Patel N, Allen M, Arianpour K, Keidan R. The utility of ICG fluorescence for sentinel lymph node identification in head and neck melanoma. Am J Otolaryngol. 2021;42(5):103147.

264. Bulgarelli J, Piccinini C, Petracci E, Pancisi E, Granato AM, de Rosa F, et al. Radiotherapy and High-Dose Interleukin-2: Clinical and Immunological Results of a Proof of Principle Study in Metastatic Melanoma and Renal Cell Carcinoma. Front Immunol. 2021;12:778459.

265. Bai X, Dai J, Li C, Cui C, Mao L, Wei X, et al. Risk Models for Advanced Melanoma Patients Under Anti-PD-1 Monotherapy-Ad hoc Analyses of Pooled Data From Two Clinical Trials. Front Oncol. 2021;11:639085.

266. Kobayashi T, Iwama S, Yasuda Y, Okada N, Okuji T, Ito M, et al. Pituitary dysfunction induced by immune checkpoint inhibitors is associated with better overall survival in both malignant melanoma and non-small cell lung carcinoma: a prospective study. J Immunother Cancer. 2020 Jul;8(2):e000779.

267. Nomura M, Oze I, Masuishi T, Yokota T, Satake H, Iwasawa S, et al. Multicenter prospective phase II trial of nivolumab in patients with unresectable or metastatic mucosal melanoma. Int J Clin Oncol. 2020 May;25(5):972–7.

268. Binkley E, Triozzi PL, Rybicki L, Achberger S, Aldrich W, Singh A. A prospective trial of adjuvant therapy for high-risk uveal melanoma: assessing 5-year survival outcomes. Br J Ophthalmol. 2020 Apr;104(4):524–8.

269. Postow MA, Knox SJ, Goldman DA, Elhanati Y, Mavinkurve V, Wong P, et al. A Prospective, Phase 1 Trial of Nivolumab, Ipilimumab, and Radiotherapy in Patients with Advanced Melanoma. Clin Cancer Res Off J Am Assoc Cancer Res. 2020 Jul 1;26(13):3193–201.

270. Boudewijns S, Bloemendal M, de Haas N, Westdorp H, Bol KF, Schreibelt G, et al. Autologous monocyte-derived DC vaccination combined with cisplatin in stage III and IV melanoma patients: a prospective, randomized phase 2 trial. Cancer Immunol Immunother CII. 2020 Mar;69(3):477–88.

271. de Carvalho CEB, Capuzzo R, Crovador C, Teixeira RJ, Laus AC, Carvalho AL, et al. Near Infrared (NIR) Fluorescence is Not a Substitute for Lymphoscintigraphy and Gamma Probe for Melanoma Sentinel Node Detection: Results from a Prospective Trial. Ann Surg Oncol. 2020 Aug;27(8):2906–12.

272. Dummer R, Brase JC, Garrett J, Campbell CD, Gasal E, Squires M, et al. Adjuvant dabrafenib plus trametinib versus placebo in patients with resected, BRAFV600-mutant, stage III melanoma (COMBI-AD): exploratory biomarker analyses from a randomised, phase 3 trial. Lancet Oncol. 2020 Mar;21(3):358–72.

273. Tang B, Chi Z, Chen Y, Liu X, Wu D, Chen J, et al. Safety, Efficacy, and Biomarker Analysis of Toripalimab in Previously Treated Advanced Melanoma: Results of the POLARIS-01 Multicenter Phase II Trial. Clin Cancer Res Off J Am Assoc Cancer Res. 2020 Aug 15;26(16):4250–9.

274. Ali A, Dumbrava M, Riddell K, Stewart N, Ward R, Ibrahim AK, et al. Correlation between initial tumour volume and treatment duration on Dabrafenib: observation study of subjects with BRAF mutant melanoma on the BRF112680 trial. BMC Cancer. 2020 Apr 22;20(1):342.

275. Jansen P, Stoffels I, Müseler AC, Petri M, Brinker TJ, Schedlowski M, et al. Salivary cortisol levels and anxiety in melanoma patients undergoing sentinel lymph node excision under local anesthesia versus general anesthesia: a prospective study. World J Surg Oncol. 2020 Mar 10;18(1):53.

276. Salama AKS, Palta M, Rushing CN, Selim MA, Linney KN, Czito BG, et al. Ipilimumab and Radiation in Patients with High-risk Resected or Regionally Advanced Melanoma. Clin Cancer Res Off J Am Assoc Cancer Res. 2021 Mar 1;27(5):1287–95.

277. Sinnamon AJ, Gimotty PA, Karakousis GC, Yang YX. Survival Outcomes Following Lymph Node Biopsy in Thin Melanoma-A Propensity-Matched Analysis. Ann Surg Oncol. 2021 Mar;28(3):1634–41.

278. van Zeijl MCT, Ismail RK, de Wreede LC, van den Eertwegh AJM, de Boer A, van Dartel M, et al. Real-world outcomes of advanced melanoma patients not represented in phase III trials. Int J Cancer. 2020 Dec 15;147(12):3461–70.

279. von Schuckmann LA, Khosrotehrani K, Ghiasvand R, Hughes MCB, van der Pols JC, Malt M, et al. Statins may reduce disease recurrence in patients with ulcerated primary melanoma. Br J Dermatol. 2020 Dec;183(6):1049–55.

280. Asher N, Ben-Betzalel G, Lev-Ari S, Shapira-Frommer R, Steinberg-Silman Y, Gochman N, et al. Real World Outcomes of Ipilimumab and Nivolumab in Patients with Metastatic Melanoma. Cancers. 2020 Aug 18;12(8):2329.

281. Algazi A, Bhatia S, Agarwala S, Molina M, Lewis K, Faries M, et al. Intratumoral delivery of tavokinogene telseplasmid yields systemic immune responses in metastatic melanoma patients. Ann Oncol Off J Eur Soc Med Oncol. 2020 Apr;31(4):532–40.

282. Alves Wainstein AJ, Flores Ferrão EO, Virgílio Alves AC, Borges Murta MC, Drummond-Lage AP. Assessment of retraction in surgical specimens in melanoma patients submitted to oncological amplification of margins. Surg Oncol. 2021 Mar;36:106–12.

283. Liermann J, Winkler JK, Syed M, Neuberger U, Reuss D, Harrabi S, et al. Stereotactic Radiosurgery With Concurrent Immunotherapy in Melanoma Brain Metastases Is Feasible and Effective. Front Oncol. 2020;10:592796.

284. Gill AB, Rundo L, Wan JCM, Lau D, Zawaideh JP, Woitek R, et al. Correlating Radiomic Features of Heterogeneity on CT with Circulating Tumor DNA in Metastatic Melanoma. Cancers. 2020 Nov 24;12(12):3493.

285. Hadi I, Roengvoraphoj O, Bodensohn R, Hofmaier J, Niyazi M, Belka C, et al. Stereotactic radiosurgery combined with targeted/ immunotherapy in patients with melanoma brain metastasis. Radiat Oncol Lond Engl. 2020 Feb 14;15(1):37.

286. Curti B, Crittenden M, Seung SK, Fountain CB, Payne R, Chang S, et al. Randomized phase II study of stereotactic body radiotherapy and interleukin-2 versus interleukin-2 in patients with metastatic melanoma. J Immunother Cancer. 2020 May;8(1):e000773.

287. Holbrook K, Lutzky J, Davies MA, Davis JM, Glitza IC, Amaria RN, et al. Intracranial antitumor activity with encorafenib plus binimetinib in patients with melanoma brain metastases: A case series. Cancer. 2020 Feb 1;126(3):523–30.

288. Ponti A, Denys A, Digklia A, Schaefer N, Hocquelet A, Knebel JF, et al. First-Line Selective Internal Radiation Therapy in Patients with Uveal Melanoma Metastatic to the Liver. J Nucl Med Off Publ Soc Nucl Med. 2020 Mar;61(3):350–6.

289. Wong A, Callahan J, Keyaerts M, Neyns B, Mangana J, Aberle S, et al. 18F-FDG PET/CT based spleen to liver ratio associates with clinical outcome to ipilimumab in patients with metastatic melanoma. Cancer Imaging Off Publ Int Cancer Imaging Soc. 2020 May 14;20(1):36.

290. Verver D, Rekkas A, Garbe C, van Klaveren D, van Akkooi ACJ, Rutkowski P, et al. The EORTC-DeCOG nomogram adequately predicts outcomes of patients with sentinel node-positive melanoma without the need for completion lymph node dissection. Eur J Cancer Oxf Engl 1990. 2020 Jul;134:9–18.

291. Ellison PM, Zitelli JA, Brodland DG. Mohs micrographic surgery for melanoma: A prospective multicenter study. J Am Acad Dermatol. 2019 Sep;81(3):767–74.

292. Gonsalves CF, Eschelman DJ, Adamo RD, Anne PR, Orloff MM, Terai M, et al. A Prospective Phase II Trial of Radioembolization for Treatment of Uveal Melanoma Hepatic Metastasis. Radiology. 2019 Oct;293(1):223–31.

293. Phillips M, Marsden H, Jaffe W, Matin RN, Wali GN, Greenhalgh J, et al. Assessment of Accuracy of an Artificial Intelligence Algorithm to Detect Melanoma in Images of Skin Lesions. JAMA Netw Open. 2019 Oct 2;2(10):e1913436.

294. Luong J, Milanese E, Fortino J, Vetto JT. Reduction of lymphocele rate in patients undergoing sentinel node biopsy for melanoma by intraoperative placement of plant-based hemostatic powder: Results of a prospective trial. Am J Surg. 2019 May;217(5):878–81.

295. Bradbury KE, Appleby PN, Tipper SJ, Travis RC, Allen NE, Kvaskoff M, et al. Circulating insulin-like growth factor I in relation to melanoma risk in the European prospective investigation into cancer and nutrition. Int J Cancer. 2019 Mar 1;144(5):957–66.

296. Takayasu Y, Kubo N, Shino M, Nikkuni O, Ida S, Musha A, et al. Carbon-ion radiotherapy combined with chemotherapy for head and neck mucosal melanoma: Prospective observational study. Cancer Med. 2019 Dec;8(17):7227–35.

297. Forschner A, Battke F, Hadaschik D, Schulze M, Weißgraeber S, Han CT, et al. Tumor mutation burden and circulating tumor DNA in combined CTLA-4 and PD-1 antibody therapy in metastatic melanoma - results of a prospective biomarker study. J Immunother Cancer. 2019 Jul 12;7(1):180.

298. Hauswald H, Bernhardt D, Krug D, Katayama S, Habl G, Lorenzo Bermejo J, et al. Whole-brain helical tomotherapy with integrated boost for brain metastases in patients with malignant melanoma - final results of the BRAIN-RT trial. Cancer Manag Res. 2019;11:4669–76.

299. Henderson MA, Gyorki D, Burmeister BH, Ainslie J, Fisher R, Di Iulio J, et al. Inguinal and Ilio-inguinal Lymphadenectomy in Management of Palpable Melanoma Lymph Node Metastasis: A Long-Term Prospective Evaluation of Morbidity and Quality of Life. Ann Surg Oncol. 2019 Dec;26(13):4663–72.

300. Alvarez-Breckenridge C, Giobbie-Hurder A, Gill CM, Bertalan M, Stocking J, Kaplan A, et al. Upfront Surgical Resection of Melanoma Brain Metastases Provides a Bridge Toward Immunotherapy-Mediated Systemic Control. The Oncologist. 2019 May;24(5):671–9.

301. Banting S, Milne D, Thorpe T, Na L, Spillane J, Speakman D, et al. Negative Sentinel Lymph Node Biopsy in Patients with Melanoma: The Patient’s Perspective. Ann Surg Oncol. 2019 Jul;26(7):2263–7.

302. Czarnecka AM, Teterycz P, Mariuk-Jarema A, Lugowska I, Rogala P, Dudzisz-Sledz M, et al. Treatment Sequencing and Clinical Outcomes in BRAF-Positive and BRAF-Negative Unresectable and Metastatic Melanoma Patients Treated with New Systemic Therapies in Routine Practice. Target Oncol. 2019 Dec;14(6):729–42.

303. Ipenburg NA, Nieweg OE, Ahmed T, van Doorn R, Scolyer RA, Long GV, et al. External validation of a prognostic model to predict survival of patients with sentinel node-negative melanoma. Br J Surg. 2019 Sep;106(10):1319–26.

304. Lo MC, Heaton MJ, Snelling A, Moncrieff MD. Reconstructive burden and financial implications of wider excision margins for invasive primary cutaneous melanoma. J Plast Reconstr Aesthetic Surg JPRAS. 2020 Feb;73(2):313–8.

305. Lang N, Dick J, Slynko A, Schulz C, Dimitrakopoulou-Strauss A, Sachpekidis C, et al. Clinical significance of signs of autoimmune colitis in 18F-fluorodeoxyglucose positron emission tomography-computed tomography of 100 stage-IV melanoma patients. Immunotherapy. 2019 Jun;11(8):667–76.

306. Nijhuis AAG, Dieng M, Khanna N, Lord SJ, Dalton J, Menzies AM, et al. False-Positive Results and Incidental Findings with Annual CT or PET/CT Surveillance in Asymptomatic Patients with Resected Stage III Melanoma. Ann Surg Oncol. 2019 Jun;26(6):1860–8.

307. Jansen YJL, Rozeman EA, Mason R, Goldinger SM, Geukes Foppen MH, Hoejberg L, et al. Discontinuation of anti-PD-1 antibody therapy in the absence of disease progression or treatment limiting toxicity: clinical outcomes in advanced melanoma. Ann Oncol Off J Eur Soc Med Oncol. 2019 Jul 1;30(7):1154–61.

308. Grätz V, Zillikens D, Busch H, Langan EA, Terheyden P. Sequential Treatment With Targeted and Immune Checkpoint Therapy in Patients With BRAF Positive Metastatic Melanoma: The Importance of Timing? Front Med. 2019;6:257.

309. De Giorgi V, Grazzini M, Benemei S, Marchionni N, Botteri E, Pennacchioli E, et al. Propranolol for Off-label Treatment of Patients With Melanoma: Results From a Cohort Study. JAMA Oncol. 2018 Feb 8;4(2):e172908.

310. Forschner A, Keim U, Hofmann M, Spänkuch I, Lomberg D, Weide B, et al. Diagnostic accuracy of dermatofluoroscopy in cutaneous melanoma detection: results of a prospective multicentre clinical study in 476 pigmented lesions. Br J Dermatol. 2018 Aug;179(2):478–85.

311. Read TA, Smith A, Thomas J, David M, Foote M, Wagels M, et al. Intralesional PV-10 for the treatment of in-transit melanoma metastases-Results of a prospective, non-randomized, single center study. J Surg Oncol. 2018 Mar;117(4):579–87.

312. O’Leary FM, Beadsmoore CJ, Pawaroo D, Skrypniuk J, Heaton MJ, Moncrieff MD. Survival outcomes and interval between lymphoscintigraphy and SLNB in cutaneous melanoma- findings of a large prospective cohort study. Eur J Surg Oncol J Eur Soc Surg Oncol Br Assoc Surg Oncol. 2018 Nov;44(11):1768–72.

313. Dusingize JC, Olsen CM, Pandeya N, Thompson BS, Webb PM, Green AC, et al. Smoking and Cutaneous Melanoma: Findings from the QSkin Sun and Health Cohort Study. Cancer Epidemiol Biomark Prev Publ Am Assoc Cancer Res Cosponsored Am Soc Prev Oncol. 2018 Aug;27(8):874–81.

314. Amini-Adle M, Khanafer N, Le-Bouar M, Duru G, Dalle S, Thomas L. Ineffective anti PD-1 therapy after BRAF inhibitor failure in advanced melanoma. BMC Cancer. 2018 Jul 3;18(1):705.

315. Couto RA, Lamaris GA, Knackstedt R, Alleyne B, Durand P, Rueda S, et al. Determining the False-Negative Rate Using Fluorescence Image-Assisted Sentinel Lymph Node Biopsy in Cutaneous Melanoma. Ann Plast Surg. 2018 Jan;80(1):54–8.

316. Shoushtari AN, Friedman CF, Navid-Azarbaijani P, Postow MA, Callahan MK, Momtaz P, et al. Measuring Toxic Effects and Time to Treatment Failure for Nivolumab Plus Ipilimumab in Melanoma. JAMA Oncol. 2018 Jan 1;4(1):98–101.

317. Pameijer CR, Leung A, Neves RI, Zhu J. Indocyanine green and fluorescence lymphangiography for sentinel node identification in patients with melanoma. Am J Surg. 2018 Sep;216(3):558–61.

318. Du Four S, Janssen Y, Michotte A, Van Binst AM, Van den Begin R, Duerinck J, et al. Focal radiation necrosis of the brain in patients with melanoma brain metastases treated with pembrolizumab. Cancer Med. 2018 Oct;7(10):4870–9.

319. Mooradian MJ, Reuben A, Prieto PA, Hazar-Rethinam M, Frederick DT, Nadres B, et al. A phase II study of combined therapy with a BRAF inhibitor (vemurafenib) and interleukin-2 (aldesleukin) in patients with metastatic melanoma. Oncoimmunology. 2018;7(5):e1423172.

320. Read RL, Madronio CM, Cust AE, Goumas C, Watts CG, Menzies S, et al. Follow-Up Recommendations after Diagnosis of Primary Cutaneous Melanoma: A Population-Based Study in New South Wales, Australia. Ann Surg Oncol. 2018 Mar;25(3):617–25.

321. Si L, Zhang X, Xu Z, Jiang Q, Bu L, Wang X, et al. Vemurafenib in Chinese patients with BRAFV600 mutation-positive unresectable or metastatic melanoma: an open-label, multicenter phase I study. BMC Cancer. 2018 May 3;18(1):520.

322. Yao JJ, Zhang F, Zhang GS, Deng XW, Zhang WJ, Lawrence WR, et al. Efficacy and safety of primary surgery with postoperative radiotherapy in head and neck mucosal melanoma: a single-arm Phase II study. Cancer Manag Res. 2018;10:6985–96.

323. Kashani-Sabet M, Nosrati M, Miller JR, Sagebiel RW, Leong SPL, Lesniak A, et al. Prospective Validation of Molecular Prognostic Markers in Cutaneous Melanoma: A Correlative Analysis of E1690. Clin Cancer Res Off J Am Assoc Cancer Res. 2017 Nov 15;23(22):6888–92.

324. Rostas JW, Tam AL, Sato T, Scoggins CR, McMasters KM, Martin RCG. Health-related quality of life during trans-arterial chemoembolization with drug-eluting beads loaded with doxorubicin (DEBDOX) for unresectable hepatic metastases from ocular melanoma. Am J Surg. 2017 Nov;214(5):884–90.

325. Patel SP, Kim DW, Bassett RL, Cain S, Washington E, Hwu WJ, et al. A phase II study of ipilimumab plus temozolomide in patients with metastatic melanoma. Cancer Immunol Immunother CII. 2017 Oct;66(10):1359–66.

326. Williams NL, Wuthrick EJ, Kim H, Palmer JD, Garg S, Eldredge-Hindy H, et al. Phase 1 Study of Ipilimumab Combined With Whole Brain Radiation Therapy or Radiosurgery for Melanoma Patients With Brain Metastases. Int J Radiat Oncol Biol Phys. 2017 Sep 1;99(1):22–30.

327. Davies MA, Saiag P, Robert C, Grob JJ, Flaherty KT, Arance A, et al. Dabrafenib plus trametinib in patients with BRAFV600-mutant melanoma brain metastases (COMBI-MB): a multicentre, multicohort, open-label, phase 2 trial. Lancet Oncol. 2017 Jul;18(7):863–73.

328. de la Cruz-Merino L, Di Guardo L, Grob JJ, Venosa A, Larkin J, McArthur GA, et al. Clinical features of serous retinopathy observed with cobimetinib in patients with BRAF-mutated melanoma treated in the randomized coBRIM study. J Transl Med. 2017 Jun 24;15(1):146.

329. Montaudié H, Cerezo M, Bahadoran P, Roger C, Passeron T, Machet L, et al. Metformin monotherapy in melanoma: a pilot, open-label, prospective, and multicentric study indicates no benefit. Pigment Cell Melanoma Res. 2017 May;30(3):378–80.

330. Fabian ID, Stacey AW, Papastefanou V, Al Harby L, Arora AK, Sagoo MS, et al. Primary photodynamic therapy with verteporfin for small pigmented posterior pole choroidal melanoma. Eye Lond Engl. 2017 Apr;31(4):519–28.

331. Frankel AE, Eskiocak U, Gill JG, Yuan S, Ramesh V, Froehlich TW, et al. Digoxin Plus Trametinib Therapy Achieves Disease Control in BRAF Wild-Type Metastatic Melanoma Patients. Neoplasia N Y N. 2017 Apr;19(4):255–60.

332. Schreuer M, Jansen Y, Planken S, Chevolet I, Seremet T, Kruse V, et al. Combination of dabrafenib plus trametinib for BRAF and MEK inhibitor pretreated patients with advanced BRAFV600-mutant melanoma: an open-label, single arm, dual-centre, phase 2 clinical trial. Lancet Oncol. 2017 Apr;18(4):464–72.

333. Weide B, Martens A, Wistuba-Hamprecht K, Zelba H, Maier L, Lipp HP, et al. Combined treatment with ipilimumab and intratumoral interleukin-2 in pretreated patients with stage IV melanoma-safety and efficacy in a phase II study. Cancer Immunol Immunother CII. 2017 Apr;66(4):441–9.

334. Goldman C, Tchack J, Robinson EM, Han SW, Moran U, Polsky D, et al. Outcomes in Melanoma Patients Treated with BRAF/MEK-Directed Therapy or Immune Checkpoint Inhibition Stratified by Clinical Trial versus Standard of Care. Oncology. 2017;93(3):164–76.

335. Jakub JW, Terando AM, Sarnaik A, Ariyan CE, Faries MB, Zani S, et al. Safety and Feasibility of Minimally Invasive Inguinal Lymph Node Dissection in Patients With Melanoma (SAFE-MILND): Report of a Prospective Multi-institutional Trial. Ann Surg. 2017 Jan;265(1):192–6.

336. Saiag P, Gutzmer R, Ascierto PA, Maio M, Grob JJ, Murawa P, et al. Prospective assessment of a gene signature potentially predictive of clinical benefit in metastatic melanoma patients following MAGE-A3 immunotherapeutic (PREDICT). Ann Oncol Off J Eur Soc Med Oncol. 2016 Oct;27(10):1947–53.

337. Rossi M, Grassi R, Costa R, Di Rosa L, D’Arpa S, Moschella F, et al. Evaluation of the Upper Limb Lymphatic System: A Prospective Lymphoscintigraphic Study in Melanoma Patients and Healthy Controls. Plast Reconstr Surg. 2016 Dec;138(6):1321–31.

338. Hiniker SM, Reddy SA, Maecker HT, Subrahmanyam PB, Rosenberg-Hasson Y, Swetter SM, et al. A Prospective Clinical Trial Combining Radiation Therapy With Systemic Immunotherapy in Metastatic Melanoma. Int J Radiat Oncol Biol Phys. 2016 Nov 1;96(3):578–88.

339. Damude S, Hoekstra-Weebers JEHM, Francken AB, Ter Meulen S, Bastiaannet E, Hoekstra HJ. The MELFO-Study: Prospective, Randomized, Clinical Trial for the Evaluation of a Stage-adjusted Reduced Follow-up Schedule in Cutaneous Melanoma Patients-Results after 1 Year. Ann Surg Oncol. 2016 Sep;23(9):2762–71.

340. Rule WG, Allred JB, Pockaj BA, Markovic SN, DiCaudo DJ, Erickson LA, et al. Results of NCCTG N0275 (Alliance) - a phase II trial evaluating resection followed by adjuvant radiation therapy for patients with desmoplastic melanoma. Cancer Med. 2016 Aug;5(8):1890–6.

341. Eigentler TK, Gutzmer R, Hauschild A, Heinzerling L, Schadendorf D, Nashan D, et al. Adjuvant treatment with pegylated interferon α-2a versus low-dose interferon α-2a in patients with high-risk melanoma: a randomized phase III DeCOG trial. Ann Oncol Off J Eur Soc Med Oncol. 2016 Aug;27(8):1625–32.

342. Chen G, McQuade JL, Panka DJ, Hudgens CW, Amin-Mansour A, Mu XJ, et al. Clinical, Molecular, and Immune Analysis of Dabrafenib-Trametinib Combination Treatment for BRAF Inhibitor-Refractory Metastatic Melanoma: A Phase 2 Clinical Trial. JAMA Oncol. 2016 Aug 1;2(8):1056–64.

343. Krogh M, Christensen I, Bouwhuis M, Johansen JS, Nørgaard P, Schmidt H, et al. Prognostic and predictive value of YKL-40 in stage IIB-III melanoma. Melanoma Res. 2016 Aug;26(4):367–76.

344. Goff SL, Dudley ME, Citrin DE, Somerville RP, Wunderlich JR, Danforth DN, et al. Randomized, Prospective Evaluation Comparing Intensity of Lymphodepletion Before Adoptive Transfer of Tumor-Infiltrating Lymphocytes for Patients With Metastatic Melanoma. J Clin Oncol Off J Am Soc Clin Oncol. 2016 Jul 10;34(20):2389–97.

345. Fang S, Sui D, Wang Y, Liu H, Chiang YJ, Ross MI, et al. Association of Vitamin D Levels With Outcome in Patients With Melanoma After Adjustment For C-Reactive Protein. J Clin Oncol Off J Am Soc Clin Oncol. 2016 May 20;34(15):1741–7.

346. Weber J, Gibney G, Kudchadkar R, Yu B, Cheng P, Martinez AJ, et al. Phase I/II Study of Metastatic Melanoma Patients Treated with Nivolumab Who Had Progressed after Ipilimumab. Cancer Immunol Res. 2016 Apr;4(4):345–53.

347. McMasters KM, Egger ME, Edwards MJ, Ross MI, Reintgen DS, Noyes RD, et al. Final Results of the Sunbelt Melanoma Trial: A Multi-Institutional Prospective Randomized Phase III Study Evaluating the Role of Adjuvant High-Dose Interferon Alfa-2b and Completion Lymph Node Dissection for Patients Staged by Sentinel Lymph Node Biopsy. J Clin Oncol Off J Am Soc Clin Oncol. 2016 Apr 1;34(10):1079–86.

348. Zenda S, Akimoto T, Mizumoto M, Hayashi R, Arahira S, Okumura T, et al. Phase II study of proton beam therapy as a nonsurgical approach for mucosal melanoma of the nasal cavity or para-nasal sinuses. Radiother Oncol J Eur Soc Ther Radiol Oncol. 2016 Feb;118(2):267–71.

349. Kibrité A, Milot H, Douville P, Gagné ÉJ, Labonté S, Friede J, et al. Predictive factors for sentinel lymph nodes and non-sentinel lymph nodes metastatic involvement: a database study of 1,041 melanoma patients. Am J Surg. 2016 Jan;211(1):89–94.

350. Mohr P, Hauschild A, Trefzer U, Enk A, Tilgen W, Loquai C, et al. Intermittent High-Dose Intravenous Interferon Alfa-2b for Adjuvant Treatment of Stage III Melanoma: Final Analysis of a Randomized Phase III Dermatologic Cooperative Oncology Group Trial. J Clin Oncol Off J Am Soc Clin Oncol. 2015 Dec 1;33(34):4077–84.

351. Lawson DH, Lee S, Zhao F, Tarhini AA, Margolin KA, Ernstoff MS, et al. Randomized, Placebo-Controlled, Phase III Trial of Yeast-Derived Granulocyte-Macrophage Colony-Stimulating Factor (GM-CSF) Versus Peptide Vaccination Versus GM-CSF Plus Peptide Vaccination Versus Placebo in Patients With No Evidence of Disease After Complete Surgical Resection of Locally Advanced and/or Stage IV Melanoma: A Trial of the Eastern Cooperative Oncology Group-American College of Radiology Imaging Network Cancer Research Group (E4697). J Clin Oncol Off J Am Soc Clin Oncol. 2015 Dec 1;33(34):4066–76.

352. van Dijk EHC, van Herpen CML, Marinkovic M, Haanen JBAG, Amundson D, Luyten GPM, et al. Serous Retinopathy Associated with Mitogen-Activated Protein Kinase Kinase Inhibition (Binimetinib) for Metastatic Cutaneous and Uveal Melanoma. Ophthalmology. 2015 Sep;122(9):1907–16.

353. Speijers MJ, Bastiaannet E, Sloot S, Suurmeijer AJH, Hoekstra HJ. Tumor mitotic rate added to the equation: melanoma prognostic factors changed? : a single-institution database study on the prognostic value of tumor mitotic rate for sentinel lymph node status and survival of cutaneous melanoma patients. Ann Surg Oncol. 2015 Sep;22(9):2978–87.

354. Ferrucci PF, Minchella I, Mosconi M, Gandini S, Verrecchia F, Cocorocchio E, et al. Dacarbazine in combination with bevacizumab for the treatment of unresectable/metastatic melanoma: a phase II study. Melanoma Res. 2015 Jun;25(3):239–45.

355. Algazi AP, Cha E, Ortiz-Urda SM, McCalmont T, Bastian BC, Hwang J, et al. The combination of axitinib followed by paclitaxel/carboplatin yields extended survival in advanced BRAF wild-type melanoma: results of a clinical/correlative prospective phase II clinical trial. Br J Cancer. 2015 Apr 14;112(8):1326–31.

356. Bol KF, Mensink HW, Aarntzen EHJG, Schreibelt G, Keunen JEE, Coulie PG, et al. Long overall survival after dendritic cell vaccination in metastatic uveal melanoma patients. Am J Ophthalmol. 2014 Nov;158(5):939–47.

357. Malvehy J, Hauschild A, Curiel-Lewandrowski C, Mohr P, Hofmann-Wellenhof R, Motley R, et al. Clinical performance of the Nevisense system in cutaneous melanoma detection: an international, multicentre, prospective and blinded clinical trial on efficacy and safety. Br J Dermatol. 2014 Nov;171(5):1099–107.

358. Ribas A, Gonzalez R, Pavlick A, Hamid O, Gajewski TF, Daud A, et al. Combination of vemurafenib and cobimetinib in patients with advanced BRAF(V600)-mutated melanoma: a phase 1b study. Lancet Oncol. 2014 Aug;15(9):954–65.

359. Jiang BS, Beasley GM, Speicher PJ, Mosca PJ, Morse MA, Hanks B, et al. Immunotherapy following regional chemotherapy treatment of advanced extremity melanoma. Ann Surg Oncol. 2014 Aug;21(8):2525–31.

360. Fujiyama T, Oze I, Yagi H, Hashizume H, Matsuo K, Hino R, et al. Induction of cytotoxic T cells as a novel independent survival factor in malignant melanoma with percutaneous peptide immunization. J Dermatol Sci. 2014 Jul;75(1):43–8.

361. van der Ploeg APT, Haydu LE, Spillane AJ, Quinn MJ, Saw RP, Shannon KF, et al. Outcome following sentinel node biopsy plus wide local excision versus wide local excision only for primary cutaneous melanoma: analysis of 5840 patients treated at a single institution. Ann Surg. 2014 Jul;260(1):149–57.

362. Wilson MA, Zhao F, Letrero R, D’Andrea K, Rimm DL, Kirkwood JM, et al. Correlation of somatic mutations and clinical outcome in melanoma patients treated with Carboplatin, Paclitaxel, and sorafenib. Clin Cancer Res Off J Am Assoc Cancer Res. 2014 Jun 15;20(12):3328–37.

363. Kelderman S, Heemskerk B, van Tinteren H, van den Brom RRH, Hospers GAP, van den Eertwegh AJM, et al. Lactate dehydrogenase as a selection criterion for ipilimumab treatment in metastatic melanoma. Cancer Immunol Immunother CII. 2014 May;63(5):449–58.

364. Perier-Muzet M, Thomas L, Poulalhon N, Debarbieux S, Bringuier PP, Duru G, et al. Melanoma patients under vemurafenib: prospective follow-up of melanocytic lesions by digital dermoscopy. J Invest Dermatol. 2014 May;134(5):1351–8.

365. Shi F, Zhang X, Wu K, Gao F, Ding Y, Maharjan R, et al. Metastatic malignant melanoma: computed tomography-guided 125I seed implantation treatment. Melanoma Res. 2014 Apr;24(2):137–43.

366. Azer MWF, Menzies AM, Haydu LE, Kefford RF, Long GV. Patterns of response and progression in patients with BRAF-mutant melanoma metastatic to the brain who were treated with dabrafenib. Cancer. 2014 Feb 15;120(4):530–6.

367. Sanmamed MF, Fernández-Landázuri S, Rodríguez C, Lozano MD, Echeveste JI, Pérez Gracia JL, et al. Relevance of MIA and S100 serum tumor markers to monitor BRAF inhibitor therapy in metastatic melanoma patients. Clin Chim Acta Int J Clin Chem. 2014 Feb 15;429:168–74.

368. Yuan J, Zhou J, Dong Z, Tandon S, Kuk D, Panageas KS, et al. Pretreatment serum VEGF is associated with clinical response and overall survival in advanced melanoma patients treated with ipilimumab. Cancer Immunol Res. 2014 Feb;2(2):127–32.

369. Paulsen IF, Chakera AH, Drejøe JB, Klyver H, Dahlstrøm K, Oturai PS, et al. Tumour response after hyperthermic isolated limb perfusion for locally advanced melanoma. Dan Med J. 2014 Jan;61(1):A4741.

370. Romero AI, Chaput N, Poirier-Colame V, Rusakiewicz S, Jacquelot N, Chaba K, et al. Regulation of CD4(+)NKG2D(+) Th1 cells in patients with metastatic melanoma treated with sorafenib: role of IL-15Rα and NKG2D triggering. Cancer Res. 2014 Jan 1;74(1):68–80.
